# CD97 is associated with mitogenic pathway activation, metabolic reprogramming, and immune microenvironment changes in glioblastoma

**DOI:** 10.1038/s41598-022-05259-y

**Published:** 2022-01-27

**Authors:** Michael M. Safaee, Elaina J. Wang, Saket Jain, Jia-Shu Chen, Sabraj Gill, Allison C. Zheng, Joseph H. Garcia, Angad S. Beniwal, Y. Tran, Alan T. Nguyen, Melissa Trieu, Kevin Leung, Jim Wells, James M. Maclean, Keith Wycoff, Manish K. Aghi

**Affiliations:** 1 Department of Neurological Surgery, Brain Tumor Center, Helen Diller Comprehensive Cancer Center, University of California, San Francisco (UCSF), San Francisco, USA; 2 Planet Biotechnology, Inc., Hayward, CA USA; 3 School of Pharmacy, University of California, San Francisco (UCSF), San Francisco, USA

**Keywords:** CNS cancer, Tumour heterogeneity, Oncogenes

## Abstract

Glioblastoma (GBM) is the most common primary brain tumor with a median survival under two years. Using in silico and in vitro techniques, we demonstrate heterogeneous expression of CD97, a leukocyte adhesion marker, in human GBM. Beyond its previous demonstrated role in tumor invasion, we show that CD97 is also associated with upregulation of the mitogen-activated protein kinase/extracellular signal-regulated kinase (MAPK/Erk) and phosphatidylinositol 3-kinase/protein kinase B (PI3K/Akt) pathways in GBM. While CD97 knockout decreased Akt activation, CD97 targeting did not alter MAPK/Erk activation, did not slow GBM cell proliferation in culture, and increased levels of glycolytic and oxidative phosphorylation metabolites. Treatment with a soluble CD97 inhibitor did not alter activation of the MAPK/Erk and PI3K/Akt pathways. Tumors with high CD97 expression were associated with immune microenvironment changes including increased naïve macrophages, regulatory T cells, and resting natural killer (NK) cells. These data suggest that, while CD97 expression is associated with conflicting effects on tumor cell proliferative and metabolic pathways that overall do not affect tumor cell proliferation, CD97 exerts pro-tumoral effects on the tumor immune microenvironment, which along with the pro-invasive effects of CD97 we previously demonstrated, provides impetus to continue exploring CD97 as a therapeutic target in GBM.

## Introduction

Glioblastoma (GBM) is the most common primary malignancy of the brain with a median survival of less than 2 years^1^. Based on contemporary cohorts since the 2005 FDA approval of temozolomide chemotherapy for newly diagnosed GBM, the 2-year survival rate is 18% and 5-year survival rate is 4%^2^. There are numerous factors associated with this grim prognosis including the invasive nature of these tumors, rapid proliferation, radioresistance, tumoral heterogeneity, and an immunosuppressive tumor microenvironment^3–5^. Novel therapeutic targets are needed.

CD97 is a multi-functional leukocyte adhesion marker that has been implicated in GBM invasion. CD97 is a member of the EGF-TM7 family of adhesion G-protein coupled receptors (GPCRs) that are primarily expressed on the surface of leukocytes, but also on a subset of epithelial malignancies^6^. CD97’s role in thyroid, pancreatic, and colorectal cancers has been well studied; its expression correlates with local invasion, poor clinical staging, and angiogenesis^7–10^. In GBM, we previously demonstrated a role for CD97 in tumor migration and invasion, disproportionate expression of CD97 in the classical and mesenchymal subtypes of GBM, and a correlation between increased CD97 expression in GBM and shorter overall survival^11,12^.

Modern advances in single-cell sorting technology have allowed us to expand our analyses of tumors with greater granularity and resolution compared to traditional bulk RNA sequencing approaches. Studies evaluating the role of CD97 in GBM beyond our initial study in cell lines are limited. We therefore sought to utilize novel single-cell and bulk RNA sequencing approaches to investigate the association of CD97 expression with mitogenic pathways, metabolic pathways, and the tumor immune microenvironment.

## Results

### CD97 is heterogeneously expressed in GBM

**Figure 1:**
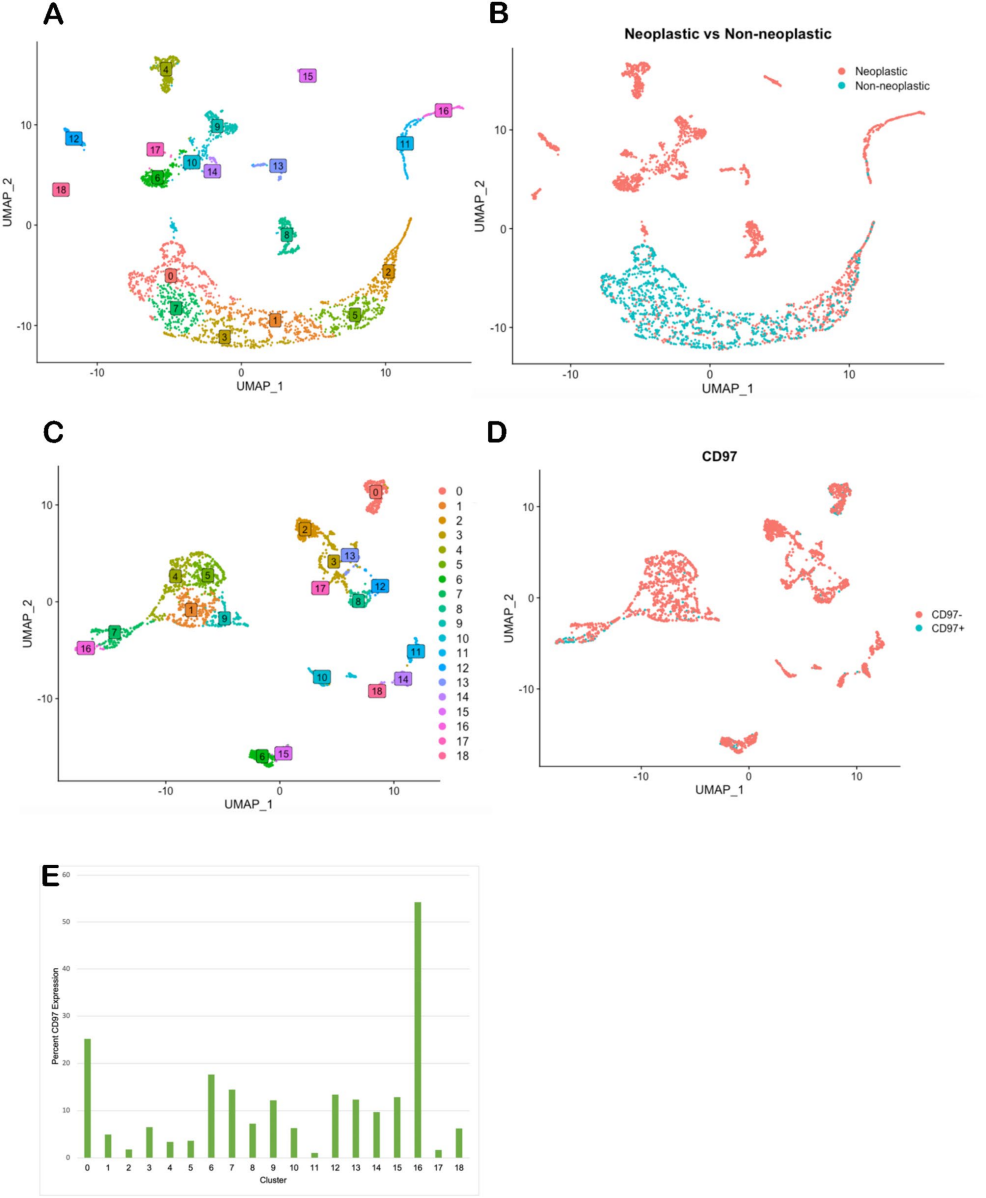
Single-cell analysis of CD97-expressing glioblastoma cells. Glioblastoma (GBM) cells from 14 IDH-wild type cases were subjected to single-cell analysis. (**A**) Shown is a t-SNE plot classifying cells from these 14 cases into 19 clusters. (**B**) Neoplastic versus non-neoplastic cells were identified and highlighted in pink via copy number variation (CNV) analysis, with stromal cells shown in cyan. (**C**) Neoplastic cells (n = 2530) were clustered into 19 distinct cell populations based off differential gene expression. (**D**) CD97^+^ and CD97^−^ cells are highlighted among neoplastic cells from these 19 clusters from 14 GBM patient samples. (**E**) Percentage of CD97^+^ cells among the 19 clusters derived from 2,530 tumor cells.

We analyzed a previously published^13^ single cell RNA-seq (scRNA-seq) performed on 3550 cells from 14 human IDH-wild type GBM specimens. Cells were clustered according to principal component analysis (PCA), resulting in 19 unique populations (Fig. 1A). Cells were classified as tumor cells or stromal cells based on copy number variation (CNV) analysis, revealing 2530 neoplastic cells (71.3%) and 1020 non-neoplastic cells (28.7%) (Fig. 1B). PCA allowed us to group neoplastic cells into 19 clusters (Fig. 1C). These neoplastic cells were then classified as CD97^+^ (n = 262 = 10.4%) or CD97^−^ (n = 2268 = 89.6%) based on the presence or absence of CD97 RNA, respectively (Fig. 1D). Mean percent CD97^+^ cells amongst all clusters was 11.3% with a distribution ranging from 1 to 54% (Fig. 1E).

### Gene expression changes associated with CD97 expression in GBM cells reveal targetable expression and activation of cell proliferation pathways

**Figure 2:**
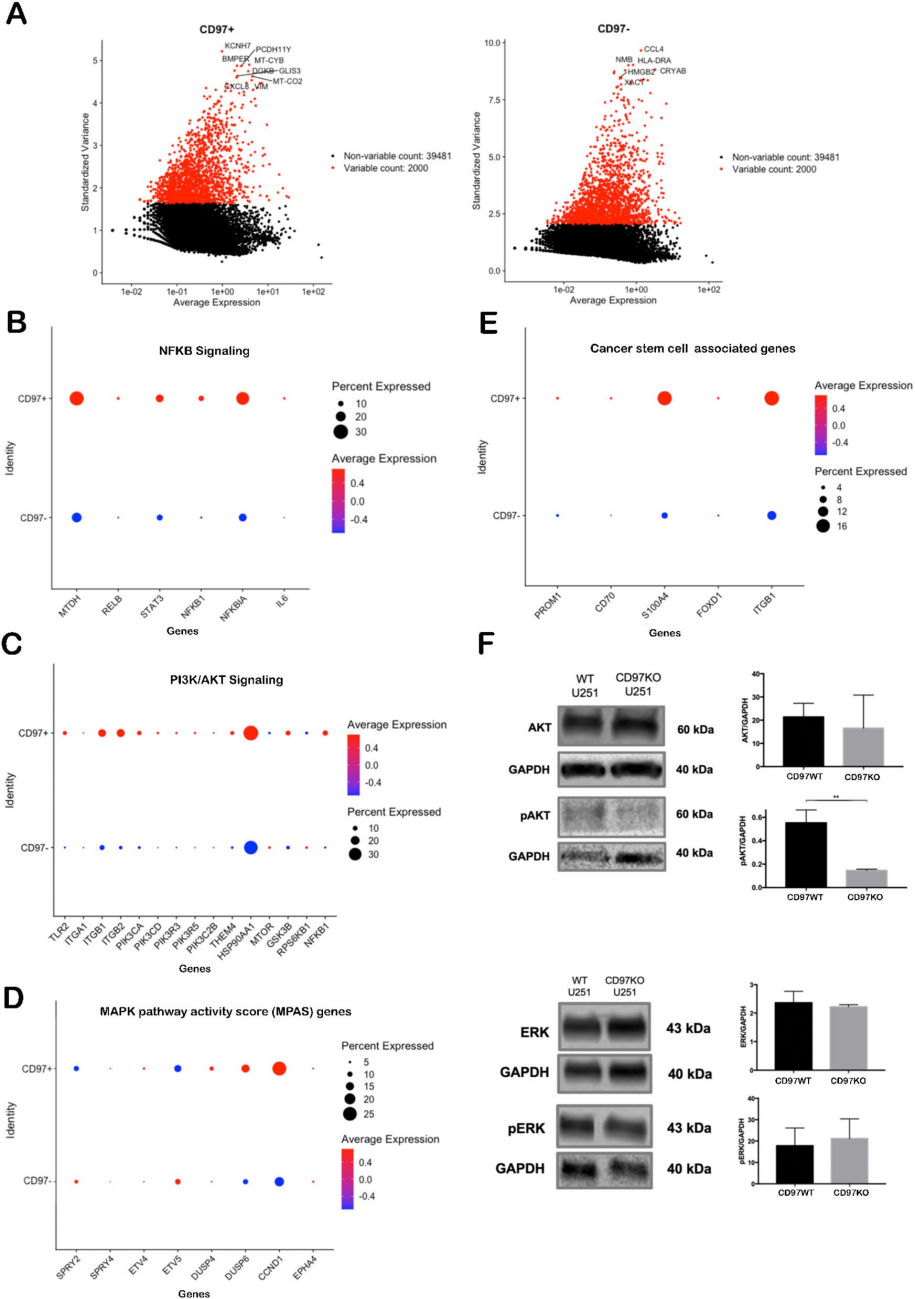
Proliferative pathways are activated in CD97-expressing patient glioblastoma cells and are suppressed with CD97 knockdown in a human GBM cell line. Proliferative pathways are activated in CD97-expressing patient GBM cells (**A–D**). **(A)** Variable feature plots illustrating the top 10 differentially expressed genes in CD97^+^ cells versus CD97^-^ cells demonstrate a bias toward NFKB pathway in CD97^+^ cells and higher expression of immune genes in CD97^-^ cells. **(B)** Genes associated with NFKB signaling were upregulated in CD97^+^ cells compared to CD97^-^ cells. **(C)** CD97 + tumor cells had higher expression of several receptors that utilize PI3K/Akt signaling, cytoplasmic activators of Akt, and downstream targets of Akt. (**D**) CD97^+^ tumor cells had higher expression of genes associated with the MAPK pathway activity score (MPAS) than CD97^-^ tumor cells. (**E**) CD97^+^ tumor cells had higher expression of genes associated with GBM stem cells than CD97^-^ tumor cells. (**F**) WT U251 cells demonstrate higher protein expression of phosphorylated AKT (pAKT) compared to CD97KO U251 cells (P < 0.01), while AKT, ERK, and phosphorylated ERK (pERK) did not demonstrate difference in protein expression between the two groups. Full western blots are shown in Supplementary Figs. S12 and S13. *P < 0.05, **P < 0.01, ***P < 0.001.

We then defined the 2000 most highly variable genes in CD97^+^ and CD97^-^ tumor cells (Supplementary Table S1; Fig. 2A). These genes demonstrated a bias toward NFκB pathway genes such as KCNH7 in CD97^+^ cells and higher expression of immune response regulating genes such as CCL4 and HLA-DRA in CD97- cells.

We then analyzed expression of 6 NF-κB pathway genes and found them all to be elevated in CD97^+^ GBM cells compared to CD97^-^ GBM cells (Fig. 2B). Because the NF-κB signaling cascade interacts with the signaling cascades initiated by PI3K/Akt^14^ and the large MAPK family^15^ and because of literature demonstrating that CD97 signaling increases Erk and MAPK activation in prostate^16^ and thyroid^17^ cancers, we investigated these pathways in CD97^+^ GBM cells. CD97^+^ tumor cells had higher expression of several receptors that utilize PI3K/Akt signaling, cytoplasmic activators of Akt, and downstream targets of Akt (Fig. 2C). CD97^+^ tumor cells had higher expression of genes associated with the MAPK pathway activity score (MPAS)^18^ than CD97^-^ tumor cells (Fig. 2D). These CD97^+^ GBM cells which expressed genes associated with these proliferative pathways also expressed stem cell markers^19,20^ more than CD97^-^ GBM cells (Fig. 2E), which was consistent with our previous demonstration that CD97 is expressed by GBM stem cells^12^. And consistent with our prior demonstration of CD97 promoting GBM cell invasion, CD97^+^ GBM cells expressed mediators of invasion^21^ at higher levels than CD97^-^ GBM cells (Supplementary Fig. S1).

To corroborate our findings of increased proliferative pathway gene expression in CD97^+^ GBM cells compared to CD97^-^ GBM cells, we analyzed the impact of reducing CD97 expression in human GBM cells on these pathways. U251 GBM cells were transfected with Synthego CRISPRevolution sgRNA to create the CD97 knockout cells. CD97 expression in CD97 knockout cells was verified using quantitative PCR (qPCR) (P < 0.001; Supplementary Fig. S2A) and flow cytometry (Supplementary Fig. S2B). In parallel, CRISPRi/dCas9-KRAB-MeCP2 and gRNAs targeting CD97 (dCAS9KRAB-MeCP2-CD97sgRNA) were transduced into U251 GBM cells, to create U251 control (U251-dCas9-KRAB-MeCP2) and U251 CD97 knockdown (U251-dCAS9-KRAB-MeCP2-CD97sgRNA) cells. Knockdown efficiency was verified using flow cytometry (Supplementary Fig. S3).

CD97 knockout in U251 human GBM cells lowerd phosphorylation of Akt, but not MAPK (Fig. 2F). However, these changes did not impact proliferation of these GBM cells in U251 cells with CD97 knockout or knockdown (Supplementary Fig. S4). We then treated U251 cells with a soluble inhibitor of CD97. This agent, DAF-Fc, is comprised of decay accelerating factor (DAF), also known as CD55, a known ligand of CD97 fused to the human IgG1 Fc region, creating an Fc-fusion protein with high binding affinity for CD97 (Supplementary Fig. S5). U251 tumor cells were treated with DAF-Fc in culture and then lysed 24 h after treatment. Western Blot analysis showed unchanged levels of phosphorylated Erk and Akt (Supplementary Fig. S6).

### Ontologic analysis of CD97^+^ GBM cells, tumors, and cell lines

**Figure 3:**
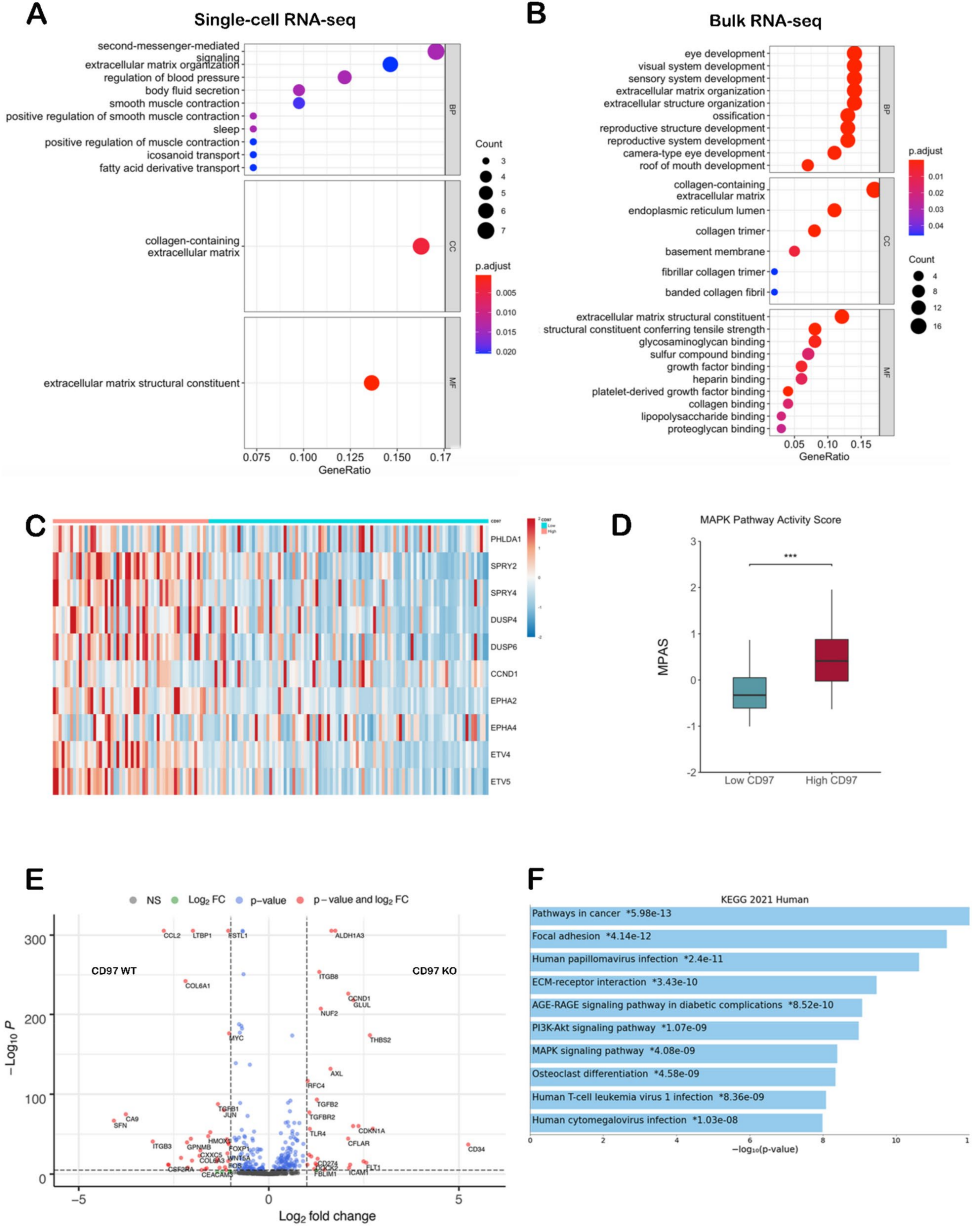
Transcriptional programs activated in CD97-expressing GBM cells and in CD97^hi^ patient GBMs. (**A**) Ontologic analysis of the top 25 genes with higher expression in CD97^+^ versus CD97^-^ single GBM cells from 14 patient GBMs reveal associations with second-messenger signaling and extracellular matrix organization. (**B**) Ontologic analysis of the 133 genes with fold change > 2 and adjusted P < 0.05 in bulk RNA-seq of CD97^hi^ GBMs relative to CD97^lo^ GBMs revealed that upregulated genes were associated with pathways involved in extracellular matrix organization, extracellular structure organization, and glycosaminoglycan binding. (**C,D**) Heatmap (**C**) and bar plot (**D**) showing expression of the 10 genes that comprise the MPAS gene signature in bulk RNA-seq data across 151 patients reveals significantly higher expression of MPAS genes in CD97^hi^ versus CD97^lo^ patient GBMs. (**E**) Volcano plot showing differentially expressed genes in U251 cells with CD97 knockout (CD97 KO) versus wild-type U251 (CD97 WT) cells. Genes on the left are upregulated in CD97 WT/downregulated in CD97 KO, while genes on the right are upregulated in CD97 KO/downregulated in CD97 KO. (**F**) Compared to wild-type U251 GBM cells, those with CD97 knockout demonstrated significant reduction in gene expression associated with pathways related to cancer, focal adhesion, PI3K pathway, and ERK/MAPK pathways. *P < 0.05; **P < 0.01; ***P < 0.001.

We then supplemented these findings of individual gene expression changes from scRNA-seq with pathway analysis. Ontologic analysis of the top 25 genes with higher expression in CD97^+^ versus CD97^-^ single cells (Supplementary Fig. S7A) reveal associations with second-messenger signaling and extracellular matrix organization. (Fig. 3A).

To build upon this scRNA-seq data using bulk RNA-seq in a larger cohort, 151 GBM specimens from The Cancer Genome Atlas (TCGA) were stratified into CD97^hi^ and CD97^lo^ tumors based on mean expression values. Using RNA sequencing data from bulk GBM tumor, ontologic analysis was performed on CD97^hi^ vs. CD97^lo^ tumors. Upregulated genes in these tumors were associated with extracellular matrix organization, extracellular structure organization, and glycosaminoglycan binding (Fig. 3B). The upregulated genes implicated in the enriched extracellular matrix and structure organization processes include several genes from the collagen superfamily (COL2A1, COL5A1, COL6A1, COL6A2, COL6A3, COL9A3, COL12A1, COL13A1), ADAM19, CREB3L1, FAP, FBN2, POSTN, and PDGFA (Supplementary Fig. S7B). Consistent with our scRNA-seq results, analysis of the MPAS gene signature in TCGA bulk RNA-seq data across 151 patients revealed significantly higher expression of MPAS genes in CD97^hi^ versus CD97^lo^ patients (Fig. 3C,D).

We then examined the effect of CD97 knockout in a cultured human GBM cell line on pathways. U251-WT and U251-CD97 knockout cells were assessed in the NanoString nCounter platform using a 780 gene multiplex related to over 40 pathways involved in tumor signaling. Genes whose expression was downregulated in knockout cells relative to wild-type included pro-invasive genes *COL6A1*^22^, *ITGB3*^23^, and *CA9*^24^, along with the gene *CCL2* which is implicated in regulatory T cell (T_reg_) recruitment and, although mostly produced by immune cells, is also produced by GBM cells after PI3K/Akt and MAPK pathway activation^25^ (Fig. 3E, Supplementary Figs. S8–S10; Supplementary Tables S2–S3). Enrichr software was then used to analyze expression of pathways defined in the KEGG 2019 Human database in this multiplex Nanostring analysis of U251 control cells and U251 CD97 knockout cells. Pathways downregulated in knockout cells relative to wild-type included ECM-receptor interaction, PI3K/Akt signaling, and MAPK signaling (Fig. 3F).

### Metabolic analysis of CD97^+^ GBM tumor cells

Because previous reports have provided mixed findings on the impact of activating these proliferative pathways on tumor cell metabolism^26^, we then assessed the effect of CD97 knockdown on GBM cell metabolism. Metabolomic assessment after cells were incubated in a low concentration (0.1 g/L) of ^13^C_6_-glucose revealed that U251 control (U251-dCas9-KRAB-MeCP2) cells had less glycolytic (Supplementary Fig. S11A) and TCA (Supplementary Fig. S11B) metabolites than U251 CD97 knockdown (U251-dCAS9-KRAB-MeCP2-CD97sgRNA) cells, suggesting that, despite the energy requirements associated with the proliferative pathways it activates, in GBM cells, CD97 slowed metabolic flux.

### Effect of CD97 expression on the immune microenvironment

**Figure 4:**
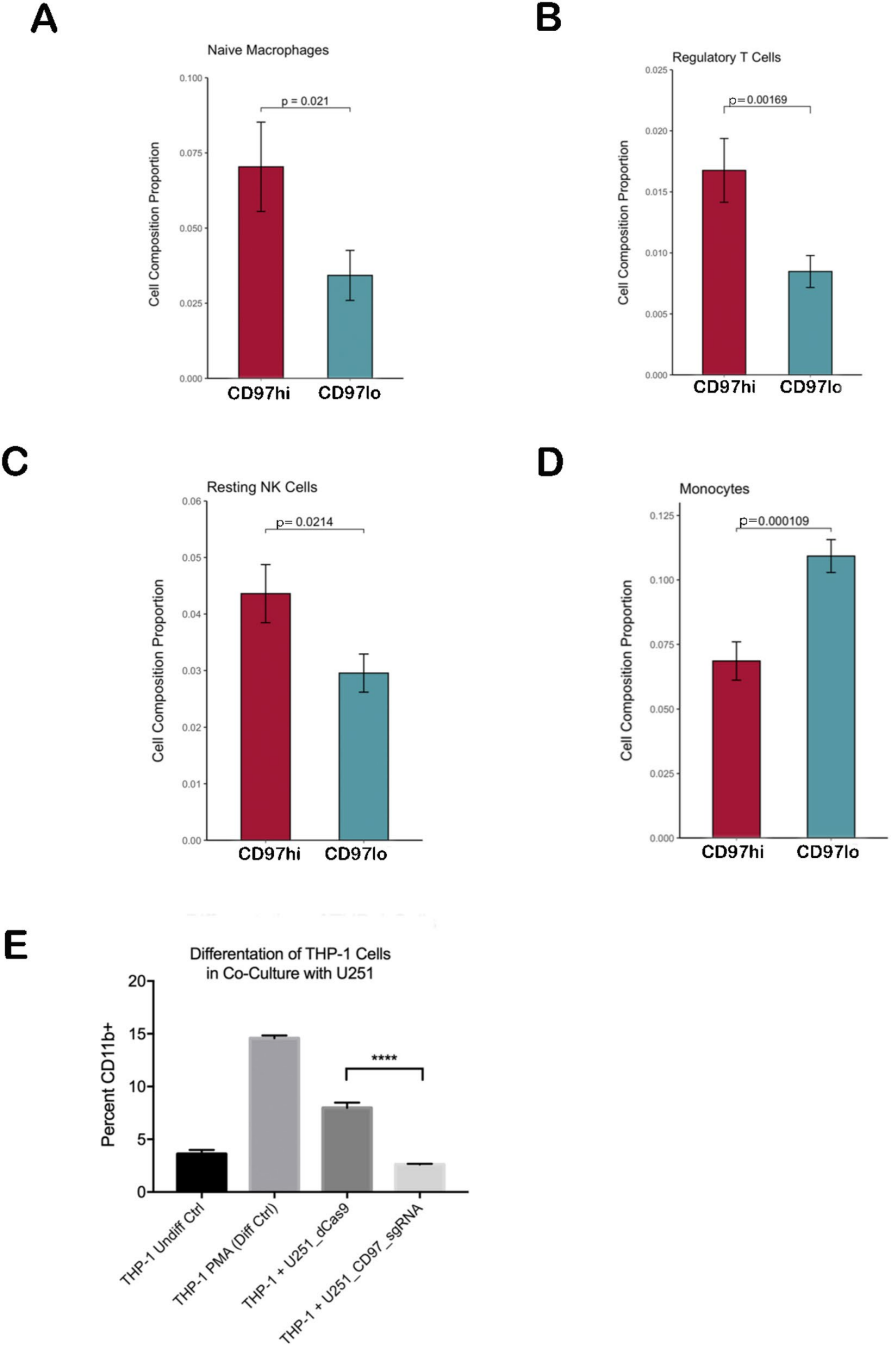
CD97 alters the GBM immune microenvironment. Compared to CD97^lo^ GBMs, CD97^hi^ GBM tumors exhibited significantly greater proportion of (**A**) nonpolarized M0 macrophages, (**B**) regulatory T cells, and (**C**) resting NK cells. In contrast, compared to CD97^hi^ GBM tumors, CD97^lo^ GBM tumors had higher proportions of (**D**) monocytes. (**E**) THP-1 monocytes co-cultures with wild type U251 GBM cells had significantly higher macrophage differentiation compared to those co-cultured with U251 CD97-knockdown cells (P < 0.001). *P < 0.05; **P < 0.01; ***P < 0.001.

To specifically isolate the impact of CD97 expression on immune cells in the tumor microenvironment, we performed deconvolution of bulk GBM RNA-seq data from TCGA via CIBERSORT to specifically identify and quantify 22 human hematopoietic cell phenotypes, including naïve and memory B cells, plasma cells, NK cells, myeloid subsets, and seven different T cell subtypes based on the expression of 547 genes included in the LM22 gene signature^27^. Three cell types represented a significantly greater proportion of the immune cell population in CD97^hi^ tumors compared to CD97^lo^ tumors: nonpolarized M0 macrophages (0.070 vs. 0.034, p = 0.021; Fig. 4A), immunosuppressive T_reg_ cells (0.017 vs. 0.008, p = 0.002; Fig. 4B), and resting NK cells (0.044 vs. 0.029, p = 0.02; Fig. 4C). Conversely, the proportion of monocytes was significantly greater in CD97^lo^ tumors relative to CD97^hi^ tumors (0.109 vs. 0.069, p < 0.001; Fig. 4D). Other cell types, including M1 anti-tumoral macrophages, M2 pro-tumoral macrophages, CD8^+^ cytotoxic T cells, naïve CD4^+^ helper T cells, resting CD4^+^ memory T cells, activated CD4^+^ memory T cells, plasma cells, and activated NK cells did not change between CD97^hi^ tumors vs. CD97^lo^ tumors (P = 0.1–0.7; Supplementary Table S4).

To corroborate this data in culture, we compared monocyte differentiation in the presence of CD97^+^ and CD97^−^ tumor cells. The THP-1 immortalized monocyte cell line was co-cultured with either U251 wildtype (U251-dCas9-KRAB-MeCP2) or U251 CD97 knockdown (U251-dCAS9-KRAB-MeCP2-CD97sgRNA) cells. There was significantly greater differentiation of monocytes to macrophages among THP-1 cells co-cultured with U251 wild type cells, which express high levels of CD97 (Fig. 4E).

## Discussion

Intra- and intertumoral heterogeneity is a significant impediment towards effective therapy against GBM. Advances in single-cell RNA-sequencing have transformed our ability to investigate intratumoral heterogeneity within GBM, while the large amount of bulk RNA-sequencing deposited through the TCGA allows for detailed insights into intertumoral heterogeneity. In this study, we perform the first analysis using both single-cell and bulk RNA sequencing analysis to analyze intra- and intertumoral CD97 expression heterogeneity in GBM and examine the gene expression pathways activated in individual CD97-expressing vs. CD97-non-expressing tumor cells and in CD97-high vs. CD97-low patient GBMs.

By integrating single cell and bulk RNA sequencing, our work combines the strength of single cell RNA-seq in identifying gene expression changes occurring in small subsets of cells with the strength of bulk RNA-seq in identifying high fold-change differentially expressed genes^28^. The use of single-cell techniques combined with CNV analysis allowed us to distinguish CD97 expressed by tumor cells from CD97 expressed by GBM stromal cells known to express CD97, such as lymphocytes, monocytes, macrophages, dendritic cells, and granulocytes^29^.

The single cell and bulk RNA sequencing analyses performed in this study suggested that CD97 expression within GBM cells is associated with more than simply the phenotypic differences in migratory and invasive capacity that we previously described^11,12^. Our previous work showed that CD97 upregulation is associated with worse survival in GBM^11^, but it was unclear if that correlation with survival reflected the pro-invasive effects of CD97 we described or other functions of CD97 not yet defined in GBM.

Non-invasive proliferative functions of CD97 mediated by its association with Gα12/13 have been described in other cancers. In addition to phenotypic differences, Ward et al. demonstrated increased Rho-GTP levels in prostate cancer through signaling mediated by CD97 and its association with lysophosphatidic acid receptor 1 (LPAR1) and subsequent ERK activation^16^. CD97 signaling has also been shown to increase MAPK activation in thyroid^17^ cancers. CD97 can even mediate cellular proliferation in non-neoplastic cells, such as in intestinal epithelial cells where this occurs through increased Akt activation^30^. We corroborated these findings in GBM by demonstrating that CD97^+^ single-cell clusters had increased transcription of genes involved in the ERK/MAPK and PI3K/AKT pathways than CD97^-^ clusters, patient GBMs with overall highest levels of CD97 expression had activation of similar pathways, and CD97 knockout in a GBM cell line decreased PI3K/AKT activation.

Despite these findings, we also found that CD97 knockout or knockdown did not slow proliferation of cultured GBM cells and that treatment of these cultured GBM cells with a CD97 inhibitor did not affect the activation of these proliferative pathways. There are several possible explanations for this discrepancy. First, our RNA-seq revealed that CD97 was only expressed by a minority of GBM cells and these cells could be a subset of tumor cells, such as the GBM stem cells whose markers were enriched in these CD97^+^ cells, with altered machinery that allows CD97 to drive proliferation in a way not captured by a CD97-expressing GBM cell line. Second, our finding that CD97 knockdown accelerated metabolic flux through the glycolytic and oxidative phosphorylation pathways means that CD97 could slow tumor cell metabolism, an effect that could counteract the proliferative pathways we found CD97 to activate. Third, since CD97 is a transmembrane protein that interacts with extracellular ligands, the discrepancy between our results in patient specimens and cultured cells could be due to differences in how CD97 interacts with the tumor microenvironment in patient GBMs compared to homogeneous cells in culture.

The poor prognosis of GBM likely reflects the capacity of GBM cells to activate proliferative and invasive programs. Proliferation leads to expansile tumor growth, causing mass effect on surrounding structures. Invasion enables escape from surgical resection and drives inevitable recurrence, with over 90% of recurrences occurring as local continuous growth 2 cm from the original tumor^31–36^. This combination of proliferation and invasion ultimately proves fatal as tumor cells compress or invade the surrounding brain. There is thus a need to target both invasion and proliferation in GBM, as, at the microscopic level, proliferation and migration appear to be temporally, mutually exclusive phenotypes that toggle back and forth, known as the “go or grow” principle^37^. By positioning CD97 at the interface between proliferative and invasive programs in GBM, our findings support CD97 as a therapeutic target in GBM.

However, validating CD97 as a therapeutic target in GBM will require accounting for the three different CD97 isoforms and the three known CD97 ligands. The three different isoforms of CD97 are formed through differential splicing to incorporate 3, 4, or 5 EGF domains: CD97 (EGF 1,2,5), CD97 (EGF 1,2,3,5), or CD97 (EGF 1–5), of which CD97 (EGF 1,2,3,5) and CD97 (EGF 1,2,5) are the two expressed in GBMs^12^, with evidence for distinct functions of each^38,39^. CD97 also has three known ligands: decay accelerating factor (DAF/CD55), a regulator of the complement cascade; chondroitin sulfate B; and α5β1 and αvβ3 integrins. Because chondroitin sulfate B requires the EGF domain 4 of CD97, which is not present in GBM^12^, the biology of CD97 in GBM will involve its binding to DAF/CD55 and/or integrins α5β1 and αvβ3 which are transmembrane proteins that would mediate cell to cell interactions. Because these transmembrane ligands have been shown to activate Erk and Akt, the pathways we identified as activated in CD97-expressing GBM cells, we cannot draw conclusions about which ligands are used by CD97 in GBM based on our signaling findings. We can, however, narrow down which ligands GBM cell CD97 bind to based on their expression pattern. For example, the effect of CD97 on GBM immune cell populations is likely through DAF/CD55 because integrins α5β1 and αvβ3 are not expressed by leukocytes^40^. Effects of CD97 on GBM proliferation and invasion are interesting because cell-to-cell interactions promoting these processes are less well described in the literature. Further study will be needed to determine which ligand mediates these processes in GBM and whether they occur through interactions between GBM tumor cells or between GBM tumor cells and GBM stromal cells.

Even if the discrepancy we found between CD97 and proliferation in patient specimens versus a cell line means that the correlation between CD97 and GBM cell proliferation is not meaningful, our findings of pro-tumoral effects of CD97 on the GBM immune microenvironment and our previously demonstrated findings of invasive roles for CD97, which were supported by our ontologic analysis, provides impetus to continue exploring CD97 as a potential therapeutic target in GBM.

While our findings about the effects of CD97 on the immune microenvironment of GBM emerged from bulk RNA-seq in which the source of CD97 expression cannot be distinguished between GBM cells versus stromal cells the way it could for scRNA-seq, our cell line conditioned media data suggested that some of these findings arise from GBM cell CD97, suggesting an intriguing previously unrecognized role for GBM cell CD97 in regulating the immunologic microenvironment of GBM. CD97 and DAF are both expressed on antigen-presenting cells and T-cells and may facilitate T-cell activation in a bidirectional manner^41^, and GBM cell expression of CD97 may facilitate similar interactions with DAF expressed by antigen-presenting cells or T-cells.

There are several limitations to this work, namely those inherent to RNA sequencing analysis and cell line studies. Future studies could expand upon our findings of the cellular heterogeneity of GBM CD97 expression in a spatial manner using recently described spatial single-cell gene expression profiling platforms^42^. Additional studies are also needed to explore whether CD97 inhibition could be effective against the subset of patient GBM cells that express CD97 in a manner that we did not see with cultured GBM cells and whether inhibiting CD97 in GBM should be pursued by targeting one of the two CD97 isoforms expressed by GBM rather than targeting both. These studies will be needed to determine if the effects of CD97 on mitogenic pathways and the immune microenvironment in GBM that we identified can be translated to provide a potential therapeutic target for an otherwise fatal disease.

## Methods

### Single cell analysis

Feature-barcode matrices that had undergone the 10× CellRanger pipeline representing 14 adult IDH-wild type GBM specimens from UCSF were downloaded from the Gene Expression Omnibus (GSE138794)^13^. Data analysis was performed in R using Seurat v3.0^43^. Cells with at least 200 features and features expressed in at least 3 cells were included. Percent mitochondrial gene expression threshold was set to be under 10%. Normalization of gene expression was conducted according to the ‘LogNormalize’ function provided in the Seurat workflow. Feature selection, dimensional reduction, and cluster identification were conducted using the provided commands in the Seurat package.

Doublets were predicted and removed from the dataset using DoubletFinder^44^. Copy number variation (CNV) analysis was performed using the CONICS package^45^ to identify tumor cells. Neoplastic cells were classified as CD97^+^ or CD97^−^ based on presence or absence of CD97 expression in the raw data. Two-proportions Z-tests were conducted via the ‘prop.test’ function in R to determine significance.

CD97^+^ and CD97^−^ subsequently underwent normalization, feature selection, dimensional reduction, and cluster identification in parallel. The 2000 most variable genes were identified using variance stabilizing transformation as provided by the Seurat workflow. Dot plots were generated using the ‘DotPlot’ function in Seurat. Default values were used for all quantitative parameters unless otherwise noted.

### Bulk RNA sequencing analysis

Gene expression data (HTSeq counts) and clinical information from the TCGA GBM cohort were downloaded via the R package *TCGAbiolinks*^46^. Primary GBM tissue samples were included in the analysis while recurrent tumor and normal brain tissue specimens were removed. The *edgeR* package^47^ was used to convert gene counts into counts per million (CPM), and only genes with CPM > 0.5 in more than 2 samples were retained in order to filter out lowly expressed genes. Samples were dichotomized into 2 subgroups on the basis of CD97 expression (Cd97^hi^ and CD97^lo^), with the mean CD97 expression of the cohort used as the threshold for dichotomization. Differential gene expression analysis between the high and low CD97 groups was performed using *DESeq2*^48^, with differentially expressed genes (DEG) defined as those with a Benjamini–Hochberg adjusted *p*-value < 0.05 and a |log_2_Fold Change|> 1. DEG symbols were mapped to their Entrez gene IDs using Bioconductor’s *AnnotationDbi* and *org.Hs.eg.db* packages and subsequently used for Gene Ontology (GO) enrichment analysis through the *clusterProfiler* package^49^. Enriched GO gene sets were visualized as dot and gene-concept network plots using the *enrichPlot* package.

TCGA bulk RNA-seq data for GBM was also downloaded from the Broad GDAC Firehose website (http://gdac.broadinstitute.org/) in order to access RSEM scaled estimates for the gene expression levels^50^. The RSEM algorithm uses a directed graph model and the expectation maximization algorithm to provide scaled estimates, which can be multiplied by 10^6^ to derive transcript abundance in terms of transcripts per million (TPM). The TPM data of the GBM samples were inputted into the CIBERSORT web application (https://cibersort.stanford.edu/), a deconvolution algorithm that uses support vector regression to infer the relative proportions of 22 types of infiltrating immune cells from bulk tumor sample expression data^27^. The LM22 markers derived from purified distinct immune cell populations and 100 permutations were used in CIBERSORT to enumerate the abundance of immune cells in each sample. Using the previously described CD97 labels, tumor-infiltrating leukocyte composition was compared between the two CD97 groups using the Mann–Whitney U test. A two-tailed p-value < 0.05 was used as the threshold for significance.

### Cell culture

Human U251 (American Type Culture Collection) GBM cells were grown in Dulbecco’s Modified Essential Media (DMEM) with 4.5 g/L glucose, 0.584 g/L L-glutamine, 0.11 g/L sodium pyruvate, 3.7 g/L NaHCO_3_, 10% FBS, and 1% penicillin/streptomycin (complete DMEM). DAF-Fc was kindly provided by Planet Biotechnology (Hayward, CA).

### CRISPRi knockdown cell lines

A lentiviral plasmid containing a dCAS9/KRAB/MeCP2 cassette was obtained from Addgene. Lenti-X 293 T cells were transfected using this plasmid and virus was generated and appropriate titers were determined. U251 cells were then transduced with the virus and selected using 5 µg/ml blasticidin to obtain a pure dCAS9/KRAB/MeCP2 positive population. U251 cells expressing dCAS9/KRAB/MeCP2 were then transfected with the plasmid containing the sgRNA GAGACGCGGCCTCCCATGGT targeting CD97. Cells were selected using 5 µg/ml Blasticidin and 5 µg/ml Puromycin.

### CRISPR knockout cell lines

Knockout cells were prepared from U251 cells according to the Synthego CRISPRevolution sgRNA EZ Kit with sequence ACCCCGACGGAGACUUGUGA. After culturing, a single cell sort was performed using a Sony SH800 FACS and anti-CD97 (VIM3b) antibody (Biolegend Cat. #336305) into complete DMEM in a 96-well TC-treated plate. Wells containing the clonal populations were individually passaged and assessed.

### Assessing CD97 expression

CD97 expression was assessed with flow cytometry and quantitative RT-PCR (qPCR). Cells were detached using TrypLE Express enzyme (Gibco) and washed with DPBS. Flow cytometry utilized an anti-CD97 (VIM3b) antibody (Biolegend Cat. #336305) on an Attune NxT cytometer (Thermo Fisher Scientific). RNA for qPCR was isolated with a Qiagen RNeasy Mini kit. cDNA was created using qScript XLT cDNA Supermix (Quanta Bio) following standard manufacturer’s protocol. cDNA was diluted to a constant concentration for all samples to ensure similar nucleic acid loading levels. qPCR was carried out using PerfeCTa SYBR Green Fastmix ROX (Quanta Bio). The qPCR primers for CD97 were GCTCAACAAGAAGGTTCGGG (forward) and ATATGCCGGACTCTGATGCC (reverse), while primers for housekeeping gene GAPDH were CATGACAACTTTGGTATCGTGG (forward) and CCTGCTTCACCACCTTCTTG (reverse). qPCR was performed on an Applied Biosystems QuantStudio 3 following recommended guidelines described by Applied Biosystems for Syber: 95 °C for 10 min, followed by 40 cycles of 95 °C for 15 s and 60 °C for 1 min. Ct values were calculated using the StepOne software accompanying the real-time cycler. Samples were prepared with three technical replicates for each primer pair.

### Western blot

For metabolic pathway Western blots, cultured U251 cells in 10 cm dishes were washed with PBS (Gibco) then placed in serum-free DMEM (Gibco) for 5 h to remove the ability of FBS compounds to overactivate the ERK/MAPK and PI3K/AKT pathways^51^. The cells were then treated with 50 nM Human Recombinant Insulin (Fisher Scientific) for 5 min to trigger the ERK/MAPK and PI3K/AKT pathways in a controlled reproducible manner allowing relative modulation by external stimuli to be reproducibly measured^51^. The media was then aspirated, a rapid PBS wash was performed, then 400 μl of 1× RIPA buffer (Cell Signaling #9806) with protease inhibitor and phosphatase inhibitor was added directly to the plate. The resulting lysate was collected and proteins were extracted in accordance with the RIPA protocol.

Protein concentration was measured with a BCA assay (Thermo Scientific #23225). Western Blot gel was run according to protocol for the Mini-Protean Electrophoresis System (Bio-Rad) and transferred to the membrane according to the Mini Trans-Blot protocol (Bio-Rad). Blocking was performed with 5% milk. Primary antibodies used and their dilutions were GAPDH 1:50,000 (Cell Signaling #5174), MAPK/ERK 1:750 (Cell Signaling #9102), phospho-MAPK/ERK 1:2000 (Cell Signaling #4370), AKT 1:750 (Cell Signaling #4691), and phospho-AKT 1:750 (Cell Signaling #13038). All were incubated in anti-rabbit secondary at 1:8000. Enhanced chemiluminescence imaging was performed using an Odyssey Imaging System (Li-Cor Biosciences) and quantified using Image Studio (Li-Cor Biosciences).

### Cell proliferation assay

U251 cells with CRISPRi knockdown or CRISPR/Cas9 knockout of CD97 and appropriate controls were plated at 1000 cells per well in 96 well plates. Proliferation was continuously assessed using the xCELLigence RTCA MP instrument (ACEA Biosciences) to measure impedance as a surrogate for cell count over 120 h^52^. First, 50 µL of media was added to each well of 96 well E-Plates (ACEA Biosciences) and the background impedance was measured and displayed as Cell Index. Dissociated adherent GBM cells were seeded at 1000 cells/well of the E-Plate in a volume of 100 µL and allowed to passively adhere on the electrode surface. Post seeding, the E-Plate was kept at ambient temperature inside a laminar flow hood for 30 min and then transferred to the RTCA MP instrument inside a cell culture incubator. Data recording was initiated immediately at 15-min intervals for the duration of the experiment.

### Recombinant CD97 expression and purification

The N-terminal extracellular domain of CD97 was cloned into an pFUSE expression vector and expressed as previously described^53^. Briefly, the extracellular domain of CD97 comprising of residues 24–540 was fused to a vector as a secreted protein with a Fc domain and avidin tag fused to the C-terminus. CD97 was then transfected into expi293 mammalian expression system according to manufacturer's protocol (Thermo Fisher Scientific). Of note, an expi293 cell line that constitutively expressed BirA-ER was used to enable robust in situ biotinlyation as the protein is being expressed. After 3–5 days of expression, it was harvested and purified using a protein A column and concentrated to 5 µM in PBS.

### Bio-layer interferometry (BLI)

BLI experiments were performed using an an Octet RED384 System (Satrious AG). Biotinylated recombinant CD97 was first immobilized onto streptavidin biosensors, followed by association and dissociation detection with 6.25 nM to 100 nM human DAF-Fc, a monoclonal anti-CD97 antibody that binds the same region of CD97 as DAF kindly provided by Kathleen Kelly of the NIH, or monoclonal antibody Ab108368 from Abcam with known Kd value. All assays were performed in PBS with 0.1% BSA kinetic buffer. Affinity (Kd) of each were calculated from a global fit (1:1) of the data using the Octet RED384 software.

### DAF-Fc treatment of cultured cells to assess effects on protein phosphorylation

U251 cells in culture with DMEM containing 10% FBS as described above were treated with 1 mg/ml human DAF-Fc or ACE2-Fc fusion proteins for 19 h. The cells were then washed and placed in serum-free DMEM where they continued to receive 1 µg/ml human DAF-Fc or ACE2-Fc for 5 h, totaling 24 h of drug treatment. This was followed by insulin treatment and protein collection as described above.

### NanoString

Cells cultured in a 10 cm plates were harvested and RNA was isolated using a Qiagen RNeasy Mini plus kit. NanoString multiplex transcriptomic analysis was carried out per the manufacturer’s protocol. Hybridization of probes was carried out for 17 h and nCounter Hs_Tumor Signaling Panel was used for the reaction. Raw data was exported using the Nsolver4.0 software followed by differential gene expression analysis using the DeSeq2 package in R. KEGG pathways analysis was carried out using the EnrichR software.

### Metabolomics

U251 control (U251-dCas9-KRAB-MeCP2) and U251 CD97 knockdown (U251-dCAS9-KRAB-MeCP2-CD97sgRNA) cells were incubated with 0.1 g/L ^13^C_6_-glucose for 24 h, after which cells were rinsed with cold 150 mM NH_4_AcO, pH 7.3, scraped off in 1 ml cold 80% MeOH, incubated with 5 nmol norvaline, vortexed three times on ice, spun down with supernatant resuspended in 200 µl 80% MeOH, spun down again, and re-suspended in protein extraction buffer with protein content measured by Bradford assay. Metabolites were then dried down in EZ‐2Elite evaporator at 30 °C using program 3 (aqueous). Dried metabolites were resuspended in 50% ACN and 5 μl loaded onto a Luna 3 µm NH2 100A (150 × 2.0 mm) column (Phenomenex). The chromatographic separation was performed on an UltiMate 3000 RSLC (Thermo) with mobile phases A (5 mM NH_4_AcO pH 9.9) and B (ACN) and a flow rate of 200 μl/min. The gradient from 15% A to 95% A over 18 min was followed by 9 min isocratic flow at 95% A and reequilibration to 15% A. Metabolite detection was achieved with a Thermo Q Exactive mass spectrometer run with polarity switching (+ 3.0 kV/ − 2.25 kV) in full scan mode with an *m/z* range of 65–975. TraceFinder 4.1 (Thermo) was used to quantify metabolites by area under the curve using retention time and accurate mass measurements (< 3 ppm). Relative amounts of metabolites were calculated by summing up all isotopologues of a given metabolite and normalized to cell number. Data analysis, including principal component analysis and hierarchical clustering was performed using in-house scripts in the R programming language.

### Monocyte differentiation assay

THP-1 cells were cultured in THP-1 media consisting of Roswell Park Memorial Institute (RPMI) 1640 medium with 4 g/L glucose, 0.584 g/L L-glutamine, 0.11 g/L sodium pyruvate, 3.7 g/L NaHCO_3_, 10% FBS, 1% penicillin/streptomycin, 1% HEPES buffer, and 0.1% 2-mercaptoethanol. THP-1 cells were collected and dyed with CellTracker Green dye (Thermo Fisher Scientific). For co-culture 150,000 U251 tumor cells and 450,000 THP-1 were added to non-TC-Treated T75 flasks in 15 ml of THP-1 media. Positive differentiation control was achieved with 50 ng/ml Phorbol 12-myristate 13-acetate (PMA). All conditions were plated in triplicate and collected at 96 h. Media was collected then flasks were treated with Accutase Cell Detachment Solution (Innovative Cell Technologies) to retrieve remaining cells. The media and Accutase from each sample were then combined and cells washed in DPBS. Monocyte differentiation was assessed by flow cytometry with an anti-CD11b (M1/70) antibody (Biolegend Cat. #101261) on an Attune NxT cytometer.

### Statistical analysis

For comparing continuous variables, t-test (parametric) or Kruskal Wallis/Wilcoxon rank sum test (non-parametric) were used, with analysis on SPSS (IBM, v24.0). Multiple comparisons involving multiple groups compared for a single variable were analyzed by 1-way ANOVA and Tukey’s test for multiple testing of nonparametric data (GraphPad Prism 9). For multiple comparisons involving multiple groups compared for multiple variables, FDR-adjusted *q* values were calculated. Experiments were done with three technical and three biologic replicates. Error bars are standard deviations amongst biologic replicates. The threshold for statistical significance was *P* < 0.05 or FDR-adjusted *q* < 0.05.

## Supplementary Information

Supplementary Information.
