# Supplementary material for: CD97 is associated with mitogenic pathway activation, metabolic reprogramming, and immune microenvironment changes in glioblastoma
Source: Sci Rep. 2022 Jan 27;12:1464. doi: 10.1038/s41598-022-05259-y (PMC8795421; doi:10.1038/s41598-022-05259-y)
Supplement: Supplementary file 1 — Supplementary Information. [file 41598_2022_5259_MOESM1_ESM.pdf]

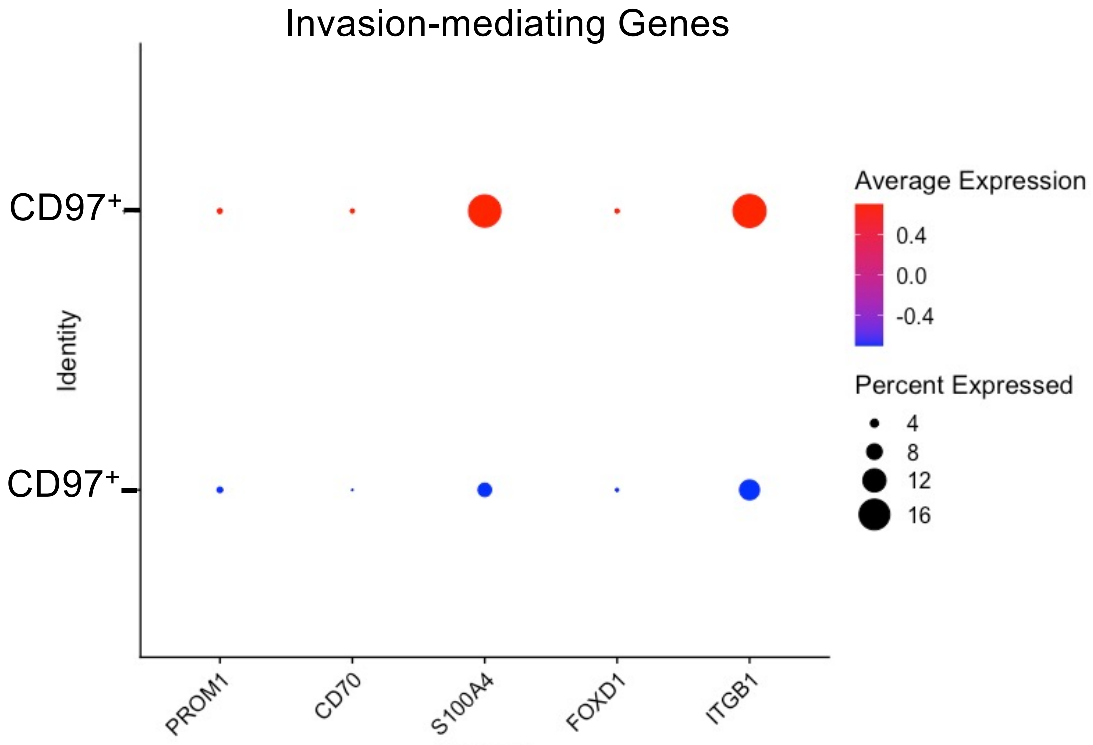

Supplementary Figure S1. Related to Figure 2. Invasion-mediated genes are more expressed in CD97$^+$ GBM cells than in CD97- GBM cells. Compared to individual CD97$^-$ GBM cells, individual CD97$^+$ GBM cells expressed higher levels of 5 genes identified from a previous study that performed gene expression profiling of invasive GBM cells in patients.

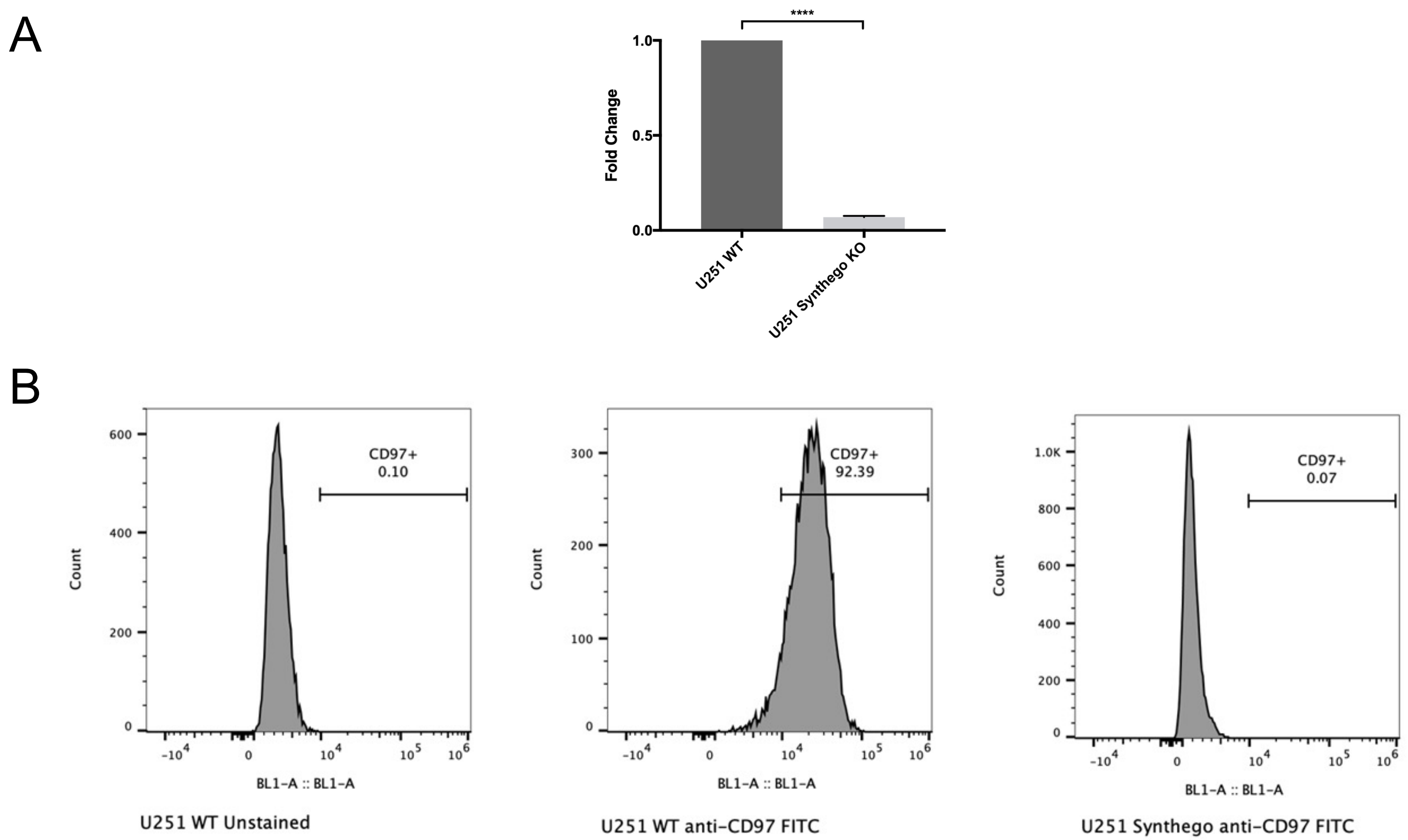

Supplementary Figure S2. Related to Figure 2F, 3E-F and Supplementary Figure S4. Validation of CD97 knockout in U251 cells. CD97 knockout in U251 cells after delivery of Cas9 and gRNAs targeting CD97 was validated using (**A**) qPCR with CD97 normalized to GAPDH for each sample and (**B**) flow cytometry. *P<0.05; **P<0.01; ***P<0.001.

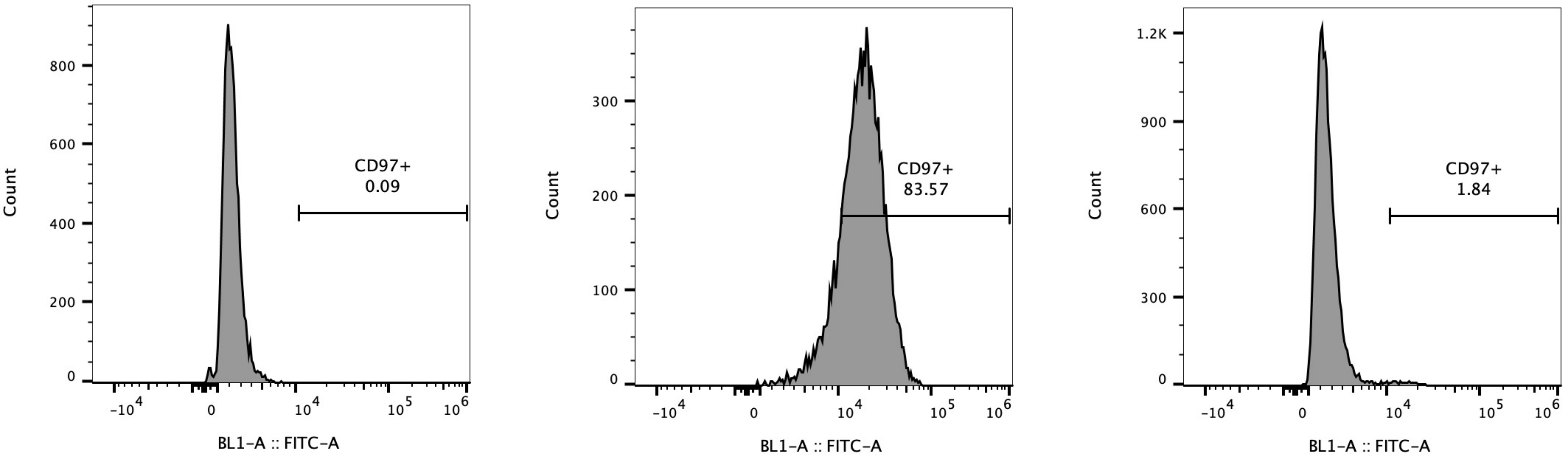

U251 WT Unstained

dCAS9-KRAB-MeCP2- Anti-CD97-FITC

dCAS9-KRAB-MeCP2-CD97sgRNA Anti-CD97-FITC

Supplementary Figure S3. Related to Supplementary Figures S4 and S11. Flow cytometry to confirm CD97 knockdown in U251 cells via use of CRISPRi. Flow cytometry analysis showing efficient knockdown of CD97 using sgRNA targeting CD97 in U251 cells stably expressing dCAS9-Krab-MeCP2.

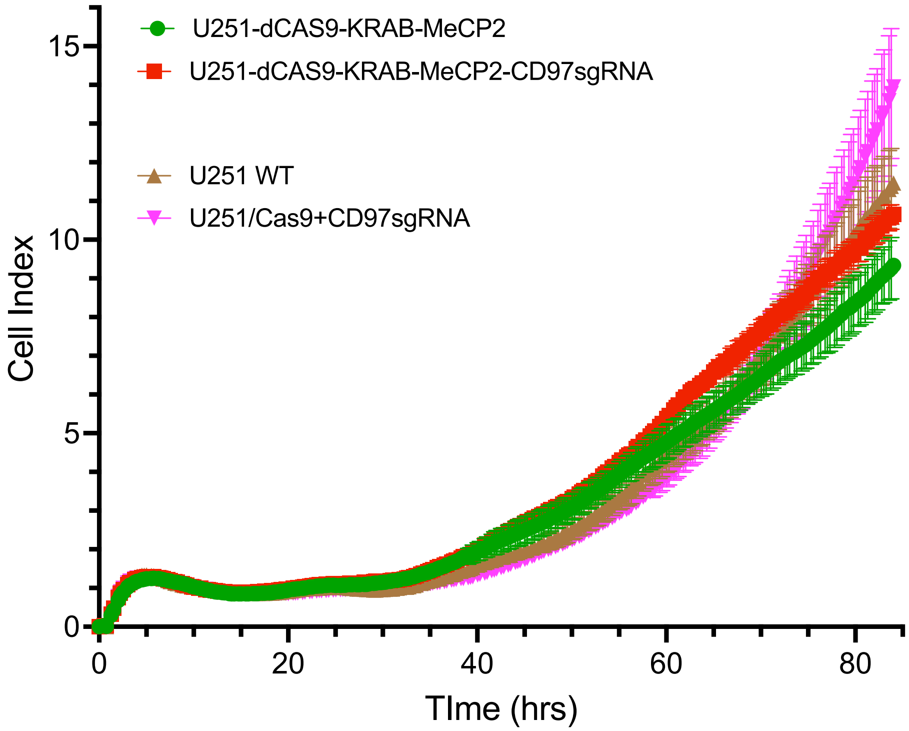

Supplementary Figure S4. CD97 knockdown or knockout does not alter GBM cell line proliferation. Related to Figure 4C. Proliferation of 2500 cells/well in a 96-well plate was continuously assessed using the xCELLigence RTCA MP instrument to measure impedance as a surrogate for cell count over 84 hours. U251-dCas9-KRAB-MeCP2-CD97sgRNA and U51-dCas9-KRAB-MeCP2 had the same proliferation (P=0.6-0.8), as did U251 WT (wild-type) and U251/Cas9-CD97sgRNA (P=0.6-0.9).

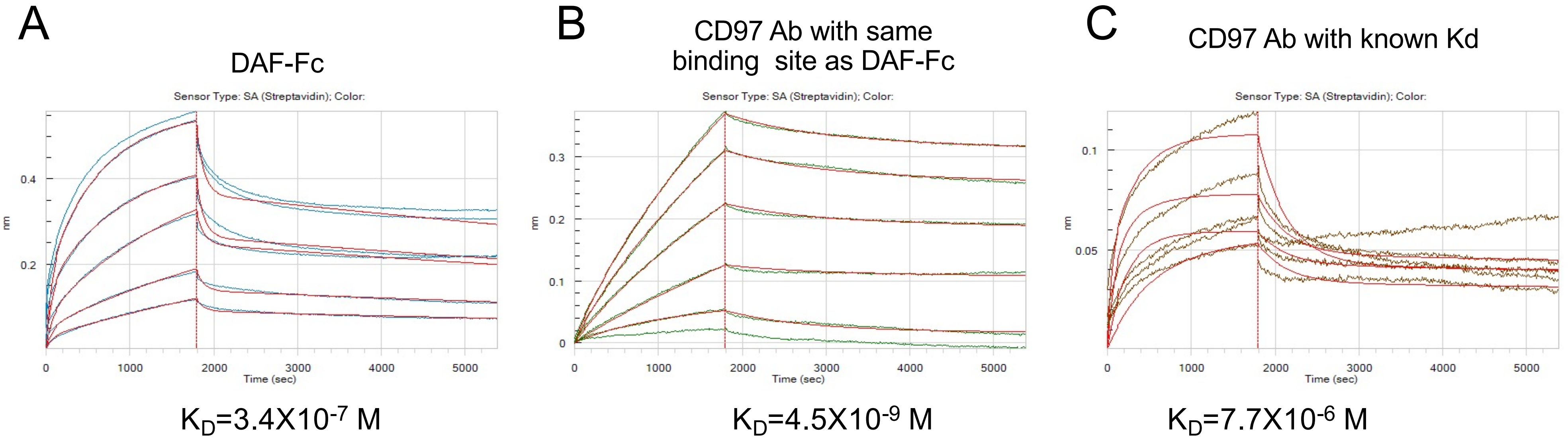

Supplementary Figure S5. Related to Supplementary Figure S6. A DAF-Fc fusion protein has affinity for CD97 comparable to that of a monoclonal antibody. Affinities for recombinant human CD97 of (**A**) DAF-Fc (a fusion protein combining human DAF with the human Fc region), (**B**) a monoclonal anti-human CD97 antibody binding the same site on CD97 as DAF-Fc, and (**C**) a monoclonal anti-human CD97 antibody with known Kd for CD97 were compared using the Octet red machine. During the first 1800 seconds (30 minutes), binding occurred, with detachment measured over the ensuing hour. Kd value for the antibody with known Kd matched the published Kd value, validating the technique. Solid lines show response curves for twofold dilution titration spanning 6.25 nM to 100 nM. Red lines show calculated fits. Bio-Layer Interferometry (BLI) measurements show robust binding of human DAF-Fc (**A**) to extracellular domain of CD97. The binding affinity of DAF-Fc (K_D = 3.4 X 10^{-7}) was lower than an antibody binding the same site as DAF-Fc (**B**) (K_D = 4.5 X 10^{-9}), but superior to a commercial antibody binding another site on CD97 (**C**) (K_D = 7.67 X 10^{-6}).

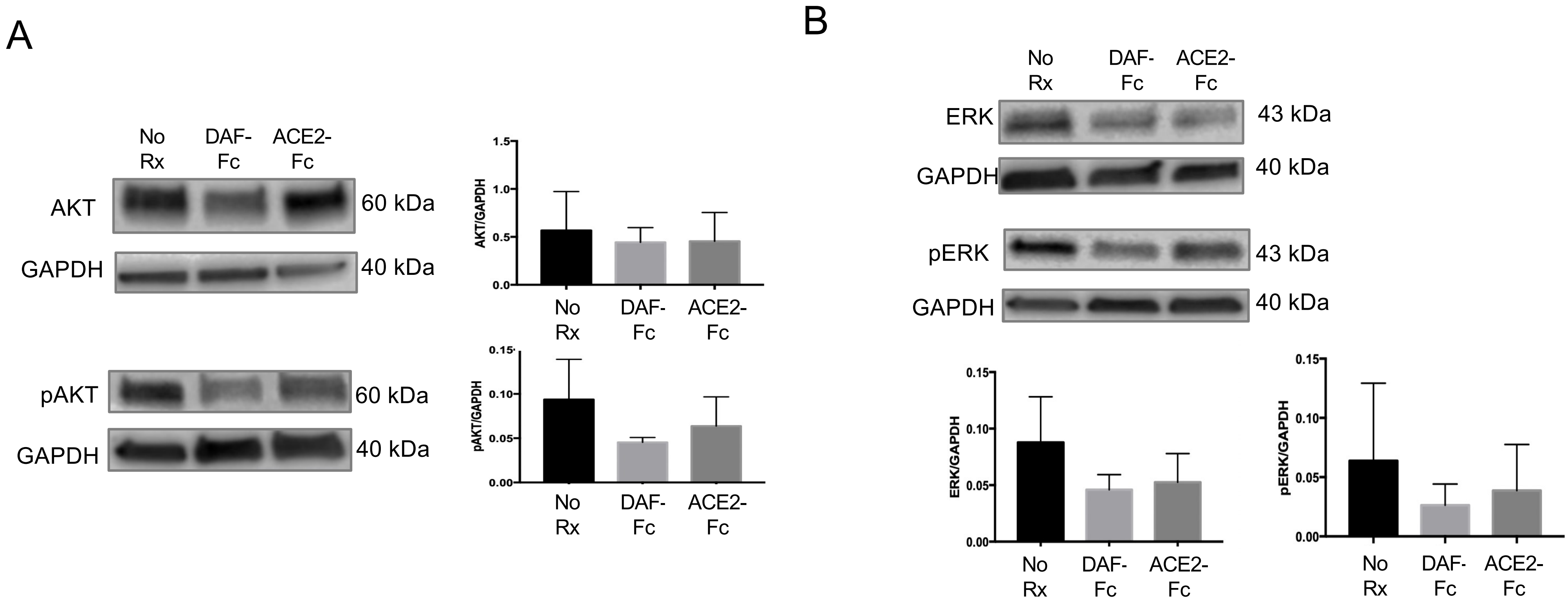

Supplementary Figure S6. DAF-Fc treatment of GBM cells. Related to Supplementary Figure S5. 150,000 U251 cells were allowed to adhere overnight and then treated with 1 μg/mL human DAF-Fc or human ACE2-Fc fusion protein (which doesn't bind U251) for 24 hours, after which they underwent western blot for (**A**) Akt and phosphorylated Akt and (**B**) ERK and phosphorylated ERK. No difference in levels of any of the proteins were seen when band intensities were quantified and normalized to GAPDH. Study was performed with biological triplicates with a representative blot shown here. Fulll-length blots from triplicate studies are shown in **Supplementary Figures S14-S15**.

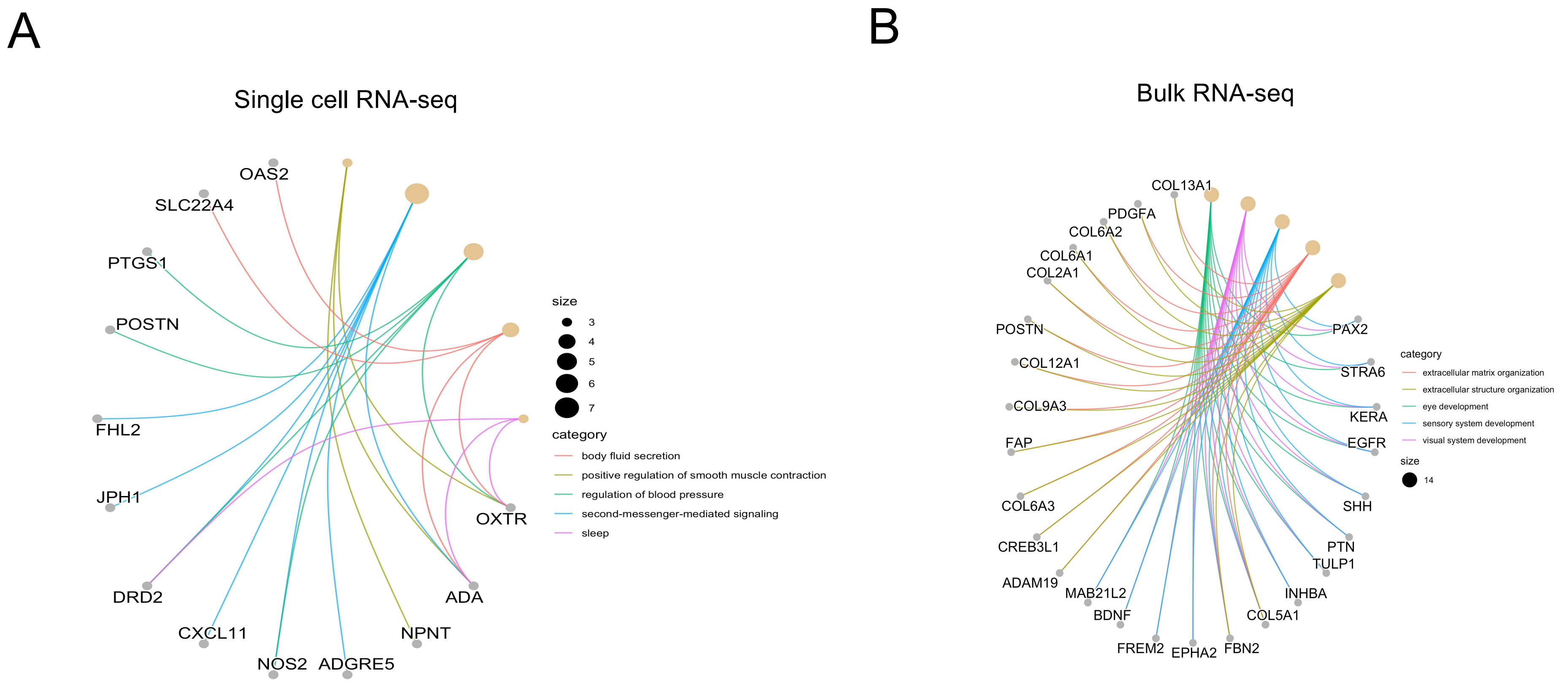

Supplementary Figure S7. Network analysis from RNA seq. Related to Figures 3A-B. Network analysis was performed on the (A) top 25 genes expressed in CD97+ single cells vs CD97- single cells and (B) top 133 genes expressed in CD97hi vs. CD97lo GBMs from bulk RNA-seq. Genes with the most pathways in common are placed onto a network with the categories of these pathway demarcated.

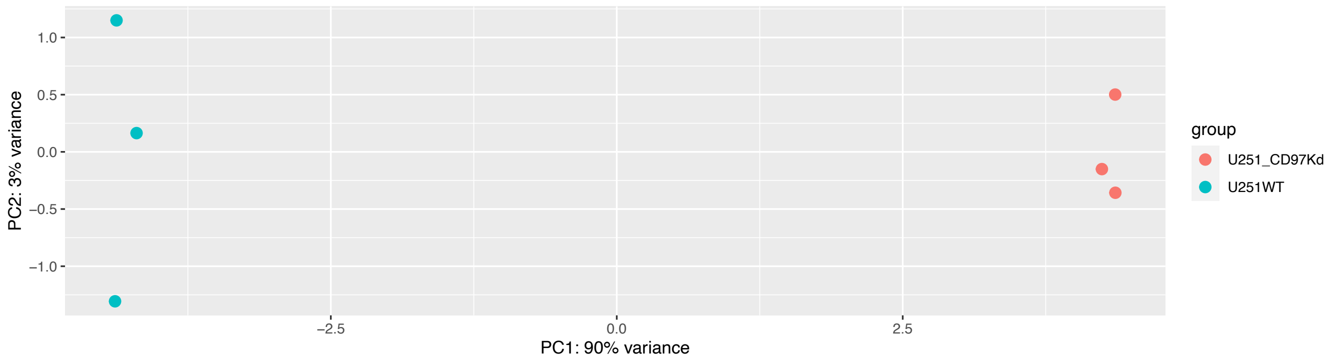

Supplementary Figure S8. Related to Figures 3E-F. Principal component analysis showing the variance between GBM cells with and without CD97 knockout. Shown is the PCA plot for U51 wild type (U251WT-Blue) and CD97 knockout U251 cells (U251_CD93kd-Red) analyzed in the Nanostring multiplex transcriptomic aasay. PC1 showed 90% variance between the two cell types.

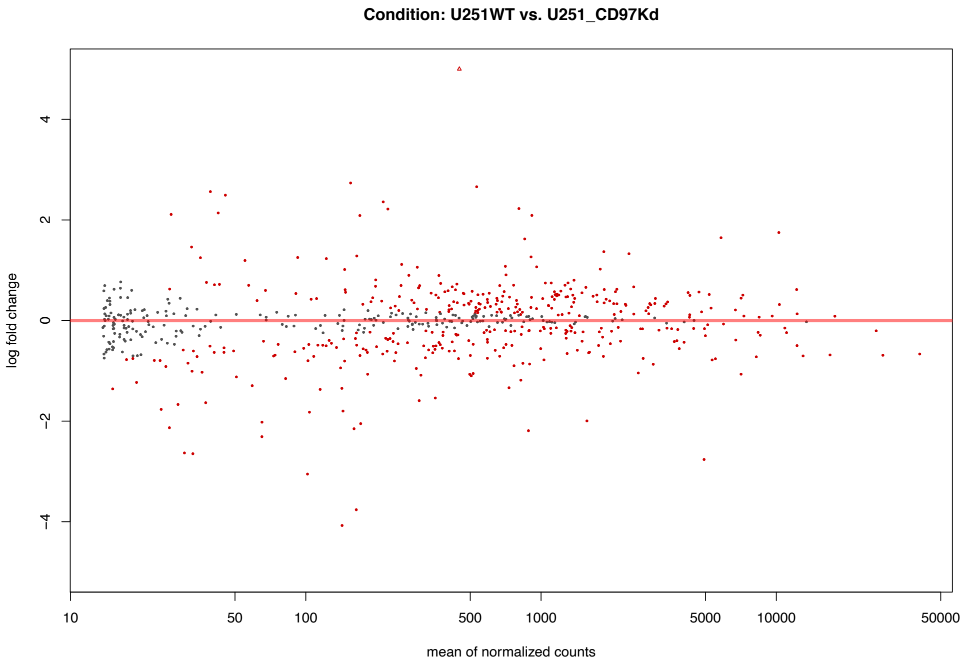

Supplementary Figure S9. Related to Figures 3E-F. MA plots showing the overall fold changes with respect to normalized raw counts of genes in the nCounter Nanostring tumor Signaling panel. Raw mean counts generated from Nanostring nSolver was used as input to perform the DeSeq2 analysis in R which revealed the fold change patterns with respect to mean of normalized counts.

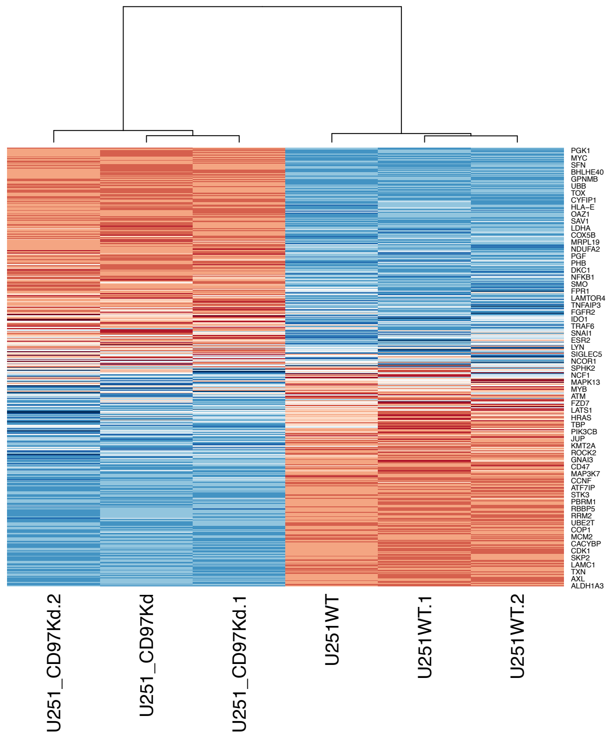

Supplementary Figure S10. Related to Figures 3E-F. Heatmap showing most significant differentially expressed genes between U251WT and U251_CD97Kd cells in the nCounter Nanostring Tumor Signaling panel. DeSeq2 analysis was carried out in R to identify the differentially expressed genes. Heat map was created using R.

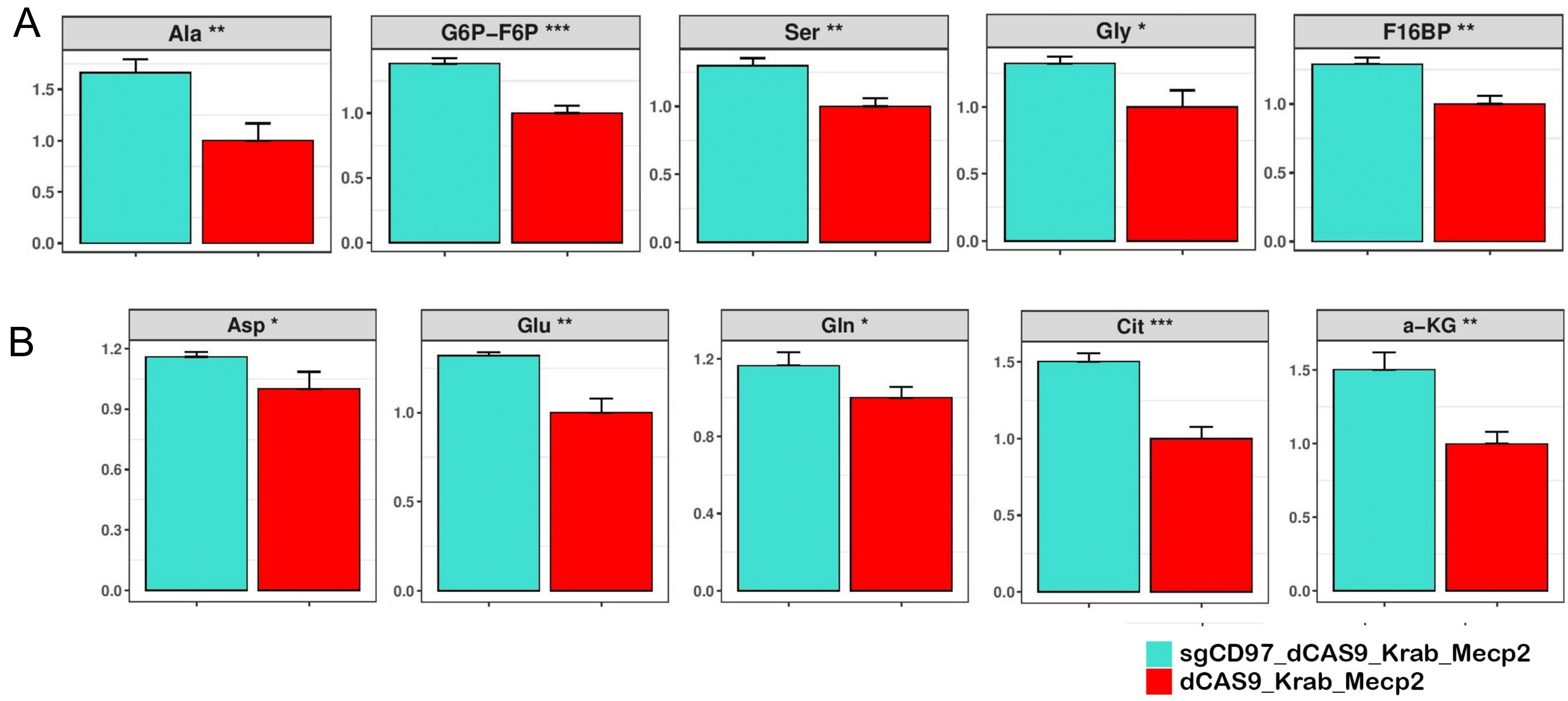

Supplementary Figure S11. Metabolomic analysis of GBM cells with and without CD97 knockdown. Metabolomic assessment revealed (**A**) reduced levels of glycolytic metabolites alanine (Ala), glucose-6-P (G6P), fructose-6-P (F6P), serine (Ser), glycine (Gly), and fructose-1,6-bisphosphate (F16BP) and (**B**) reduced levels of TCA metabolites aspartate (Asp), glutamate (Glu), glutamine (Gln), citrate (Cit), and a-ketoglutarate (a-KG) in U251 control cells (red) relative to U251 CD97 knockdown (cyan) cells. *P<0.05; **P<0.01; ***P<0.001.

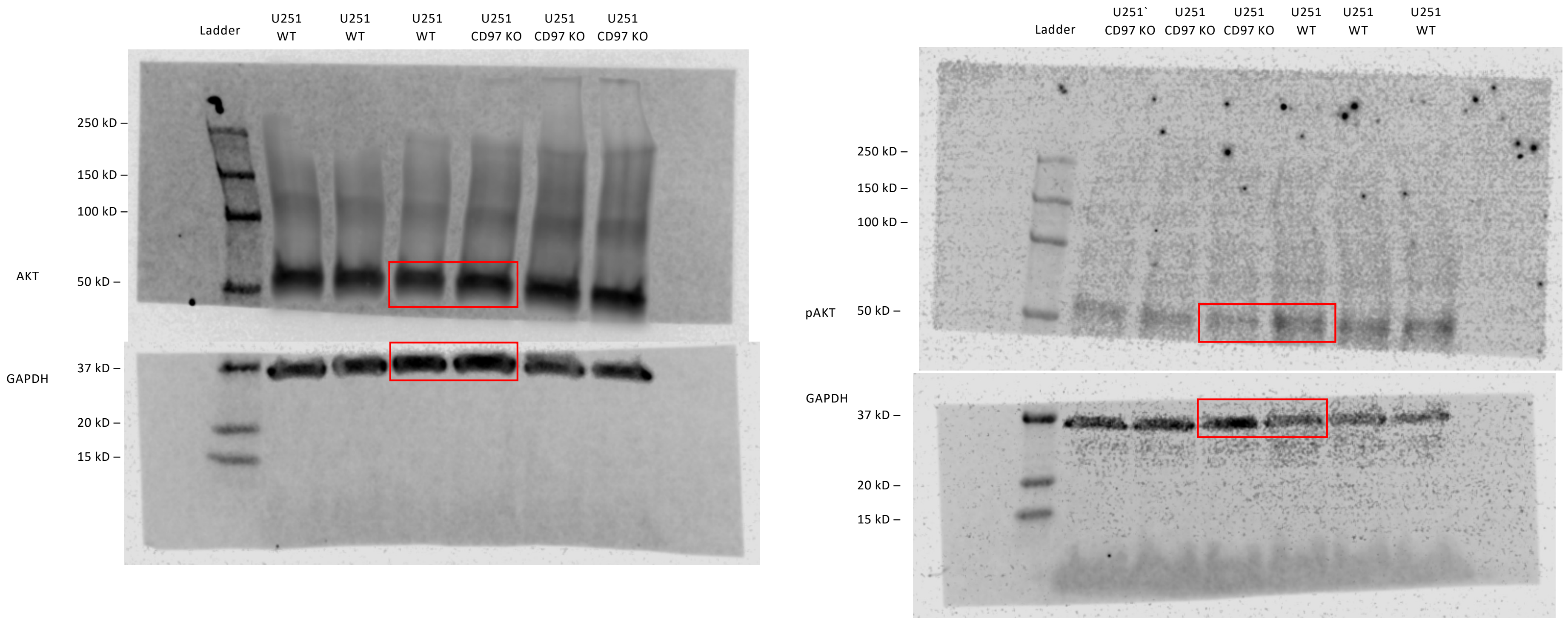

Supplementary Figure S12. Full-length western blots for Akt and phosphorylated Akt from Figure 2F. Shown are full-length Western blots for Akt (left) and phosphorylated Akt (right) in U251 wild-type cells (U251 WT) and U251 cells with CD97 knockout (U251 CD97 KO) with each cell-type shown in triplicate. GAPDH blots are shown below (lower halves of gels were cut to blot for GAPDH). Areas boxed in red were used in **Figure 2F**, with the blot on the right flipped for **Figure 2F** to put U251 WT on the left and U251 CD97 KO on the right the way they are arranged in the blot on the left.

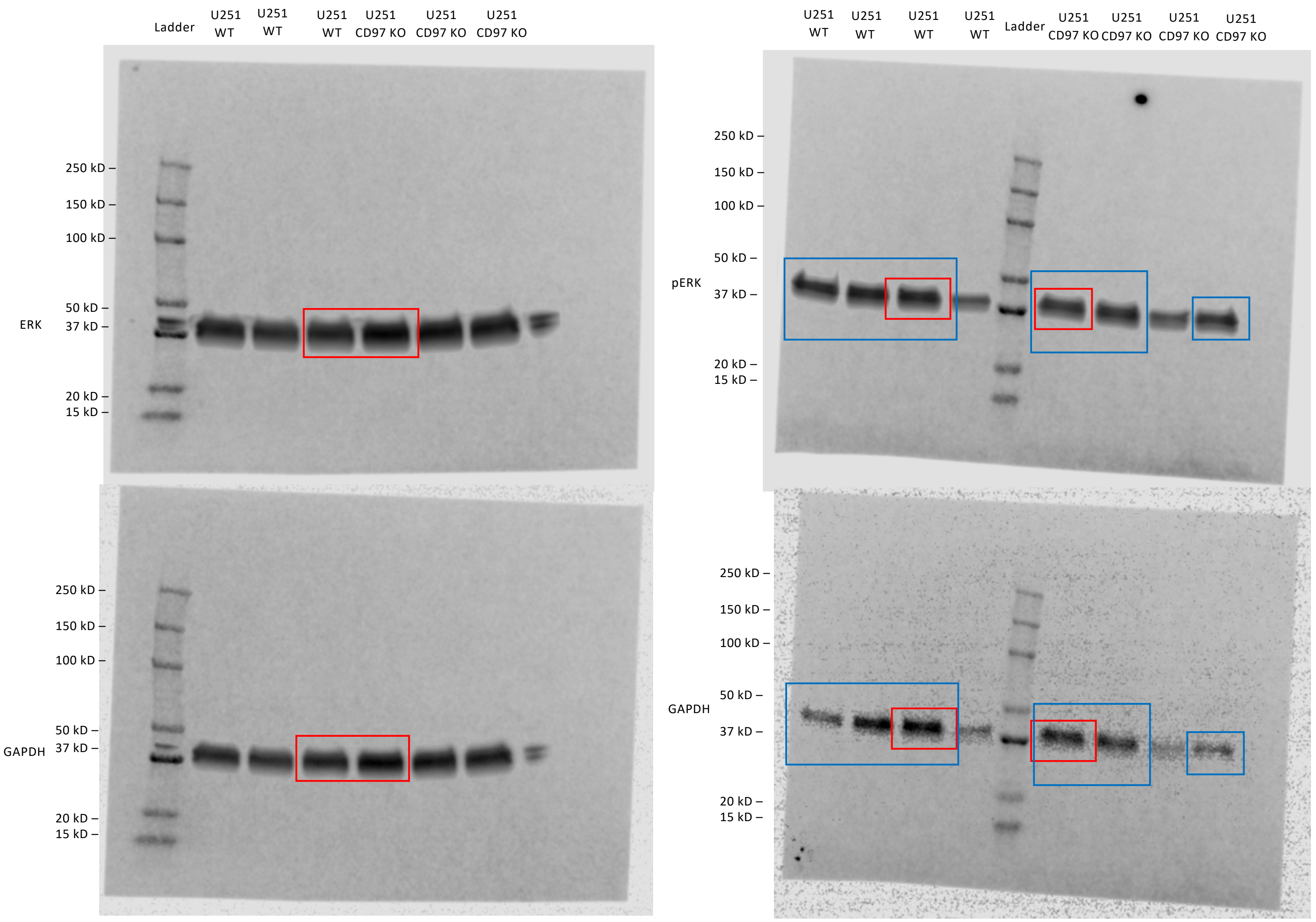

Supplementary Figure S13. Full-length western blots for Erk and phosphorylated Erk from Figure 2F. Shown are full-length Western blots for Erk (left) and phosphorylated Erk (right) in U251 wild-type cells (U251 WT) and U251 cells with CD97 knockout (U251 CD97 KO) with each cell-type in triplicate and GAPDH blots below. Wells boxed in red were used in **Figure 2F**. The rightmost spot on the left blot contains protein spillover, while on the right blot one well of each cell type didn't load well and was excluded from analysis with wells in blue boxes used for quantification.

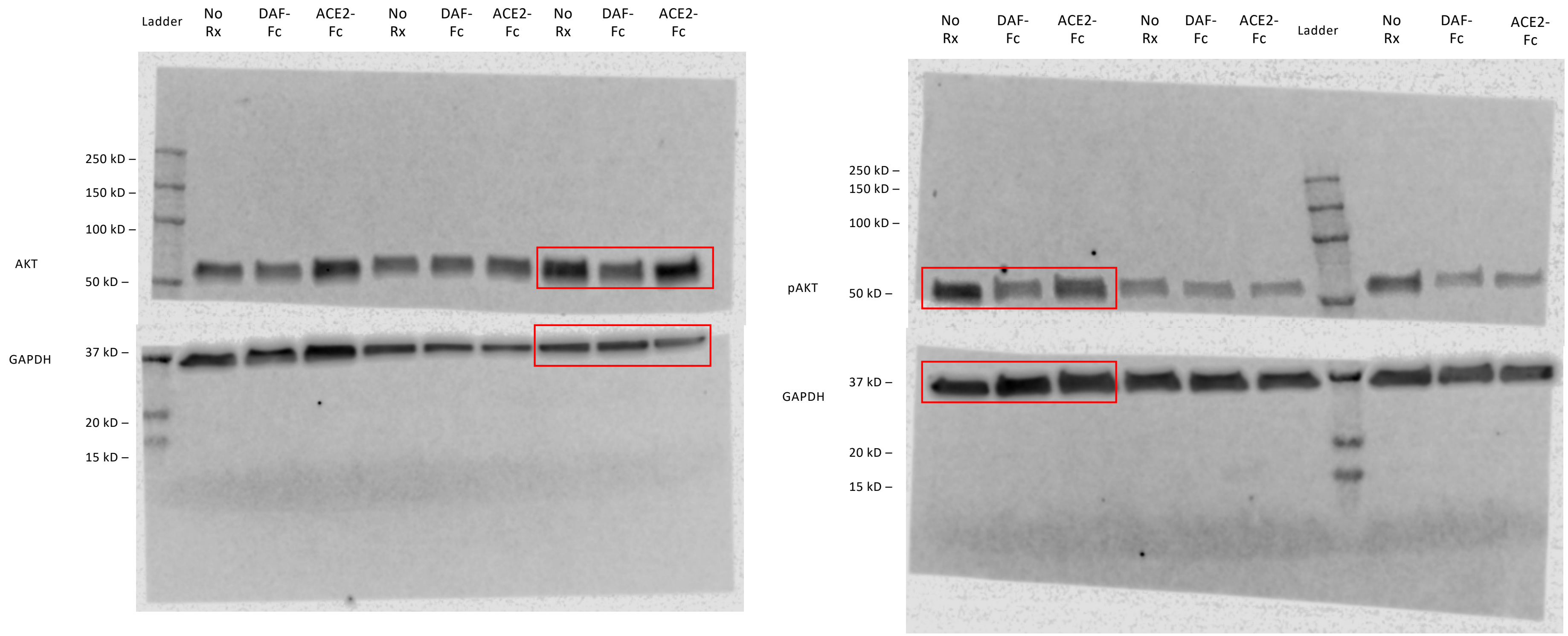

Supplementary Figure S14. Full-length western blots for Akt and phosphorylated Akt from Supplementary Figure S6A. Shown are full-length western blots for Akt (left) and phosphorylated Akt (right) with the GAPDH bands shown below (lower halves of gels were cut to blot for GAPDH) for triplicate U251 GBM cells treated with vehicle alone ("No Rx"), human DAF-Fc ("DAF-Fc"), and a fusion protein between ACE2 and Fc that does not bind U251. Areas boxed in red were used in **Supplementary Figure S6A**.

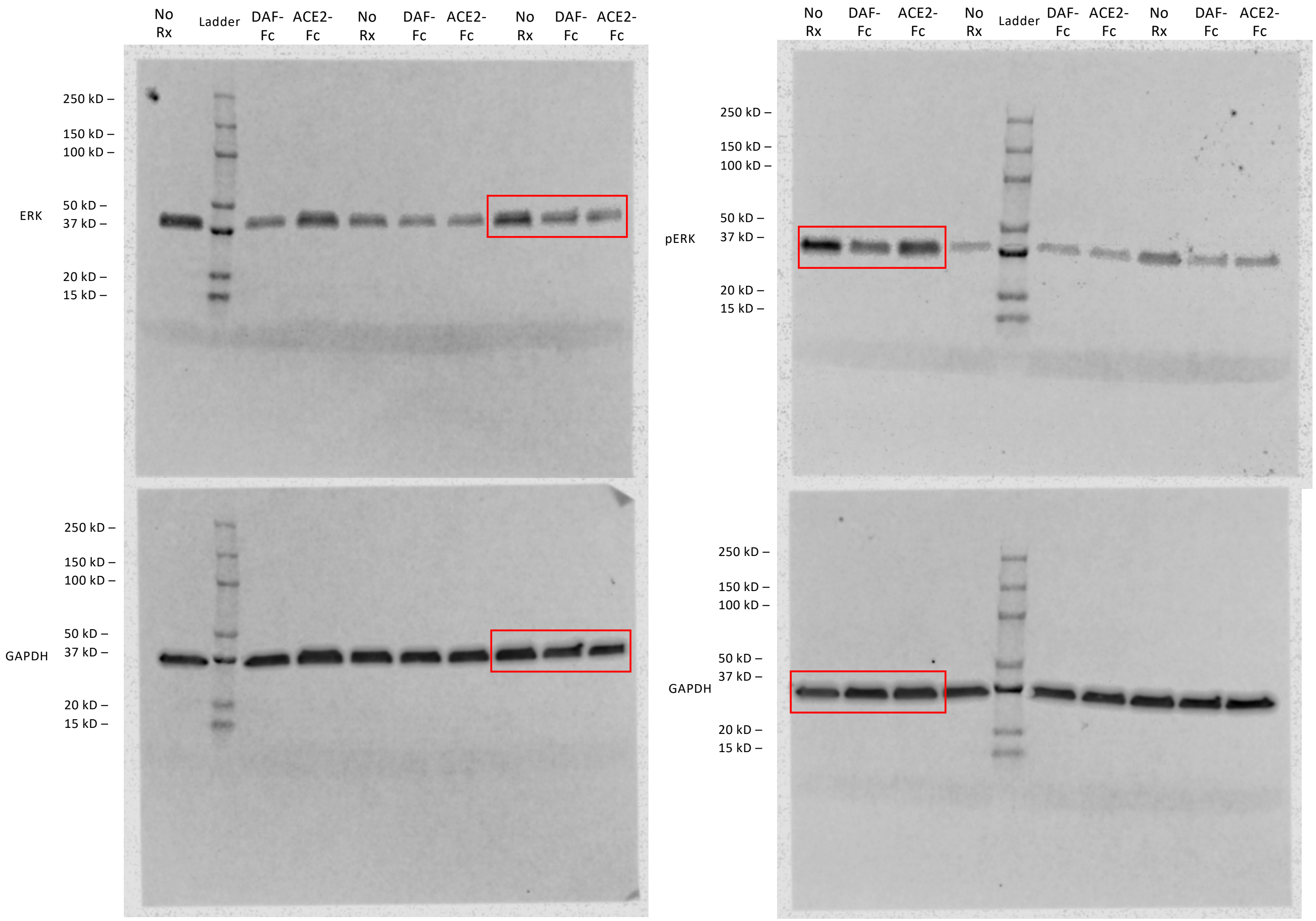

Supplementary Figure S15. Full-length western blots for Erk and phosphorylated Erk from Supplementary Figure S6B. Shown are full-length western blots for Erk (left) and phosphorylated Erk (right) with the GAPDH bands shown below for triplicate U251 GBM cells treated with vehicle alone (“No Rx”), human DAF-Fc (“DAF-Fc”), and a fusion protein between ACE2 and Fc that does not bind U251. Areas boxed in red were used in **Supplementary Figure S6B**.

Supplementary Table S1. Most differentially expressed genes in CD97+ GBM cells and CD97- GBM cells. Shown are the top 100 genes with greatest variable expression (from high to low) in CD97+ GBM cells and CD97- GBM cells.

CD97+ GBM cells	CD97- GBM cells
KCNH7	CCL4
MT-CYB	HLA-DRA
BMPER	NMB
PCDH11Y	CRYAB
RYR3	FABP7
DGKB	S100A4
GLIS3	METRN
MT-CO2	HMGB2
CXCL8	XACT
VIM	HLA-DPA1
DCC	CD74
SOX6	LINC01266
MT-CO3	CCL3
TSHZ2	IL1B
CCL2	CXCL8
VEGFA	FCGBP
MT-ATP6	ARL6IP1
RPL26	HSPA6
TMEM108	MEG3
MT-ND4	CCL3L3
AC124254.1	ARC
RPS27	NRG1
LINC01266	NLRP3
TMSB4X	EFNA5
MT-ND1	G0S2
MT-ND3	IGFBP2
GRIN2B	IL1B
RPS15A	NR4A3
PCDH15	HLA-DRB1
RPL12	ID3
TNS3	MET
AC073050.1	AC068992.1
RPL13A	CCL4L2
OPCML	GLRA2
RPL34	SPP1
G0S2	ZMAT4
CHRM3	HPSE2
PCDH11X	C1QB
RPL23A	C1QC
NRXN1	SERPINE1
MT-CO1	TUBA1B

RPL28	HIST1H4C
RBFOX1	C1QA
KALRN	PLCG2
SNTG1	PTTG1
RPL39	CGNL1
RPL3	CCL4L2
TNR	APOC1
RPS14	SPP1
KCNIP4	IL1A
GNA14	CALM2
RPL41	KCNE4
SORCS3	HLA-DPB1
RPS18	IER3
RPLP1	H2AFZ
IGFBP5	PTCHD4
EYA2	PRDX2
DCLK1	TSHZ2
RPLP2	PLAUR
ITPR1	CXCL8
PLXNA4	MROH8
ADAMTS9	CENPF
DACH1	TOP2A
FTH1	HLA-DRB5
RPL11	CTSD
RPS25	FXYD6
ACAN	CH25H
RPL21	RNF219-AS1
PRICKLE1	LGALS1
TCF7L1	PFN1
DNAH11	BIRC5
CCL20	ACAN
RPS12	F13A1
DGCR5	MND1
PTCHD1-AS	RGS6
LINC01239	MT1X
RPL30	KCNIP4
HIST1H4C	LINC00499
SEMA5B	UBE2S
RPS29	RNF144B
ZMAT4	TIMP1
SLC11A1	CDH4
CRYAB	CCL20
GABRB1	UNC5D
CDH4	PRICKLE1
IFNG-AS1	VIM

DACH2	GRIK1
RPL36A	RIT2
TRPM3	PFN2
LAMA2	S100A11
TPT1	MIR4435-2HG
LHFPL3	PLEK
APOE	CPNE4
MT-ND2	UQCR11
MIR4435-2HG	ENSG00000260411
HSPA6	FTL
PTPRN2	MIF
SERPINE1	PTGER3
PCSK5	RGS1
IFI27	RBP1

Supplementary Table S2. Top 250 upregulated and top 250 downregulated genes in U251 wild type cells relative to U251 cells with CD97 knockout

	baseMean	log2FoldChar	lfcSE	stat	pvalue	padj
SFN	142.61923	-4.0746804	0.23459593	-17.368931	1.42E-67	2.53E-66
CA9	163.892344	-3.7615101	0.20442313	-18.400609	1.30E-75	2.46E-74
ITGB3	101.673726	-3.0527641	0.22628707	-13.49067	1.77E-41	1.61E-40
CCL2	4931.10724	-2.7626197	0.03403473	-81.170605	0	0
CSF2RA	33.1022363	-2.6480257	0.37397778	-7.0807034	1.43E-12	5.42E-12
SIRPA	30.4839316	-2.6328515	0.37913987	-6.9442749	3.80E-12	1.36E-11
PSMB9	65.0587609	-2.3092709	0.24557819	-9.403404	5.28E-21	2.82E-20
COL6A1	883.954574	-2.1907719	0.06584697	-33.270657	1.03E-242	6.40E-241
GPNMB	160.325057	-2.1514614	0.16162037	-13.311821	1.98E-40	1.74E-39
FGFBP1	26.3164287	-2.1314535	0.37008059	-5.7594307	8.44E-09	2.59E-08
IGFBP3	171.101091	-2.0499906	0.14573972	-14.066108	6.14E-45	6.28E-44
TGFB3	65.1129412	-2.020466	0.23627245	-8.5514242	1.22E-17	5.79E-17
LTBP1	1566.55825	-1.9960029	0.05189537	-38.462061	0	0
COL6A3	103.629754	-1.8217734	0.18171713	-10.025326	1.18E-23	7.01E-23
CXXC5	143.904323	-1.7999157	0.15687669	-11.473443	1.79E-30	1.27E-29
CEACAM3	24.2789366	-1.7662501	0.39453024	-4.4768433	7.58E-06	1.85E-05
LAIR1	28.6281384	-1.6687761	0.33984468	-4.9104084	9.09E-07	2.36E-06
CXCR4	37.5374302	-1.6341729	0.30177859	-5.4151385	6.12E-08	1.75E-07
HMOX1	303.379714	-1.592808	0.10921847	-14.583687	3.57E-48	4.20E-47
VEGFA	355.213452	-1.540415	0.10039289	-15.343866	3.89E-53	5.06E-52
NFKB2	115.085593	-1.3693562	0.16523734	-8.2872077	1.16E-16	5.28E-16
FPR1	15.1041121	-1.3593615	0.45579788	-2.9823778	0.00286019	0.0054248
TAP1	142.33377	-1.3481126	0.14782568	-9.11961	7.54E-20	3.82E-19
TGFB1	730.765364	-1.33713	0.06710619	-19.925584	2.44E-88	5.25E-87
SMAD7	59.1745972	-1.2956199	0.22769411	-5.6901777	1.27E-08	3.84E-08
FAS	19.0792592	-1.2304803	0.39503504	-3.1148637	0.0018403	0.00359983
JUN	820.396598	-1.1853262	0.06223176	-19.046966	6.96E-81	1.45E-79
COL6A2	82.0748802	-1.1540222	0.19429311	-5.9395942	2.86E-09	9.09E-09
HLA-C	50.6981749	-1.1212644	0.24845518	-4.5129442	6.39E-06	1.58E-05
ITGA5	505.069571	-1.0995905	0.07875299	-13.962525	2.64E-44	2.61E-43
WNT5A	308.543338	-1.0866228	0.10166145	-10.688643	1.15E-26	7.56E-26
PRKCA	497.519452	-1.0687289	0.08050828	-13.274771	3.24E-40	2.81E-39
FOS	183.186323	-1.0672711	0.12710049	-8.3970655	4.58E-17	2.12E-16
FSTL1	7082.07601	-1.0663418	0.02778251	-38.381769	0	0
FOXP1	514.57239	-1.0532266	0.08132901	-12.950196	2.34E-38	1.92E-37
MYC	2589.98973	-1.0428314	0.03677427	-28.357641	6.74E-177	2.34E-175
LAMC2	36.2282305	-1.027848	0.2844186	-3.6138564	0.00030168	0.0006582
TLR3	32.8380933	-1.0072967	0.29983981	-3.3594495	0.00078098	0.0015874
MAP3K14	294.979941	-0.953258	0.09968406	-9.5627926	1.15E-21	6.44E-21
COL4A1	140.545042	-0.9419538	0.14350633	-6.5638481	5.24E-11	1.78E-10
OAS1	25.4157101	-0.9142689	0.34692543	-2.635347	0.00840513	0.01498515
BHLHE40	767.524617	-0.899307	0.062978	-14.279702	2.93E-46	3.15E-45
B4GALT1	2998.34516	-0.8691139	0.03455529	-25.151396	1.36E-139	4.05E-138
SORD	893.941863	-0.8651621	0.06300314	-13.732049	6.53E-43	6.17E-42
LOX	837.011771	-0.8501562	0.06101412	-13.933763	3.95E-44	3.85E-43
TNFRSF9	31.5581945	-0.8474348	0.30256589	-2.8008271	0.00509718	0.00949445
REL	146.217706	-0.8054789	0.15243149	-5.284203	1.26E-07	3.52E-07
LTBR	238.121757	-0.8040281	0.11315318	-7.1056609	1.20E-12	4.56E-12
HLA-B	102.134456	-0.7995886	0.17344409	-4.6100655	4.03E-06	1.01E-05
FGFR3	24.0242164	-0.7955486	0.35585379	-2.2356052	0.02537765	0.0422284
TERT	22.702406	-0.7930889	0.37877518	-2.0938248	0.03627559	0.05925645
P4HB	5335.4938	-0.783626	0.02677943	-29.26224	3.14E-188	1.40E-186
FANCI	1028.89685	-0.7828406	0.05470348	-14.310619	1.88E-46	2.09E-45
CCL5	17.2944995	-0.7774171	0.41776698	-1.8608868	0.06276016	0.0986457
VCP	5511.49888	-0.7607028	0.02675863	-28.42832	9.03E-178	3.32E-176
P4HA2	445.681892	-0.757799	0.08051938	-9.4113861	4.90E-21	2.63E-20
NFATC2	18.4022467	-0.7551714	0.39575837	-1.9081627	0.05637019	0.09042415
TJP1	2717.02948	-0.7528337	0.0374899	-20.080971	1.08E-89	2.41E-88
TOX	591.426559	-0.7501285	0.0729072	-10.288812	7.91E-25	4.94E-24
CD28	13.8993947	-0.746569	0.47037438	-1.5871803	0.11247181	0.17034566
SOS1	322.413305	-0.740692	0.10494591	-7.0578459	1.69E-12	6.28E-12
B2M	8222.17778	-0.7218615	0.02480179	-29.105223	3.08E-186	1.28E-184
TNFAIP3	34.4899713	-0.7167759	0.28459904	-2.5185465	0.01178403	0.02025685
IRF1	108.073767	-0.7126453	0.166047	-4.2918288	1.77E-05	4.17E-05
SLC7A5	1840.83922	-0.7124258	0.04515684	-15.7767	4.50E-56	6.24E-55

UGDH	666.933864	-0.709603	0.06597075	-10.756327	5.53E-27	3.67E-26
ERN1	72.9468972	-0.7057473	0.19553089	-3.6093905	0.00030692	0.00066731
PRDX1	12996.4828	-0.7039864	0.0244072	-28.843387	6.13E-183	2.39E-181
PTGER1	18.4275688	-0.7025576	0.39253435	-1.789799	0.07348622	0.11322322
SPINT2	19.2307201	-0.699371	0.39072705	-1.7899221	0.07346643	0.11322322
CCNE1	169.939919	-0.696036	0.1304823	-5.3343329	9.59E-08	2.71E-07
CYFIP1	500.591886	-0.6944669	0.0768439	-9.0373721	1.60E-19	8.08E-19
PGK1	28381.587	-0.689987	0.01705134	-40.465273	0	0
ITPR1	74.0050004	-0.6875389	0.19762938	-3.4789304	0.00050342	0.00105061
HIF1A	16917.9839	-0.6842449	0.01832335	-37.342779	3.32E-305	2.96E-303
SELL	19.9166879	-0.6798947	0.40756203	-1.6681993	0.09527618	0.1450057
KRT16	13.8678751	-0.6667625	0.50097008	-1.3309428	0.18320784	0.25690268
TAPBP	212.310614	-0.6657238	0.11910558	-5.5893583	2.28E-08	6.74E-08
PKM	40696.2432	-0.6656412	0.01965538	-33.86559	2.14E-251	1.48E-249
SKIL	1290.82292	-0.6561371	0.04957881	-13.234225	5.57E-40	4.76E-39
G6PD	1592.9585	-0.641792	0.04398437	-14.591365	3.19E-48	3.83E-47
MAFG	346.419336	-0.6414144	0.09171165	-6.9938155	2.68E-12	9.65E-12
TAP2	241.372075	-0.6382914	0.10878513	-5.8674508	4.43E-09	1.39E-08
HK2	182.705425	-0.633823	0.12747787	-4.9720238	6.63E-07	1.74E-06
FZD4	40.7996852	-0.6333383	0.26380645	-2.4007689	0.01636067	0.02766682
RELB	44.8905498	-0.6324182	0.25061457	-2.5234695	0.01162031	0.0200306
CD276	1606.79931	-0.6287103	0.04367827	-14.394124	5.63E-47	6.39E-46
ESR2	14.1426203	-0.6280048	0.46375621	-1.3541701	0.1756821	0.24802179
SERPINB5	16.6086959	-0.6252762	0.42111665	-1.4848052	0.13759543	0.20159138
ITPR3	89.8465332	-0.6213073	0.17644239	-3.521304	0.00042943	0.00090529
ZEB1	221.667669	-0.609425	0.1138434	-5.3531865	8.64E-08	2.45E-07
NUDT2	406.954755	-0.6077402	0.08403373	-7.232098	4.76E-13	1.83E-12
CD68	33.2839789	-0.605942	0.29039606	-2.0866055	0.03692381	0.0600012
EPAS1	49.3242012	-0.6049671	0.24520965	-2.4671421	0.01361963	0.02328397
HLA-E	428.389677	-0.6042193	0.08245508	-7.3278598	2.34E-13	9.24E-13
RNF111	638.602183	-0.5918476	0.07275541	-8.1347571	4.13E-16	1.80E-15
SMC3	1426.30729	-0.5901978	0.04654861	-12.679174	7.71E-37	6.17E-36
KMT2D	180.314593	-0.5867444	0.12841081	-4.5692756	4.89E-06	1.23E-05
IDO1	30.1195859	-0.5864565	0.31262395	-1.8759166	0.06066674	0.09628928
PDK1	332.763756	-0.5846026	0.09325853	-6.2686237	3.64E-10	1.19E-09
PTGER3	15.0907925	-0.5833989	0.4289185	-1.3601627	0.17377845	0.24644944
CD14	14.2617343	-0.5803349	0.43961439	-1.3200998	0.18680169	0.26092617
JAK1	354.090468	-0.5724368	0.09124846	-6.2733853	3.53E-10	1.16E-09
ACTG2	14.5800847	-0.5675682	0.43881448	-1.2934126	0.19586839	0.27160417
PGF	175.329965	-0.5630084	0.12677746	-4.4409185	8.96E-06	2.16E-05
AKT3	996.490342	-0.5593319	0.05463138	-10.238289	1.34E-24	8.09E-24
EIF4EBP1	3868.38824	-0.5588505	0.03081057	-18.138272	1.59E-73	2.92E-72
PDGFB	97.9533374	-0.5518865	0.17223631	-3.2042404	0.00135419	0.00269114
WWC1	380.336929	-0.5496076	0.08713833	-6.3073002	2.84E-10	9.37E-10
FGFR2	44.913832	-0.5429285	0.2485896	-2.1840353	0.02895965	0.04780641
SIN3A	693.481927	-0.5419299	0.0675299	-8.0250361	1.01E-15	4.31E-15
KIR3DL1	15.2317044	-0.5410815	0.43870606	-1.2333577	0.21744236	0.2975527
TAZ	135.044061	-0.5361443	0.14335655	-3.7399362	0.00018407	0.00040875
CD8B	15.0650253	-0.5233608	0.46561972	-1.1240091	0.26100916	0.35101232
RIMKLB	199.872666	-0.5207946	0.12607005	-4.1309934	3.61E-05	8.32E-05
GABPA	348.27064	-0.5091377	0.09316688	-5.4647931	4.63E-08	1.35E-07
IL27RA	39.1445368	-0.5086409	0.26975332	-1.8855781	0.05935184	0.09473983
LDHA	357.317897	-0.5043104	0.08996454	-5.6056582	2.07E-08	6.19E-08
IFNAR2	317.058379	-0.503945	0.09522589	-5.292101	1.21E-07	3.38E-07
EGF	122.018881	-0.5025012	0.1553448	-3.2347477	0.0012175	0.00243501
ALDOA	12248.2018	-0.4988188	0.01997239	-24.975416	1.13E-137	3.21E-136
CHMP2A	1756.00337	-0.495881	0.0429942	-11.533672	8.93E-31	6.40E-30
PDGFRA	751.849195	-0.4946033	0.06193782	-7.9854817	1.40E-15	5.90E-15
SMO	118.054272	-0.4910449	0.15444418	-3.1794329	0.00147564	0.00292316
FGFR1	112.52553	-0.489351	0.16165939	-3.0270496	0.00246953	0.00472696
PHB	164.500446	-0.4892913	0.13028774	-3.7554673	0.00017302	0.00038558
XRCC2	373.005184	-0.4805519	0.08857048	-5.425644	5.77E-08	1.67E-07
OAS3	102.976769	-0.4757753	0.17046748	-2.791003	0.0052545	0.00975835
MAP2K1	709.643991	-0.4751114	0.06719002	-7.0711601	1.54E-12	5.78E-12
PPAN	76.3041591	-0.4740088	0.19352561	-2.4493337	0.01431208	0.02433443
SNAI1	27.6300565	-0.4725844	0.31538444	-1.4984393	0.13401915	0.19770201
SAV1	514.709356	-0.4720216	0.07669836	-6.1542604	7.54E-10	2.43E-09

AKT1S1	246.665959	-0.4623274	0.10831687	-4.268286	1.97E-05	4.60E-05
SOD1	1970.61522	-0.4609842	0.04193286	-10.993387	4.11E-28	2.76E-27
TNFRSF17	14.7079178	-0.4596125	0.44037781	-1.0436776	0.29663457	0.39382972
JAK2	128.527471	-0.4517997	0.15025104	-3.0069656	0.0026387	0.00501996
FCRL2	25.7861881	-0.4500256	0.32980503	-1.3645203	0.17240388	0.24561648
ATG2B	270.475959	-0.4416574	0.10235846	-4.3148107	1.60E-05	3.78E-05
SRC	23.0261509	-0.439984	0.35935213	-1.2243811	0.22080849	0.3014978
ZMYM2	368.163418	-0.4379206	0.08817737	-4.9663604	6.82E-07	1.79E-06
IQGAP1	4054.97761	-0.431953	0.03067993	-14.079332	5.09E-45	5.29E-44
SQLE	592.083912	-0.4316158	0.06946761	-6.2131945	5.19E-10	1.69E-09
CXCL10	14.5445621	-0.430809	0.44374865	-0.9708401	0.33162789	0.4333125
IL15RA	14.5559287	-0.4294738	0.4432847	-0.9688443	0.3326229	0.4333125
STK4	419.117617	-0.4246585	0.08486845	-5.0037262	5.62E-07	1.49E-06
TFRC	3684.7449	-0.4184418	0.03055447	-13.694946	1.09E-42	1.01E-41
IRF3	234.866946	-0.4179026	0.11084492	-3.7701554	0.00016315	0.00036489
CD86	15.2234746	-0.4178243	0.42874157	-0.9745364	0.32979028	0.43233011
NF1	66.1060706	-0.4108389	0.20800304	-1.9751583	0.04825019	0.07779876
ERO1A	1494.3438	-0.4106358	0.04841653	-8.481315	2.23E-17	1.04E-16
SIGLEC5	17.074139	-0.4100397	0.41631502	-0.9849264	0.3246602	0.42740078
LAMB1	2176.95121	-0.409702	0.03972785	-10.312717	6.17E-25	3.93E-24
SERPINE1	3778.97895	-0.4014594	0.03436331	-11.682793	1.56E-31	1.16E-30
COL4A2	492.76251	-0.3994524	0.08028219	-4.9756037	6.50E-07	1.72E-06
LAMA4	125.684305	-0.3948177	0.14848728	-2.6589328	0.00783886	0.01405588
PCLAF	6725.28299	-0.3875281	0.0250684	-15.458833	6.58E-54	8.73E-53
RET	18.0298979	-0.3827494	0.40045729	-0.9557809	0.33918292	0.4409378
ABI1	223.114798	-0.3783349	0.11108951	-3.4056767	0.00066	0.00137281
TNF	16.8937799	-0.3768135	0.4036031	-0.933624	0.35049786	0.45281712
HUWE1	591.16278	-0.3762914	0.07366555	-5.1081061	3.25E-07	8.87E-07
PNOC	24.3754565	-0.3736938	0.33676257	-1.1096653	0.26714326	0.35771973
ATP7A	144.806613	-0.3655512	0.14372302	-2.5434423	0.01097662	0.01913328
BRAF	228.069887	-0.3580528	0.11054739	-3.2389074	0.00119989	0.00240749
PFKFB3	162.539366	-0.3575958	0.13049768	-2.7402466	0.00613931	0.01116889
SNAI2	218.519044	-0.352362	0.11351605	-3.1040719	0.00190877	0.00372137
WEE1	1279.26571	-0.3511168	0.0487207	-7.2067285	5.73E-13	2.19E-12
SQSTM1	1826.53051	-0.3294799	0.04553382	-7.2359391	4.62E-13	1.79E-12
LAT	16.1592788	-0.3283152	0.4283144	-0.7665284	0.44336195	0.55555743
NCR1	20.1537162	-0.3223493	0.37464427	-0.8604144	0.38956066	0.49923099
MUC1	28.0858784	-0.3125353	0.31917182	-0.979207	0.32747771	0.4302023
TPI1	4996.02737	-0.3023599	0.02721888	-11.108459	1.14E-28	7.91E-28
LCN2	35.2112213	-0.2997233	0.28314992	-1.0585323	0.28981283	0.38641711
KDM5A	382.645761	-0.2975896	0.08526654	-3.4901096	0.00048282	0.00101101
CD44	8544.06627	-0.291895	0.02471931	-11.808379	3.53E-32	2.69E-31
DICER1	437.753492	-0.2899883	0.0802311	-3.6144129	0.00030103	0.0006582
CHD8	442.999194	-0.2862333	0.07957183	-3.5971691	0.0003217	0.00069221
IGF1R	823.755917	-0.2761797	0.05912523	-4.6710971	3.00E-06	7.57E-06
B4GALNT1	434.29494	-0.2730959	0.08312239	-3.2854675	0.00101813	0.00205604
TPD52	1272.8241	-0.2563473	0.04902582	-5.228823	1.71E-07	4.71E-07
PCNA	1100.26852	-0.2544948	0.05138063	-4.9531265	7.30E-07	1.91E-06
EGLN3	235.781717	-0.2535537	0.10921645	-2.3215707	0.02025606	0.0340695
ASXL2	164.158876	-0.2463288	0.13218821	-1.8634706	0.06239608	0.0983211
STK11IP	118.415257	-0.2445592	0.15943428	-1.5339183	0.12504976	0.18590902
ATOX1	1393.5252	-0.2414555	0.04647761	-5.1950919	2.05E-07	5.60E-07
PLAU	11055.0007	-0.241405	0.02070051	-11.661794	2.00E-31	1.47E-30
NFKB1	572.421521	-0.2406885	0.07127844	-3.3767368	0.00073351	0.00149579
HSPB1	8370.68749	-0.2351033	0.02463137	-9.5448717	1.36E-21	7.59E-21
NKX3-1	29.7722581	-0.2341763	0.30551287	-0.7665022	0.44337756	0.55555743
MGA	344.402098	-0.2339783	0.09022339	-2.5933217	0.00950538	0.01685045
ARID4A	176.034219	-0.2332908	0.12838233	-1.8171564	0.06919317	0.10767217
EPC1	187.457403	-0.2310379	0.12445806	-1.8563512	0.06340352	0.09912731
PIK3R1	383.471053	-0.2293077	0.0866296	-2.6469902	0.00812117	0.01452038
MRPL19	1508.37212	-0.2262482	0.04490605	-5.038257	4.70E-07	1.26E-06
DNMT1	1768.63281	-0.2256145	0.04323228	-5.2186585	1.80E-07	4.95E-07
KRAS	606.571066	-0.223233	0.06872292	-3.2483057	0.00116095	0.00233687
CRK	744.06325	-0.2221454	0.0634966	-3.4985406	0.00046781	0.00098288
OSMR	1290.31771	-0.2159529	0.04848257	-4.4542392	8.42E-06	2.05E-05
SRM	1993.77172	-0.2120201	0.03913984	-5.4169899	6.06E-08	1.74E-07
GSR	1340.23191	-0.2100215	0.04772378	-4.4007725	1.08E-05	2.58E-05

CDK12	772.498262	-0.2084001	0.06231136	-3.3444957	0.00082432	0.00167006
PSMC4	992.81467	-0.2067041	0.05634261	-3.6686999	0.00024379	0.00053754
MTOR	323.962997	-0.2059304	0.09359747	-2.2001711	0.02779476	0.04600512
DKC1	887.16938	-0.2054159	0.05779954	-3.5539371	0.00037951	0.00080824
UBB	26583.5048	-0.2047575	0.01759912	-11.63453	2.75E-31	2.00E-30
ATF2	706.539106	-0.2018465	0.06405956	-3.1509187	0.00162758	0.00320381
VEGFC	142.858757	-0.1981602	0.13887868	-1.4268583	0.15362073	0.22087405
HLA-A	890.552974	-0.197292	0.05806763	-3.3976243	0.00067974	0.00140916
LAMTOR4	586.525	-0.1922419	0.07103524	-2.7062892	0.00680398	0.01230632
ABCC1	1274.66888	-0.1909583	0.04957854	-3.8516333	0.00011733	0.00026432
PFKP	3171.17509	-0.1909094	0.03310104	-5.7674745	8.05E-09	2.49E-08
GSTM4	166.982563	-0.1873594	0.13007474	-1.4403983	0.14975474	0.21581283
TLK2	633.982475	-0.1838147	0.0681457	-2.6973775	0.0069888	0.01260408
SLC1A5	3812.38415	-0.1791931	0.03017286	-5.9388815	2.87E-09	9.09E-09
TRAF6	285.821273	-0.1772513	0.09934852	-1.7841366	0.07440147	0.11435103
NDUFA2	2699.17873	-0.1654439	0.03506744	-4.717877	2.38E-06	6.12E-06
ITGAV	2649.10368	-0.1651853	0.03480714	-4.7457295	2.08E-06	5.38E-06
GABARAP	3589.21209	-0.1633251	0.0312332	-5.2292161	1.70E-07	4.71E-07
AKT2	1032.38155	-0.1626962	0.05336057	-3.0489963	0.00229607	0.00442207
SREBF1	4950.1316	-0.1622667	0.02826573	-5.7407577	9.43E-09	2.88E-08
EIF2AK3	246.42595	-0.1619006	0.11013665	-1.4699976	0.14156242	0.20639007
AGO4	110.138733	-0.1600836	0.16122196	-0.9929391	0.32073962	0.42313218
COX5B	4452.67801	-0.1577477	0.0298043	-5.2927834	1.20E-07	3.38E-07
DOCK1	1098.9907	-0.1549192	0.05241531	-2.9556088	0.00312053	0.00590063
ABCC4	177.526784	-0.1530941	0.13762665	-1.1123873	0.26597165	0.3569168
CCNB2	3429.56392	-0.1515285	0.03402125	-4.4539376	8.43E-06	2.05E-05
ATRX	1804.02186	-0.1514894	0.04200639	-3.6063426	0.00031054	0.00067284
OAZ1	10869.4454	-0.1491979	0.02118013	-7.0442375	1.86E-12	6.80E-12
CREBBP	428.804259	-0.1470802	0.08145288	-1.8057085	0.07096388	0.10987955
TXN2	1161.38232	-0.1454042	0.05277769	-2.7550311	0.00586866	0.01077071
RB1CC1	1350.36293	-0.1407665	0.04981211	-2.8259488	0.00471408	0.00883359
IDH1	2128.73793	-0.1363116	0.04029229	-3.3830692	0.00071681	0.00146703
MAPK14	1023.82207	-0.1314423	0.05449148	-2.4121626	0.01585821	0.02689
ADM	397.101763	-0.130766	0.08424603	-1.5521922	0.12061625	0.18092437
PUM1	830.179899	-0.1254883	0.0616897	-2.0341859	0.04193286	0.06778783
CCNT1	601.22089	-0.1210371	0.06907694	-1.7522071	0.0797382	0.12195254
MAPK3	271.501803	-0.1172807	0.10274145	-1.1415132	0.25365644	0.34334407
LYN	332.619792	-0.1131648	0.09280039	-1.2194437	0.22267584	0.30338367
SLC2A1	143.800093	-0.108533	0.14024616	-0.7738751	0.43900464	0.55229616
WASF2	1329.02017	-0.102593	0.04916247	-2.0868158	0.03690479	0.0600012
NSD1	484.180796	-0.0992573	0.07751672	-1.2804633	0.20038226	0.27663392
FBN1	744.193242	-0.0985292	0.06424751	-1.5335879	0.12513107	0.18590902
MTHFD2	5108.19666	-0.084537	0.02761491	-3.0612813	0.00220392	0.00425773
NCOR1	360.642652	-0.083302	0.09224882	-0.9030142	0.36651835	0.47156176
RRAGC	283.508074	-0.0813607	0.10431315	-0.7799659	0.43541095	0.54908536
ITGB1	5963.67312	-0.0694088	0.02521909	-2.7522328	0.00591904	0.01083133
ATF4	1383.86408	-0.0669801	0.04819941	-1.3896461	0.16463637	0.23508717
UBE2C	3415.62882	-0.0520055	0.03362807	-1.5464902	0.12198624	0.18210385
JAG1	1143.77863	-0.0430863	0.05106018	-0.8438333	0.39876257	0.50781193
BIRC5	4058.63778	-0.0285172	0.0303217	-0.940487	0.3469678	0.44918653
KPNA2	13431.794	-0.0263154	0.01950217	-1.3493559	0.17722267	0.24963193
KIF23	2065.76651	0.03865388	0.03884023	0.99520197	0.31963804	0.42257233
NDUFB1	3040.20001	0.05150002	0.03315726	1.55320501	0.12037417	0.18092437
RASA1	751.446852	0.05267599	0.06296754	0.83655788	0.40284113	0.51196103
KDM1A	1572.23277	0.06353478	0.04362039	1.45653864	0.14524378	0.21077237
AURKB	528.414215	0.06488277	0.07588162	0.8550525	0.39252208	0.50088708
CDK4	4274.61545	0.06580552	0.02841953	2.31550379	0.02058538	0.03453031
NME1	8453.89111	0.06752357	0.02286553	2.95307304	0.00314628	0.00593135
TBRG4	509.826716	0.07157867	0.0754838	0.94826529	0.34299442	0.44496573
GLUD1	868.18475	0.0745226	0.05889577	1.26533033	0.20575294	0.28279699
AURKA	1538.65017	0.07493327	0.04518748	1.65827494	0.09726199	0.14766783
VEGFB	294.89822	0.07625668	0.09780366	0.77969144	0.43557252	0.54908536
MSH2	1547.56319	0.07885645	0.04457676	1.76900355	0.07689328	0.11789044
MAP2K2	1006.81344	0.07947674	0.053959	1.47290979	0.14077536	0.20572325
RUNX1	885.933054	0.08271842	0.05844848	1.41523651	0.15699917	0.2252126
IL11	570.091454	0.08355609	0.07218953	1.15745439	0.24708676	0.33517857
POLR2A	530.674199	0.08602566	0.07606504	1.13094871	0.25807668	0.34781825

POS_A	17738.0436	0.08672648	0.04625508	1.87496135	0.06079804	0.09628928
ELOVL5	9605.30479	0.08930493	0.02260667	3.95037989	7.80E-05	0.00017835
POS_B	7281.63072	0.08954272	0.02534508	3.53294247	0.00041096	0.00087225
IFNAR1	515.522478	0.09070567	0.07608105	1.19222428	0.23317332	0.31699379
TCOF1	203.207769	0.09445087	0.11904628	0.79339623	0.42754701	0.54115483
ATM	647.190571	0.09588784	0.06625013	1.44736082	0.14779589	0.21397828
GLUD2	1454.89419	0.09593474	0.04670402	2.05410033	0.03996598	0.06477603
TUBG1	741.304085	0.09969479	0.06522577	1.52845707	0.12639909	0.18734688
ATXN7	139.014276	0.10311805	0.14278351	0.72219857	0.47017241	0.58096551
RBL1	432.863889	0.10313369	0.08221745	1.25440153	0.2096961	0.28758322
IFNGR2	616.415344	0.1048948	0.07281064	1.44065202	0.14968302	0.21581283
HDAC1	803.763132	0.1054331	0.06051306	1.74231961	0.08145253	0.12426988
PRIM1	1183.11005	0.10948085	0.05043493	2.17073484	0.02995122	0.04931283
ARID1A	422.252698	0.11080426	0.08395756	1.3197651	0.18691346	0.26092617
LAMTOR1	242.005853	0.11110123	0.11455317	0.96986605	0.33211327	0.4333125
PA2G4	4285.03346	0.11435986	0.03130715	3.65283481	0.00025936	0.00056986
TFDP1	523.649584	0.11772755	0.08075634	1.45781194	0.14489239	0.21075256
EZH2	818.408381	0.11846492	0.06058986	1.95519398	0.05056018	0.08131328
PRKACA	1229.06851	0.11872886	0.0532278	2.23057987	0.02570897	0.04266595
POS_C	2047.13572	0.12054396	0.04727374	2.54991374	0.01077496	0.01893964
AKT1	2478.37993	0.12889506	0.04056268	3.17767579	0.00148461	0.00293163
TXNRD2	278.751544	0.13058329	0.10006822	1.30494264	0.19191238	0.26671119
SMS	12259.8982	0.13254695	0.02074744	6.38859282	1.67E-10	5.56E-10
TET2	373.697965	0.13372604	0.08886584	1.50480817	0.1323734	0.19573698
GNAI3	2867.89857	0.13439831	0.03405407	3.94661574	7.93E-05	0.00018051
TMUB2	257.876967	0.13950093	0.11006217	1.26747397	0.20498587	0.28236464
LATS1	524.002991	0.14032467	0.07562482	1.85553723	0.06351955	0.09912731
NRDE2	231.497714	0.14727934	0.10869166	1.35501971	0.17541128	0.24802179
RFC3	1313.88255	0.14746574	0.04754369	3.101689	0.0019242	0.00372888
TBP	770.865363	0.15194781	0.06177141	2.45984061	0.01389987	0.02369814
CREB1	323.718219	0.15396121	0.09845117	1.56383322	0.11785674	0.17775839
ATG4B	538.209734	0.15755701	0.0729019	2.16121943	0.03067839	0.05037715
ATR	370.974321	0.16387368	0.0869826	1.88398229	0.05956737	0.09482153
DLL3	75.2136084	0.16438948	0.1910841	0.86029911	0.38962418	0.49923099
PARP2	678.648942	0.16541853	0.06499928	2.54492861	0.01093001	0.01913328
SOX2	833.107936	0.16620458	0.06042711	2.75049706	0.00595049	0.01085704
GSK3B	649.964515	0.16726234	0.06777341	2.467964	0.0135884	0.02328397
CTNNA1	4811.80935	0.17185031	0.02780209	6.18120207	6.36E-10	2.06E-09
APC	794.131093	0.17224583	0.06147323	2.80196467	0.00507924	0.00948937
TOX4	676.658809	0.17257299	0.06806077	2.53557198	0.01122639	0.01945907
HRAS	405.373891	0.17985458	0.08399181	2.14133484	0.03224705	0.05281406
WWTR1	2183.9591	0.18009071	0.03817155	4.71793014	2.38E-06	6.12E-06
PIK3CB	527.420935	0.1890754	0.0741431	2.55014156	0.01076792	0.01893964
TPX2	2792.00353	0.19319443	0.0339867	5.6844121	1.31E-08	3.96E-08
FZD7	183.817067	0.19744382	0.12763358	1.54695823	0.12187332	0.18210385
SDHA	537.996911	0.19842595	0.07372605	2.69139561	0.00711538	0.01279537
RAC3	248.482448	0.19975082	0.10594128	1.88548623	0.05936422	0.09473983
RRM2	6702.52423	0.21012631	0.02615292	8.034527	9.39E-16	4.01E-15
SKA1	406.049182	0.21382754	0.08407065	2.54342687	0.0109771	0.01913328
PIK3CD	145.448649	0.21442909	0.13714715	1.56349652	0.11793585	0.17775839
TEAD1	811.860765	0.21998648	0.05948664	3.69808214	0.00021724	0.00048069
MAPK8	325.75556	0.22254207	0.09772846	2.2771469	0.02277746	0.03810491
MX1	34.463096	0.22603772	0.28451449	0.79446819	0.42692292	0.54115483
YAP1	666.500119	0.22625053	0.06687922	3.38297199	0.00071706	0.00146703
KMT2A	546.924746	0.22760856	0.07286885	3.12353721	0.00178691	0.0035064
MAML2	298.209012	0.2298845	0.09757854	2.35589204	0.01847829	0.03116338
MYBL2	416.606083	0.23036047	0.08355612	2.75695519	0.00583424	0.01073912
SPHK2	31.307252	0.23556119	0.308804	0.76281781	0.44557206	0.55718831
ABCF1	956.697353	0.23599837	0.05543319	4.25734775	2.07E-05	4.80E-05
ROCK2	726.200608	0.23723239	0.06636925	3.57443224	0.00035099	0.00075006
MAP3K7	1109.81976	0.24600239	0.05246765	4.68864857	2.75E-06	7.00E-06
CTNNB1	5280.02227	0.24772699	0.02669158	9.28109243	1.68E-20	8.80E-20
RAD54L	229.292878	0.25105314	0.11082053	2.26540285	0.02348797	0.03918848
XBP1	577.524821	0.25703321	0.07287738	3.52692712	0.00042041	0.00088928
RBX1	2852.44833	0.25910657	0.03517416	7.36639065	1.75E-13	6.97E-13
EIF4G1	905.473769	0.25937627	0.05704408	4.54694495	5.44E-06	1.36E-05
STAT1	528.265374	0.26077262	0.07695893	3.3884649	0.00070285	0.00144745

CD47	780.84227	0.26727661	0.06085661	4.39190772	1.12E-05	2.68E-05
ERCC3	1347.31952	0.27354144	0.04786988	5.71426983	1.10E-08	3.35E-08
CHUK	609.422658	0.28010659	0.07108196	3.94061412	8.13E-05	0.00018442
MCM4	3336.69907	0.28676704	0.0320924	8.93566946	4.05E-19	2.00E-18
NCAPG	239.858771	0.28728294	0.10954658	2.62247293	0.00872942	0.01551897
NFE2L2	1077.02754	0.29279614	0.05286267	5.538807	3.05E-08	8.92E-08
PIK3R4	406.984423	0.29723399	0.08295895	3.58290443	0.0003398	0.00072863
KIF20A	1074.64392	0.30030502	0.05372627	5.58953825	2.28E-08	6.74E-08
LMNB1	2294.58991	0.3027424	0.0374603	8.08168637	6.39E-16	2.75E-15
LY96	470.534529	0.3084905	0.07837628	3.93601847	8.28E-05	0.0001873
PSMB7	2926.44378	0.31140701	0.03378598	9.21704803	3.05E-20	1.57E-19
CCNB1	10308.5672	0.31818181	0.02099152	15.1576386	6.75E-52	8.59E-51
CHEK2	174.738472	0.32346984	0.12741043	2.53880182	0.01112328	0.01933406
RAD54B	494.744239	0.32486671	0.07636506	4.25412745	2.10E-05	4.85E-05
GMPS	644.163815	0.32519415	0.07192804	4.52110409	6.15E-06	1.53E-05
PARP1	2282.2789	0.32589294	0.03988123	8.17158608	3.04E-16	1.34E-15
CDK6	551.314933	0.32635952	0.07252297	4.50008517	6.79E-06	1.67E-05
CCNF	792.644377	0.32770296	0.0602631	5.43787146	5.39E-08	1.56E-07
IL6ST	3397.29895	0.33120625	0.03304082	10.0241532	1.19E-23	7.03E-23
SF3B1	2100.63869	0.33284291	0.03898948	8.5367362	1.38E-17	6.53E-17
BUB1	2035.21096	0.33526193	0.03972499	8.43957219	3.19E-17	1.48E-16
SSRP1	2896.65513	0.3365318	0.03553693	9.46991793	2.80E-21	1.53E-20
CACYBP	3044.53502	0.33889467	0.03305641	10.2520122	1.16E-24	7.16E-24
ACSL3	543.829761	0.34021655	0.07279599	4.67356162	2.96E-06	7.51E-06
SETD2	491.796872	0.34293327	0.08049801	4.26014573	2.04E-05	4.76E-05
TCF7	26.2363178	0.34427183	0.32869847	1.04737886	0.29492488	0.39239472
CDK2	1853.77419	0.35072055	0.0427517	8.20366314	2.33E-16	1.03E-15
BRMS1	288.379313	0.35205289	0.10370062	3.39489679	0.00068655	0.00141856
PARVA	440.563893	0.35386411	0.07949863	4.45119768	8.54E-06	2.07E-05
ARID4B	592.823598	0.35884957	0.07072091	5.07416472	3.89E-07	1.05E-06
CDC20	1376.60589	0.36316048	0.04992926	7.27350095	3.50E-13	1.37E-12
CDK1	3451.77463	0.37008431	0.03120696	11.8590297	1.93E-32	1.49E-31
MLH1	681.220264	0.37243219	0.06453839	5.77070796	7.89E-09	2.45E-08
FOXM1	1731.66845	0.37316655	0.04299174	8.67995985	3.96E-18	1.90E-17
SOCS3	189.289383	0.3732873	0.12260942	3.04452383	0.00233049	0.00447454
LIG1	425.185865	0.37382342	0.08286588	4.51118633	6.45E-06	1.59E-05
E2F1	503.719425	0.38513752	0.07580491	5.08064074	3.76E-07	1.02E-06
ABCG2	14.6970524	0.38554697	0.45037487	0.85605792	0.39196575	0.50088708
PARD3	210.152752	0.38685173	0.119662	3.2328705	0.00122553	0.00244323
MET	1957.85556	0.39045796	0.04216591	9.26003942	2.04E-20	1.06E-19
CDH11	652.628212	0.39615196	0.06662963	5.94558235	2.75E-09	8.82E-09
NCF1	14.3326085	0.3964935	0.43724323	0.90680306	0.36451095	0.469948
IKBKG	62.055936	0.39665629	0.21378803	1.85537184	0.06354315	0.09912731
PIK3CA	786.37517	0.3994987	0.06061387	6.59087937	4.37E-11	1.50E-10
PLCB3	282.084317	0.40579553	0.10126513	4.00725848	6.14E-05	0.00014092
ATF7IP	591.166488	0.41933293	0.07230199	5.79974262	6.64E-09	2.07E-08
ADORA2B	1139.40066	0.42031729	0.05107885	8.22879257	1.89E-16	8.43E-16
SH3PXD2A	105.356367	0.42341345	0.16492322	2.5673368	0.0102483	0.01811598
PTGER4	25.8943053	0.42391168	0.33038465	1.28308531	0.19946219	0.2759743
MAPK1	1891.08868	0.42452359	0.04061002	10.4536655	1.41E-25	9.07E-25
CLSPN	1016.16151	0.43588518	0.0537782	8.10523968	5.26E-16	2.28E-15
EHHADH	111.106517	0.43600528	0.15770115	2.76475653	0.00569653	0.01051667
PLK1	3063.04368	0.43892587	0.03296061	13.3166801	1.85E-40	1.65E-39
TP53	1389.79268	0.43935416	0.04672852	9.4022696	5.34E-21	2.82E-20
H2AX	29.2427451	0.44211273	0.32447318	1.36255555	0.17302263	0.24593649
KAT2B	648.75647	0.44249147	0.06881248	6.43039531	1.27E-10	4.25E-10
TXN	7085.00353	0.44527956	0.02615439	17.0250427	5.36E-65	9.03E-64
MMP9	14.634436	0.44757214	0.44639491	1.00263718	0.31603595	0.41869731
TEAD2	192.459754	0.4521833	0.12565564	3.59859128	0.00031995	0.00069082
CDC25C	366.312662	0.4526034	0.09034048	5.0099732	5.44E-07	1.45E-06
MAPK13	17.4918499	0.45504073	0.39960014	1.13874015	0.25481155	0.34416106
ACKR2	16.3148442	0.45866841	0.42125648	1.08881032	0.27623754	0.36910541
RFC2	910.760186	0.46080582	0.05807028	7.93531221	2.10E-15	8.73E-15
CTNND1	734.376379	0.46647875	0.0637088	7.32204619	2.44E-13	9.58E-13
RB1	1276.29853	0.46864956	0.0495038	9.46694108	2.88E-21	1.56E-20
COP1	1114.43714	0.47392967	0.05245696	9.03463853	1.65E-19	8.21E-19
ITGB5	1296.957	0.47424954	0.05341072	8.87929564	6.73E-19	3.31E-18

IL7R	250.050543	0.47641763	0.10767345	4.42465269	9.66E-06	2.32E-05
EDN1	1664.03513	0.48315091	0.04286117	11.272462	1.79E-29	1.26E-28
TTK	687.391315	0.4860746	0.06526034	7.44823886	9.46E-14	3.81E-13
MCM2	1165.35326	0.49022723	0.05173547	9.47565035	2.65E-21	1.46E-20
MKI67	4291.75166	0.49952063	0.02914879	17.1369275	7.87E-66	1.36E-64
PAK2	1364.84466	0.50567686	0.04769592	10.6020992	2.91E-26	1.89E-25
TOP2A	7207.81527	0.50580617	0.02514148	20.1183913	5.09E-90	1.18E-88
KIF2C	1189.28239	0.5072533	0.05191585	9.77068366	1.50E-22	8.53E-22
FEN1	1725.90007	0.50759054	0.042018	12.0803108	1.34E-33	1.06E-32
ABL1	357.232801	0.50776096	0.09145427	5.55207493	2.82E-08	8.31E-08
MCL1	538.930814	0.5115882	0.07256086	7.05047097	1.78E-12	6.58E-12
CDCA8	1148.28447	0.5147932	0.05193139	9.91294843	3.66E-23	2.11E-22
STIP1	5186.50492	0.5178177	0.03145006	16.4647585	6.57E-61	1.03E-59
RAD51AP1	566.864485	0.52306447	0.07779331	6.72377195	1.77E-11	6.17E-11
RAF1	1169.46331	0.52336011	0.05165993	10.1308724	4.03E-24	2.42E-23
ASH1L	469.954306	0.53094571	0.0784053	6.77180868	1.27E-11	4.46E-11
LAMB3	90.7344047	0.53712557	0.1784758	3.00951479	0.00261665	0.00499325
RBBP5	602.099062	0.54333397	0.07342564	7.39978567	1.36E-13	5.46E-13
BRIP1	467.252356	0.5440256	0.07991846	6.80725869	9.95E-12	3.51E-11
PIK3R3	340.98163	0.54499018	0.09259312	5.88586069	3.96E-09	1.25E-08
MDM2	1492.43816	0.5507976	0.04590254	11.9992832	3.58E-33	2.80E-32
RPA3	4218.11244	0.55497714	0.02942433	18.8611647	2.38E-79	4.79E-78
FANCD2	147.547719	0.56200395	0.14786964	3.80067176	0.0001443	0.00032391
GPX1	4690.92463	0.56692693	0.02776663	20.4175651	1.17E-92	2.80E-91
MYD88	389.864057	0.57147564	0.08747491	6.53302324	6.45E-11	2.16E-10
LAMTOR2	1139.90766	0.5722856	0.05187293	11.0324528	2.66E-28	1.81E-27
CCNA2	2071.17293	0.57246333	0.04006597	14.2880192	2.60E-46	2.85E-45
SDC1	696.979654	0.57484138	0.06475277	8.87747971	6.84E-19	3.33E-18
KEAP1	509.446569	0.58150122	0.07606093	7.64520257	2.09E-14	8.56E-14
BUB1B	1213.72353	0.58559235	0.0500793	11.6933005	1.38E-31	1.04E-30
MYB	13.8516574	0.58961585	0.44944328	1.31188044	0.18956048	0.26403067
PBRM1	402.501488	0.59178547	0.08543921	6.92639233	4.32E-12	1.54E-11
NBL1	67.3897888	0.59906033	0.20332191	2.9463639	0.00321534	0.00604329
ITGA1	18.1876798	0.60078478	0.40417706	1.48643958	0.13716289	0.20159138
IDH2	432.731642	0.61087629	0.08171423	7.47576418	7.68E-14	3.11E-13
KLF5	146.958454	0.61293857	0.14090807	4.34991827	1.36E-05	3.23E-05
PRDX6	12202.473	0.61568393	0.02188156	28.1371111	3.45E-174	1.08E-172
RUVBL1	1856.46181	0.61663034	0.04093462	15.0637876	2.80E-51	3.50E-50
LAMC1	2099.57734	0.62211506	0.03999837	15.5535096	1.51E-54	2.04E-53
CD19	15.3092479	0.62225229	0.44141253	1.40968424	0.15863294	0.2270343
TSPAN1	26.3784773	0.6259258	0.33414364	1.87322373	0.0610375	0.09642379
PMAIP1	1336.53859	0.6269927	0.04824268	12.9966381	1.28E-38	1.08E-37
IL4R	16.2919928	0.64308553	0.48103921	1.33686717	0.181266	0.25475222
LIN9	301.370337	0.64826542	0.09830513	6.59442116	4.27E-11	1.47E-10
RAD51	301.930653	0.65239179	0.09838834	6.63078335	3.34E-11	1.16E-10
CHEK1	1550.46881	0.6597951	0.04583667	14.3944825	5.60E-47	6.39E-46
CAV1	2476.63289	0.66885556	0.03933721	17.0031284	7.79E-65	1.28E-63
CDC25A	419.200996	0.67031548	0.08438908	7.94315406	1.97E-15	8.25E-15
STING1	197.872256	0.68146927	0.1271392	5.36002496	8.32E-08	2.37E-07
BRCA2	304.788075	0.6936392	0.09848343	7.04320728	1.88E-12	6.82E-12
STAT4	13.9729154	0.69453434	0.46779627	1.48469407	0.13762488	0.20159138
DTL	667.275702	0.69495763	0.06780316	10.2496345	1.19E-24	7.27E-24
EGFR	253.994802	0.69667382	0.10626326	6.55611181	5.52E-11	1.86E-10
CCNE2	57.1646521	0.70050315	0.22573527	3.10320644	0.00191436	0.00372137
GTSE1	1263.9922	0.70287306	0.05013751	14.0189074	1.19E-44	1.20E-43
BCL2L1	764.440028	0.70306262	0.06333862	11.1000628	1.25E-28	8.60E-28
AMOTL2	40.894354	0.71057909	0.28056507	2.53267129	0.01131971	0.01956647
JUP	42.9437235	0.71941848	0.25812663	2.78707581	0.0053186	0.0098481
EXO1	438.697759	0.72488942	0.08257276	8.77879577	1.65E-18	7.99E-18
CDCA5	374.277925	0.73843653	0.08932732	8.26663739	1.38E-16	6.19E-16
HJURP	1042.90917	0.74616967	0.0546151	13.6623317	1.70E-42	1.56E-41
SKP2	1104.636	0.74683188	0.0541432	13.7936419	2.78E-43	2.67E-42
HMGA1	1301.88495	0.74800438	0.04982799	15.0117307	6.15E-51	7.53E-50
PTCH1	37.821793	0.75809046	0.27681942	2.73857395	0.00617063	0.01119323
PARP3	16.3363549	0.76760294	0.42465085	1.8076096	0.07066728	0.1096925
UCHL5	1380.54374	0.80233552	0.04728837	16.9668691	1.44E-64	2.31E-63
STK3	198.223184	0.80784042	0.12257955	6.59033617	4.39E-11	1.50E-10

EME1	367.869029	0.89595818	0.08948737	10.0121193	1.35E-23	7.86E-23
UBE2T	274.162146	0.89899872	0.10713571	8.39121446	4.81E-17	2.21E-16
AIMP2	709.650383	0.90765295	0.06991873	12.9815422	1.56E-38	1.30E-37
TOX3	146.250331	1.01443031	0.14365586	7.06153084	1.65E-12	6.15E-12
RFC4	1784.97988	1.0226833	0.04440072	23.033033	2.18E-117	5.66E-116
SLIT2	297.792469	1.06004913	0.10286445	10.3053016	6.67E-25	4.20E-24
TGFBR2	957.854229	1.06690505	0.05707603	18.6926982	5.68E-78	1.11E-76
TLR4	705.25112	1.07825869	0.0676087	15.94852	2.92E-57	4.14E-56
NEK2	255.532352	1.11601482	0.11343286	9.83854995	7.68E-23	4.40E-22
FBLIM1	55.1327202	1.19365269	0.23723758	5.03146542	4.87E-07	1.30E-06
DOCK2	122.256031	1.23073412	0.16321946	7.5403639	4.69E-14	1.91E-13
KDR	35.6524845	1.24853067	0.29195708	4.27641854	1.90E-05	4.46E-05
PDCD1LG2	92.3647242	1.25324176	0.18137166	6.90979917	4.85E-12	1.72E-11
TGFB2	906.612944	1.26494787	0.06152088	20.5612773	6.10E-94	1.52E-92
CD274	164.676908	1.28367923	0.1403255	9.14786848	5.81E-20	2.97E-19
ITGB8	2364.2972	1.32801317	0.0389864	34.0634951	2.56E-254	2.00E-252
NUF2	1848.50733	1.36875496	0.04448424	30.7694338	6.72E-208	3.23E-206
SPINT1	32.6884437	1.46172133	0.31046528	4.70816364	2.50E-06	6.39E-06
AXL	852.14976	1.62239843	0.06623506	24.4945553	1.69E-132	4.58E-131
THBS1	5821.3831	1.64615752	0.0282364	58.2991351	0	0
ALDH1A3	10251.5852	1.74923715	0.02394459	73.0535365	0	0
CFLAR	169.723046	2.0878167	0.14816729	14.0909419	4.32E-45	4.57E-44
CCND1	913.982319	2.08999452	0.06499307	32.1571906	7.01E-227	3.98E-225
ICAM1	26.7609939	2.11002332	0.37480559	5.62964738	1.81E-08	5.42E-08
IL7	42.4246206	2.13903545	0.30357759	7.04609137	1.84E-12	6.75E-12
FZD1	223.288048	2.21714369	0.13475994	16.4525436	8.04E-61	1.22E-59
GLUL	805.252074	2.22587711	0.07050145	31.572077	8.93E-219	4.64E-217
CDKN1A	213.313112	2.35913029	0.14340767	16.4505169	8.32E-61	1.24E-59
TP73	45.5145426	2.49293956	0.30131122	8.27363667	1.30E-16	5.88E-16
FLT1	39.2972395	2.56291068	0.32859214	7.79967119	6.21E-15	2.56E-14
THBS2	532.461735	2.65976127	0.09442834	28.1669827	1.48E-174	4.88E-173
NT5E	155.012142	2.73508583	0.17102536	15.9922825	1.45E-57	2.10E-56
CD34	449.385646	5.24584911	0.40878149	12.832893	1.07E-37	8.69E-37

Supplementary Table S3. Top 250 upregulated and top 250 downregulated genes in U251 CD97 knockout cells relative to wild type U251 cells

	baseMean	log2FoldChai	lfcSE	stat	pvalue	padj
ABCC1	1274.66888	-0.1909583	0.04957854	-3.8516333	0.00011733	0.00026432
ABCC4	177.526784	-0.1530941	0.13762665	-1.1123873	0.26597165	0.3569168
ABCG2	14.6970524	0.38554697	0.45037487	0.85605792	0.39196575	0.50088708
ABI1	223.114798	-0.3783349	0.11108951	-3.4056767	0.00066	0.00137281
ABL1	357.232801	0.50776096	0.09145427	5.55207493	2.82E-08	8.31E-08
ACKR2	16.3148442	0.45866841	0.42125648	1.08881032	0.27623754	0.36910541
ACSL3	543.829761	0.34021655	0.07279599	4.67356162	2.96E-06	7.51E-06
ACTG2	14.5800847	-0.5675682	0.43881448	-1.2934126	0.19586839	0.27160417
ADM	397.101763	-0.130766	0.08424603	-1.5521922	0.12061625	0.18092437
ADORA2A	33.2078229	-0.1095981	0.28586484	-0.3833913	0.70142966	0.78019984
ADORA2B	1139.40066	0.42031729	0.05107885	8.22879257	1.89E-16	8.43E-16
AGO2	67.8569367	0.0095626	0.20607983	0.04640239	0.96298953	0.97076813
AGO4	110.138733	-0.1600836	0.16122196	-0.9929391	0.32073962	0.42313218
AIMP2	709.650383	0.90765295	0.06991873	12.9815422	1.56E-38	1.30E-37
AKT1	2478.37993	0.12889506	0.04056268	3.17767579	0.00148461	0.00293163
AKT1S1	246.665959	-0.4623274	0.10831687	-4.268286	1.97E-05	4.60E-05
AKT2	1032.38155	-0.1626962	0.05336057	-3.0489963	0.00229607	0.00442207
AKT3	996.490342	-0.5593319	0.05463138	-10.238289	1.34E-24	8.09E-24
ALDH1A3	10251.5852	1.74923715	0.02394459	73.0535365	0	0
ALDOA	12248.2018	-0.4988188	0.01997239	-24.975416	1.13E-137	3.21E-136
AMOTL2	40.894354	0.71057909	0.28056507	2.53267129	0.01131971	0.01956647
APC	794.131093	0.17224583	0.06147323	2.80196467	0.00507924	0.00948937
AR	39.4046098	-0.0153018	0.26842471	-0.0570058	0.95454055	0.9688634
ARID1A	422.252698	0.11080426	0.08395756	1.3197651	0.18691346	0.26092617
ARID1B	190.004966	0.01783138	0.13070131	0.1364285	0.89148255	0.91947952
ARID2	551.1651	-0.0124983	0.07140619	-0.1750306	0.86105563	0.89999784
ARID4A	176.034219	-0.2332908	0.12838233	-1.8171564	0.06919317	0.10767217
ARID4B	592.823598	0.35884957	0.07072091	5.07416472	3.89E-07	1.05E-06
ASH1L	469.954306	0.53094571	0.0784053	6.77180868	1.27E-11	4.46E-11
ASXL2	164.158876	-0.2463288	0.13218821	-1.8634706	0.06239608	0.0983211
ATF2	706.539106	-0.2018465	0.06405956	-3.1509187	0.00162758	0.00320381
ATF4	1383.86408	-0.0669801	0.04819941	-1.3896461	0.16463637	0.23508717
ATF7IP	591.166488	0.41933293	0.07230199	5.79974262	6.64E-09	2.07E-08
ATG101	502.409864	-0.0200841	0.07488843	-0.2681875	0.78855496	0.83983019
ATG2B	270.475959	-0.4416574	0.10235846	-4.3148107	1.60E-05	3.78E-05
ATG4B	538.209734	0.15755701	0.0729019	2.16121943	0.03067839	0.05037715
ATM	647.190571	0.09588784	0.06625013	1.44736082	0.14779589	0.21397828
ATOX1	1393.5252	-0.2414555	0.04647761	-5.1950919	2.05E-07	5.60E-07
ATP7A	144.806613	-0.3655512	0.14372302	-2.5434423	0.01097662	0.01913328
ATR	370.974321	0.16387368	0.0869826	1.88398229	0.05956737	0.09482153
ATRX	1804.02186	-0.1514894	0.04200639	-3.6063426	0.00031054	0.00067284
ATXN7	139.014276	0.10311805	0.14278351	0.72219857	0.47017241	0.58096551
AURKA	1538.65017	0.07493327	0.04518748	1.65827494	0.09726199	0.14766783
AURKB	528.414215	0.06488277	0.07588162	0.8550525	0.39252208	0.50088708
AXL	852.14976	1.62239843	0.06623506	24.4945553	1.69E-132	4.58E-131
B2M	8222.17778	-0.7218615	0.02480179	-29.105223	3.08E-186	1.28E-184
B4GALNT1	434.29494	-0.2730959	0.08312239	-3.2854675	0.00101813	0.00205604
B4GALT1	2998.34516	-0.8691139	0.03455529	-25.151396	1.36E-139	4.05E-138
BAD	120.761082	0.10205867	0.15521255	0.65754132	0.51083291	0.62161457
BAX	1111.18982	-0.0320925	0.05446451	-0.5892367	0.55570252	0.65923645
BCAP31	1086.96698	-0.0221729	0.05166738	-0.4291478	0.6678157	0.75637444
BCL2	43.4523184	-0.1349583	0.2580807	-0.5229306	0.60102253	0.69483082
BCL2L1	764.440028	0.70306262	0.06333862	11.1000628	1.25E-28	8.60E-28
BHLHE40	767.524617	-0.899307	0.062978	-14.279702	2.93E-46	3.15E-45
BIRC5	4058.63778	-0.0285172	0.0303217	-0.940487	0.3469678	0.44918653
BLM	358.778292	0.0139045	0.08857004	0.15698874	0.87525372	0.91026387
BMPR1A	843.978161	-0.025971	0.06093764	-0.4261902	0.6699693	0.75637444
BMPR2	698.622911	0.03494615	0.06708302	0.52093879	0.60240941	0.69483082
BRAF	228.069887	-0.3580528	0.11054739	-3.2389074	0.00119989	0.00240749

BRCA1	565.639204	-0.0094344	0.0752374	-0.1253953	0.90021061	0.92390036
BRCA2	304.788075	0.6936392	0.09848343	7.04320728	1.88E-12	6.82E-12
BRIP1	467.252356	0.5440256	0.07991846	6.80725869	9.95E-12	3.51E-11
BRMS1	288.379313	0.35205289	0.10370062	3.39489679	0.00068655	0.00141856
BUB1	2035.21096	0.33526193	0.03972499	8.43957219	3.19E-17	1.48E-16
BUB1B	1213.72353	0.58559235	0.0500793	11.6933005	1.38E-31	1.04E-30
BYSL	283.893054	-0.068203	0.09885266	-0.6899458	0.49022825	0.60455025
CA9	163.892344	-3.7615101	0.20442313	-18.400609	1.30E-75	2.46E-74
CACYBP	3044.53502	0.33889467	0.03305641	10.2520122	1.16E-24	7.16E-24
CASP3	708.678018	0.02756961	0.06678001	0.41284224	0.6797222	0.76285369
CAV1	2476.63289	0.66885556	0.03933721	17.0031284	7.79E-65	1.28E-63
CBX2	82.6693364	-0.118638	0.18339098	-0.6469129	0.51768834	0.62847767
CCL2	4931.10724	-2.7626197	0.03403473	-81.170605	0	0
CCL5	17.2944995	-0.7774171	0.41776698	-1.8608868	0.06276016	0.0986457
CCNA2	2071.17293	0.57246333	0.04006597	14.2880192	2.60E-46	2.85E-45
CCNB1	10308.5672	0.31818181	0.02099152	15.1576386	6.75E-52	8.59E-51
CCNB2	3429.56392	-0.1515285	0.03402125	-4.4539376	8.43E-06	2.05E-05
CCND1	913.982319	2.08999452	0.06499307	32.1571906	7.01E-227	3.98E-225
CCNE1	169.939919	-0.696036	0.1304823	-5.3343329	9.59E-08	2.71E-07
CCNE2	57.1646521	0.70050315	0.22573527	3.10320644	0.00191436	0.00372137
CCNF	792.644377	0.32770296	0.0602631	5.43787146	5.39E-08	1.56E-07
CCNK	1021.38749	4.55E-05	0.05557624	0.00081831	0.99934708	0.99985843
CCNT1	601.22089	-0.1210371	0.06907694	-1.7522071	0.0797382	0.12195254
CCS	292.194222	-0.0356407	0.09855797	-0.3616212	0.71763509	0.79044303
CD14	14.2617343	-0.5803349	0.43961439	-1.3200998	0.18680169	0.26092617
CD163	19.8314256	-0.2791175	0.37540626	-0.7435078	0.45717429	0.56941468
CD19	15.3092479	0.62225229	0.44141253	1.40968424	0.15863294	0.2270343
CD274	164.676908	1.28367923	0.1403255	9.14786848	5.81E-20	2.97E-19
CD276	1606.79931	-0.6287103	0.04367827	-14.394124	5.63E-47	6.39E-46
CD28	13.8993947	-0.746569	0.47037438	-1.5871803	0.11247181	0.17034566
CD300A	18.014877	0.1461889	0.39656126	0.3686414	0.71239504	0.78818174
CD33	16.202512	-0.2739431	0.40793173	-0.6715416	0.50187554	0.61526589
CD34	449.385646	5.24584911	0.40878149	12.832893	1.07E-37	8.69E-37
CD3E	25.5523525	0.09418454	0.33244215	0.28331107	0.77693839	0.83443986
CD40LG	16.3265632	0.10514669	0.42425468	0.24783861	0.80425928	0.85483465
CD44	8544.06627	-0.291895	0.02471931	-11.808379	3.53E-32	2.69E-31
CD47	780.84227	0.26727661	0.06085661	4.39190772	1.12E-05	2.68E-05
CD68	33.2839789	-0.605942	0.29039606	-2.0866055	0.03692381	0.0600012
CD80	20.8226766	-0.2223058	0.36393461	-0.6108399	0.54130558	0.6495667
CD86	15.2234746	-0.4178243	0.42874157	-0.9745364	0.32979028	0.43233011
CD8B	15.0650253	-0.5233608	0.46561972	-1.1240091	0.26100916	0.35101232
CDC20	1376.60589	0.36316048	0.04992926	7.27350095	3.50E-13	1.37E-12
CDC25A	419.200996	0.67031548	0.08438908	7.94315406	1.97E-15	8.25E-15
CDC25C	366.312662	0.4526034	0.09034048	5.0099732	5.44E-07	1.45E-06
CDCA5	374.277925	0.73843653	0.08932732	8.26663739	1.38E-16	6.19E-16
CDCA8	1148.28447	0.5147932	0.05193139	9.91294843	3.66E-23	2.11E-22
CDH1	13.9797706	0.13295854	0.45235311	0.29392644	0.76881412	0.82713795
CDH11	652.628212	0.39615196	0.06662963	5.94558235	2.75E-09	8.82E-09
CDK1	3451.77463	0.37008431	0.03120696	11.8590297	1.93E-32	1.49E-31
CDK12	772.498262	-0.2084001	0.06231136	-3.3444957	0.00082432	0.00167006
CDK2	1853.77419	0.35072055	0.0427517	8.20366314	2.33E-16	1.03E-15
CDK4	4274.61545	0.06580552	0.02841953	2.31550379	0.02058538	0.03453031
CDK6	551.314933	0.32635952	0.07252297	4.50008517	6.79E-06	1.67E-05
CDKN1A	213.313112	2.35913029	0.14340767	16.4505169	8.32E-61	1.24E-59
CDKN2A	27.4804757	0.13433616	0.32120401	0.4182269	0.67578123	0.76116875
CEACAM1	18.7771007	0.21586574	0.38403009	0.56210633	0.57404357	0.67079249
CEACAM3	24.2789366	-1.7662501	0.39453024	-4.4768433	7.58E-06	1.85E-05
CENPA	359.789049	-0.0061611	0.08915978	-0.0691013	0.94490901	0.96501345
CFLAR	169.723046	2.0878167	0.14816729	14.0909419	4.32E-45	4.57E-44
CHD8	442.999194	-0.2862333	0.07957183	-3.5971691	0.0003217	0.00069221
CHEK1	1550.46881	0.6597951	0.04583667	14.3944825	5.60E-47	6.39E-46

CHEK2	174.738472	0.32346984	0.12741043	2.53880182	0.01112328	0.01933406
CHMP2A	1756.00337	-0.495881	0.0429942	-11.533672	8.93E-31	6.40E-30
CHUK	609.422658	0.28010659	0.07108196	3.94061412	8.13E-05	0.00018442
CLOCK	303.552225	-0.0703186	0.09717084	-0.7236594	0.46927485	0.58096551
CLSPN	1016.16151	0.43588518	0.0537782	8.10523968	5.26E-16	2.28E-15
COL4A1	140.545042	-0.9419538	0.14350633	-6.5638481	5.24E-11	1.78E-10
COL4A2	492.76251	-0.3994524	0.08028219	-4.9756037	6.50E-07	1.72E-06
COL6A1	883.954574	-2.1907719	0.06584697	-33.270657	1.03E-242	6.40E-241
COL6A2	82.0748802	-1.1540222	0.19429311	-5.9395942	2.86E-09	9.09E-09
COL6A3	103.629754	-1.8217734	0.18171713	-10.025326	1.18E-23	7.01E-23
COP1	1114.43714	0.47392967	0.05245696	9.03463853	1.65E-19	8.21E-19
COX5B	4452.67801	-0.1577477	0.0298043	-5.2927834	1.20E-07	3.38E-07
CPT2	358.365953	0.06006139	0.08935967	0.67213084	0.50150041	0.61526589
CREB1	323.718219	0.15396121	0.09845117	1.56383322	0.11785674	0.17775839
CREBBP	428.804259	-0.1470802	0.08145288	-1.8057085	0.07096388	0.10987955
CRK	744.06325	-0.2221454	0.0634966	-3.4985406	0.00046781	0.00098288
CSF1R	15.302732	0.06257003	0.45763701	0.13672415	0.89124884	0.91947952
CSF2RA	33.1022363	-2.6480257	0.37397778	-7.0807034	1.43E-12	5.42E-12
CTNNA1	4811.80935	0.17185031	0.02780209	6.18120207	6.36E-10	2.06E-09
CTNNB1	5280.02227	0.24772699	0.02669158	9.28109243	1.68E-20	8.80E-20
CTNND1	734.376379	0.46647875	0.0637088	7.32204619	2.44E-13	9.58E-13
CTSW	15.3976485	-0.2549942	0.42839698	-0.5952288	0.55169051	0.65572358
CXCL10	14.5445621	-0.430809	0.44374865	-0.9708401	0.33162789	0.4333125
CXCR2	13.9101837	-0.1094313	0.47177926	-0.2319544	0.81657344	0.86071255
CXCR4	37.5374302	-1.6341729	0.30177859	-5.4151385	6.12E-08	1.75E-07
CXXC5	143.904323	-1.7999157	0.15687669	-11.473443	1.79E-30	1.27E-29
CYBB	16.8973347	-0.1486207	0.407388	-0.3648137	0.71525046	0.78994034
CYFIP1	500.591886	-0.6944669	0.0768439	-9.0373721	1.60E-19	8.08E-19
DICER1	437.753492	-0.2899883	0.0802311	-3.6144129	0.00030103	0.0006582
DKC1	887.16938	-0.2054159	0.05779954	-3.5539371	0.00037951	0.00080824
DLL3	75.2136084	0.16438948	0.1910841	0.86029911	0.38962418	0.49923099
DNA2	381.116607	-0.0517818	0.08629486	-0.6000564	0.54846864	0.65572358
DNMT1	1768.63281	-0.2256145	0.04323228	-5.2186585	1.80E-07	4.95E-07
DOCK1	1098.9907	-0.1549192	0.05241531	-2.9556088	0.00312053	0.00590063
DOCK2	122.256031	1.23073412	0.16321946	7.5403639	4.69E-14	1.91E-13
DTL	667.275702	0.69495763	0.06780316	10.2496345	1.19E-24	7.27E-24
DUSP6	513.312892	0.03531935	0.07578216	0.46606416	0.64116957	0.73142562
E2F1	503.719425	0.38513752	0.07580491	5.08064074	3.76E-07	1.02E-06
E2F2	24.985732	-0.0921779	0.34251598	-0.2691199	0.78783744	0.83983019
EDN1	1664.03513	0.48315091	0.04286117	11.272462	1.79E-29	1.26E-28
EGF	122.018881	-0.5025012	0.1553448	-3.2347477	0.0012175	0.00243501
EGFR	253.994802	0.69667382	0.10626326	6.55611181	5.52E-11	1.86E-10
EGLN3	235.781717	-0.2535537	0.10921645	-2.3215707	0.02025606	0.0340695
EHHADH	111.106517	0.43600528	0.15770115	2.76475653	0.00569653	0.01051667
EIF2AK3	246.42595	-0.1619006	0.11013665	-1.4699976	0.14156242	0.20639007
EIF4EBP1	3868.38824	-0.5588505	0.03081057	-18.138272	1.59E-73	2.92E-72
EIF4G1	905.473769	0.25937627	0.05704408	4.54694495	5.44E-06	1.36E-05
ELOVL5	9605.30479	0.08930493	0.02260667	3.95037989	7.80E-05	0.00017835
EME1	367.869029	0.89595818	0.08948737	10.0121193	1.35E-23	7.86E-23
ENTPD1	20.5824796	0.21598883	0.37630685	0.57396996	0.56598813	0.66723874
EOMES	29.4213079	-0.2024816	0.30771002	-0.6580272	0.51052068	0.62161457
EP300	330.531803	-0.0541108	0.09226864	-0.5864483	0.55757428	0.6602018
EPAS1	49.3242012	-0.6049671	0.24520965	-2.4671421	0.01361963	0.02328397
EPC1	187.457403	-0.2310379	0.12445806	-1.8563512	0.06340352	0.09912731
ERBB2	89.066062	-0.1093102	0.17850611	-0.6123613	0.54029873	0.6495667
ERBB3	16.8481233	0.06838662	0.41228854	0.16587078	0.86825864	0.90449648
ERN1	72.9468972	-0.7057473	0.19553089	-3.6093905	0.00030692	0.00066731
ERO1A	1494.3438	-0.4106358	0.04841653	-8.481315	2.23E-17	1.04E-16
ESR2	14.1426203	-0.6280048	0.46375621	-1.3541701	0.1756821	0.24802179
EXO1	438.697759	0.72488942	0.08257276	8.77879577	1.65E-18	7.99E-18
EZH2	818.408381	0.11846492	0.06058986	1.95519398	0.05056018	0.08131328

FANCA	186.592778	0.02970411	0.12256998	0.24234411	0.80851354	0.85655763
FANCD2	147.547719	0.56200395	0.14786964	3.80067176	0.0001443	0.00032391
FANCI	1028.89685	-0.7828406	0.05470348	-14.310619	1.88E-46	2.09E-45
FAS	19.0792592	-1.2304803	0.39503504	-3.1148637	0.0018403	0.00359983
FASLG	15.1849263	-0.1654383	0.42364769	-0.390509	0.69616018	0.77710903
FBLIM1	55.1327202	1.19365269	0.23723758	5.03146542	4.87E-07	1.30E-06
FBLN2	13.8244551	-0.3107206	0.47277959	-0.6572209	0.5110389	0.62161457
FBN1	744.193242	-0.0985292	0.06424751	-1.5335879	0.12513107	0.18590902
FCRL2	25.7861881	-0.4500256	0.32980503	-1.3645203	0.17240388	0.24561648
FEN1	1725.90007	0.50759054	0.042018	12.0803108	1.34E-33	1.06E-32
FGFBP1	26.3164287	-2.1314535	0.37008059	-5.7594307	8.44E-09	2.59E-08
FGFR1	112.52553	-0.489351	0.16165939	-3.0270496	0.00246953	0.00472696
FGFR2	44.913832	-0.5429285	0.2485896	-2.1840353	0.02895965	0.04780641
FGFR3	24.0242164	-0.7955486	0.35585379	-2.2356052	0.02537765	0.0422284
FLT1	39.2972395	2.56291068	0.32859214	7.79967119	6.21E-15	2.56E-14
FOS	183.186323	-1.0672711	0.12710049	-8.3970655	4.58E-17	2.12E-16
FOXA1	20.4685955	0.05527998	0.36626072	0.15093068	0.8800304	0.91370877
FOXM1	1731.66845	0.37316655	0.04299174	8.67995985	3.96E-18	1.90E-17
FOXP1	514.57239	-1.0532266	0.08132901	-12.950196	2.34E-38	1.92E-37
FPR1	15.1041121	-1.3593615	0.45579788	-2.9823778	0.00286019	0.0054248
FRMD6	194.222195	-0.094702	0.12570939	-0.7533405	0.45124535	0.56315419
FSTL1	7082.07601	-1.0663418	0.02778251	-38.381769	0	0
FZD1	223.288048	2.21714369	0.13475994	16.4525436	8.04E-61	1.22E-59
FZD4	40.7996852	-0.6333383	0.26380645	-2.4007689	0.01636067	0.02766682
FZD7	183.817067	0.19744382	0.12763358	1.54695823	0.12187332	0.18210385
FZD8	35.6619827	-0.1712443	0.28763524	-0.5953521	0.55160812	0.65572358
GABARAP	3589.21209	-0.1633251	0.0312332	-5.2292161	1.70E-07	4.71E-07
GABPA	348.27064	-0.5091377	0.09316688	-5.4647931	4.63E-08	1.35E-07
GALNT3	17.4648196	0.12632717	0.39722423	0.31802483	0.7504661	0.8158132
GLUD1	868.18475	0.0745226	0.05889577	1.26533033	0.20575294	0.28279699
GLUD2	1454.89419	0.09593474	0.04670402	2.05410033	0.03996598	0.06477603
GLUL	805.252074	2.22587711	0.07050145	31.572077	8.93E-219	4.64E-217
GMPS	644.163815	0.32519415	0.07192804	4.52110409	6.15E-06	1.53E-05
GNAI3	2867.89857	0.13439831	0.03405407	3.94661574	7.93E-05	0.00018051
GNLY	15.2153704	-0.0429841	0.44356674	-0.0969055	0.92280142	0.94553052
GPNMB	160.325057	-2.1514614	0.16162037	-13.311821	1.98E-40	1.74E-39
GPX1	4690.92463	0.56692693	0.02776663	20.4175651	1.17E-92	2.80E-91
GSK3B	649.964515	0.16726234	0.06777341	2.467964	0.0135884	0.02328397
GSR	1340.23191	-0.2100215	0.04772378	-4.4007725	1.08E-05	2.58E-05
GSTM4	166.982563	-0.1873594	0.13007474	-1.4403983	0.14975474	0.21581283
GTSE1	1263.9922	0.70287306	0.05013751	14.0189074	1.19E-44	1.20E-43
GZMA	18.335866	-0.0139071	0.38376884	-0.0362383	0.97109232	0.97735743
GZMK	13.7254069	-0.1398816	0.46543595	-0.3005388	0.7637662	0.82399256
H2AX	29.2427451	0.44211273	0.32447318	1.36255555	0.17302263	0.24593649
HAVCR2	15.499299	0.02377473	0.42088882	0.05648696	0.95495388	0.9688634
HDAC1	803.763132	0.1054331	0.06051306	1.74231961	0.08145253	0.12426988
HIF1A	16917.9839	-0.6842449	0.01832335	-37.342779	3.32E-305	2.96E-303
HJURP	1042.90917	0.74616967	0.0546151	13.6623317	1.70E-42	1.56E-41
HK2	182.705425	-0.633823	0.12747787	-4.9720238	6.63E-07	1.74E-06
HLA-A	890.552974	-0.197292	0.05806763	-3.3976243	0.00067974	0.00140916
HLA-B	102.134456	-0.7995886	0.17344409	-4.6100655	4.03E-06	1.01E-05
HLA-C	50.6981749	-1.1212644	0.24845518	-4.5129442	6.39E-06	1.58E-05
HLA-E	428.389677	-0.6042193	0.08245508	-7.3278598	2.34E-13	9.24E-13
HMGA1	1301.88495	0.74800438	0.04982799	15.0117307	6.15E-51	7.53E-50
HMOX1	303.379714	-1.592808	0.10921847	-14.583687	3.57E-48	4.20E-47
HRAS	405.373891	0.17985458	0.08399181	2.14133484	0.03224705	0.05281406
HSPB1	8370.68749	-0.2351033	0.02463137	-9.5448717	1.36E-21	7.59E-21
HUWE1	591.16278	-0.3762914	0.07366555	-5.1081061	3.25E-07	8.87E-07
ICAM1	26.7609939	2.11002332	0.37480559	5.62964738	1.81E-08	5.42E-08
IDH1	2128.73793	-0.1363116	0.04029229	-3.3830692	0.00071681	0.00146703
IDH2	432.731642	0.61087629	0.08171423	7.47576418	7.68E-14	3.11E-13

IDO1	30.1195859	-0.5864565	0.31262395	-1.8759166	0.06066674	0.09628928
IFNAR1	515.522478	0.09070567	0.07608105	1.19222428	0.23317332	0.31699379
IFNAR2	317.058379	-0.503945	0.09522589	-5.292101	1.21E-07	3.38E-07
IFNGR2	616.415344	0.1048948	0.07281064	1.44065202	0.14968302	0.21581283
IGF1R	823.755917	-0.2761797	0.05912523	-4.6710971	3.00E-06	7.57E-06
IGFBP3	171.101091	-2.0499906	0.14573972	-14.066108	6.14E-45	6.28E-44
IKBKG	62.055936	0.39665629	0.21378803	1.85537184	0.06354315	0.09912731
IL10RA	15.2585192	-0.1582427	0.43996986	-0.3596671	0.71909609	0.79044303
IL11	570.091454	0.08355609	0.07218953	1.15745439	0.24708676	0.33517857
IL15	115.427275	-0.0979885	0.15989408	-0.6128336	0.53998637	0.6495667
IL15RA	14.5559287	-0.4294738	0.4432847	-0.9688443	0.3326229	0.4333125
IL22RA2	20.1872954	0.1956367	0.37504342	0.52163748	0.60192277	0.69483082
IL27RA	39.1445368	-0.5086409	0.26975332	-1.8855781	0.05935184	0.09473983
IL4R	16.2919928	0.64308553	0.48103921	1.33686717	0.181266	0.25475222
IL6	67.8583224	0.06659293	0.20100147	0.33130571	0.74041358	0.80772391
IL6ST	3397.29895	0.33120625	0.03304082	10.0241532	1.19E-23	7.03E-23
IL7	42.4246206	2.13903545	0.30357759	7.04609137	1.84E-12	6.75E-12
IL7R	250.050543	0.47641763	0.10767345	4.42465269	9.66E-06	2.32E-05
IQGAP1	4054.97761	-0.431953	0.03067993	-14.079332	5.09E-45	5.29E-44
IRF1	108.073767	-0.7126453	0.166047	-4.2918288	1.77E-05	4.17E-05
IRF3	234.866946	-0.4179026	0.11084492	-3.7701554	0.00016315	0.00036489
ITGA1	18.1876798	0.60078478	0.40417706	1.48643958	0.13716289	0.20159138
ITGA5	505.069571	-1.0995905	0.07875299	-13.962525	2.64E-44	2.61E-43
ITGAV	2649.10368	-0.1651853	0.03480714	-4.7457295	2.08E-06	5.38E-06
ITGB1	5963.67312	-0.0694088	0.02521909	-2.7522328	0.00591904	0.01083133
ITGB2	18.3892917	-0.1668053	0.38898545	-0.4288215	0.66805316	0.75637444
ITGB3	101.673726	-3.0527641	0.22628707	-13.49067	1.77E-41	1.61E-40
ITGB5	1296.957	0.47424954	0.05341072	8.87929564	6.73E-19	3.31E-18
ITGB8	2364.2972	1.32801317	0.0389864	34.0634951	2.56E-254	2.00E-252
ITPR1	74.0050004	-0.6875389	0.19762938	-3.4789304	0.00050342	0.00105061
ITPR3	89.8465332	-0.6213073	0.17644239	-3.521304	0.00042943	0.00090529
JAG1	1143.77863	-0.0430863	0.05106018	-0.8438333	0.39876257	0.50781193
JAK1	354.090468	-0.5724368	0.09124846	-6.2733853	3.53E-10	1.16E-09
JAK2	128.527471	-0.4517997	0.15025104	-3.0069656	0.0026387	0.00501996
JAK3	25.4487045	0.18634927	0.32894158	0.56651174	0.57104595	0.67079249
JMJD1C	271.606245	0.05078136	0.10144669	0.50057187	0.61667247	0.70606168
JUN	820.396598	-1.1853262	0.06223176	-19.046966	6.96E-81	1.45E-79
JUP	42.9437235	0.71941848	0.25812663	2.78707581	0.0053186	0.0098481
KAT2B	648.75647	0.44249147	0.06881248	6.43039531	1.27E-10	4.25E-10
KAT6A	703.380065	0.02263649	0.06607542	0.34258559	0.73191025	0.79984587
KAT6B	178.68228	0.0272961	0.12766501	0.21381032	0.83069497	0.87118263
KDM1A	1572.23277	0.06353478	0.04362039	1.45653864	0.14524378	0.21077237
KDM3B	685.601326	-0.0336542	0.06637951	-0.5069969	0.61215703	0.70218012
KDM5A	382.645761	-0.2975896	0.08526654	-3.4901096	0.00048282	0.00101101
KDM6A	169.063156	-0.0866955	0.12856764	-0.6743181	0.50010907	0.61526589
KDR	35.6524845	1.24853067	0.29195708	4.27641854	1.90E-05	4.46E-05
KEAP1	509.446569	0.58150122	0.07606093	7.64520257	2.09E-14	8.56E-14
KIF20A	1074.64392	0.30030502	0.05372627	5.58953825	2.28E-08	6.74E-08
KIF23	2065.76651	0.03865388	0.03884023	0.99520197	0.31963804	0.42257233
KIF2C	1189.28239	0.5072533	0.05191585	9.77068366	1.50E-22	8.53E-22
KIR2DL4	16.6481639	-0.0088059	0.4078301	-0.021592	0.9827734	0.98593344
KIR2DS4	16.8874916	-0.0968262	0.40498295	-0.2390871	0.81103802	0.85777581
KIR3DL1	15.2317044	-0.5410815	0.43870606	-1.2333577	0.21744236	0.2975527
KIR3DL2	17.9859218	0.19770882	0.38896182	0.50829878	0.61124382	0.70218012
KIT	14.7090673	0.31821258	0.43984939	0.72345804	0.46939852	0.58096551
KLF5	146.958454	0.61293857	0.14090807	4.34991827	1.36E-05	3.23E-05
KLRB1	19.0623364	-0.214133	0.38033936	-0.563005	0.57343149	0.67079249
KLRG1	15.1571224	-0.0969175	0.44395509	-0.2183047	0.82719172	0.87043446
KMT2A	546.924746	0.22760856	0.07286885	3.12353721	0.00178691	0.0035064
KMT2D	180.314593	-0.5867444	0.12841081	-4.5692756	4.89E-06	1.23E-05
KPNA2	13431.794	-0.0263154	0.01950217	-1.3493559	0.17722267	0.24963193

KRAS	606.571066	-0.223233	0.06872292	-3.2483057	0.00116095	0.00233687
KRT16	13.8678751	-0.6667625	0.50097008	-1.3309428	0.18320784	0.25690268
LAIR1	28.6281384	-1.6687761	0.33984468	-4.9104084	9.09E-07	2.36E-06
LAMA4	125.684305	-0.3948177	0.14848728	-2.6589328	0.00783886	0.01405588
LAMB1	2176.95121	-0.409702	0.03972785	-10.312717	6.17E-25	3.93E-24
LAMB3	90.7344047	0.53712557	0.1784758	3.00951479	0.00261665	0.00499325
LAMC1	2099.57734	0.62211506	0.03999837	15.5535096	1.51E-54	2.04E-53
LAMC2	36.2282305	-1.027848	0.2844186	-3.6138564	0.00030168	0.0006582
LAMTOR1	242.005853	0.11110123	0.11455317	0.96986605	0.33211327	0.4333125
LAMTOR2	1139.90766	0.5722856	0.05187293	11.0324528	2.66E-28	1.81E-27
LAMTOR4	586.525	-0.1922419	0.07103524	-2.7062892	0.00680398	0.01230632
LAMTOR5	13.8705887	0.23865002	0.44342616	0.53819562	0.590442	0.68738024
LAT	16.1592788	-0.3283152	0.4283144	-0.7665284	0.44336195	0.55555743
LATS1	524.002991	0.14032467	0.07562482	1.85553723	0.06351955	0.09912731
LCN2	35.2112213	-0.2997233	0.28314992	-1.0585323	0.28981283	0.38641711
LDHA	357.317897	-0.5043104	0.08996454	-5.6056582	2.07E-08	6.19E-08
LIG1	425.185865	0.37382342	0.08286588	4.51118633	6.45E-06	1.59E-05
LIN9	301.370337	0.64826542	0.09830513	6.59442116	4.27E-11	1.47E-10
LMNB1	2294.58991	0.3027424	0.0374603	8.08168637	6.39E-16	2.75E-15
LOX	837.011771	-0.8501562	0.06101412	-13.933763	3.95E-44	3.85E-43
LRRC32	21.3823423	-0.0200824	0.37187846	-0.0540026	0.95693311	0.9688634
LTBP1	1566.55825	-1.9960029	0.05189537	-38.462061	0	0
LTBR	238.121757	-0.8040281	0.11315318	-7.1056609	1.20E-12	4.56E-12
LY96	470.534529	0.3084905	0.07837628	3.93601847	8.28E-05	0.0001873
LYN	332.619792	-0.1131648	0.09280039	-1.2194437	0.22267584	0.30338367
MAFG	346.419336	-0.6414144	0.09171165	-6.9938155	2.68E-12	9.65E-12
MAGEC1	16.0241008	0.11222644	0.41376075	0.27123512	0.78621021	0.83983019
MAML2	298.209012	0.2298845	0.09757854	2.35589204	0.01847829	0.03116338
MAP2K1	709.643991	-0.4751114	0.06719002	-7.0711601	1.54E-12	5.78E-12
MAP2K2	1006.81344	0.07947674	0.053959	1.47290979	0.14077536	0.20572325
MAP3K14	294.979941	-0.953258	0.09968406	-9.5627926	1.15E-21	6.44E-21
MAP3K7	1109.81976	0.24600239	0.05246765	4.68864857	2.75E-06	7.00E-06
MAPK1	1891.08868	0.42452359	0.04061002	10.4536655	1.41E-25	9.07E-25
MAPK13	17.4918499	0.45504073	0.39960014	1.13874015	0.25481155	0.34416106
MAPK14	1023.82207	-0.1314423	0.05449148	-2.4121626	0.01585821	0.02689
MAPK3	271.501803	-0.1172807	0.10274145	-1.1415132	0.25365644	0.34334407
MAPK8	325.75556	0.22254207	0.09772846	2.2771469	0.02277746	0.03810491
MCL1	538.930814	0.5115882	0.07256086	7.05047097	1.78E-12	6.58E-12
MCM2	1165.35326	0.49022723	0.05173547	9.47565035	2.65E-21	1.46E-20
MCM4	3336.69907	0.28676704	0.0320924	8.93566946	4.05E-19	2.00E-18
MDM2	1492.43816	0.5507976	0.04590254	11.9992832	3.58E-33	2.80E-32
MET	1957.85556	0.39045796	0.04216591	9.26003942	2.04E-20	1.06E-19
MGA	344.402098	-0.2339783	0.09022339	-2.5933217	0.00950538	0.01685045
MKI67	4291.75166	0.49952063	0.02914879	17.1369275	7.87E-66	1.36E-64
MLH1	681.220264	0.37243219	0.06453839	5.77070796	7.89E-09	2.45E-08
MLST8	313.763749	-0.0716333	0.09655073	-0.7419243	0.45813321	0.56947235
MMP9	14.634436	0.44757214	0.44639491	1.00263718	0.31603595	0.41869731
MS4A1	15.9751836	0.11191032	0.41646386	0.26871557	0.78814857	0.83983019
MSH2	1547.56319	0.07885645	0.04457676	1.76900355	0.07689328	0.11789044
MSLN	23.4509198	0.16263915	0.34185975	0.47574818	0.63425379	0.72486148
MTHFD2	5108.19666	-0.084537	0.02761491	-3.0612813	0.00220392	0.00425773
MTOR	323.962997	-0.2059304	0.09359747	-2.2001711	0.02779476	0.04600512
MUC1	28.0858784	-0.3125353	0.31917182	-0.979207	0.32747771	0.4302023
MX1	34.463096	0.22603772	0.28451449	0.79446819	0.42692292	0.54115483
MYB	13.8516574	0.58961585	0.44944328	1.31188044	0.18956048	0.26403067
MYBL2	416.606083	0.23036047	0.08355612	2.75695519	0.00583424	0.01073912
MYC	2589.98973	-1.0428314	0.03677427	-28.357641	6.74E-177	2.34E-175
MYD88	389.864057	0.57147564	0.08747491	6.53302324	6.45E-11	2.16E-10
NBL1	67.3897888	0.59906033	0.20332191	2.9463639	0.00321534	0.00604329
NCAPG	239.858771	0.28728294	0.10954658	2.62247293	0.00872942	0.01551897
NCF1	14.3326085	0.3964935	0.43724323	0.90680306	0.36451095	0.469948

NCK1	460.199785	-0.0474622	0.07973565	-0.5952444	0.55168009	0.65572358
NCOA2	272.754514	0.01335648	0.102755	0.12998373	0.8965793	0.92168943
NCOR1	360.642652	-0.083302	0.09224882	-0.9030142	0.36651835	0.47156176
NCR1	20.1537162	-0.3223493	0.37464427	-0.8604144	0.38956066	0.49923099
NDUFA2	2699.17873	-0.1654439	0.03506744	-4.717877	2.38E-06	6.12E-06
NDUFB1	3040.20001	0.05150002	0.03315726	1.55320501	0.12037417	0.18092437
NEIL3	195.361855	0.04714998	0.12521037	0.3765661	0.70649609	0.78304362
NEK2	255.532352	1.11601482	0.11343286	9.83854995	7.68E-23	4.40E-22
NF1	66.1060706	-0.4108389	0.20800304	-1.9751583	0.04825019	0.07779876
NFATC2	18.4022467	-0.7551714	0.39575837	-1.9081627	0.05637019	0.09042415
NFE2L2	1077.02754	0.29279614	0.05286267	5.538807	3.05E-08	8.92E-08
NFKB1	572.421521	-0.2406885	0.07127844	-3.3767368	0.00073351	0.00149579
NFKB2	115.085593	-1.3693562	0.16523734	-8.2872077	1.16E-16	5.28E-16
NKX3-1	29.7722581	-0.2341763	0.30551287	-0.7665022	0.44337756	0.55555743
NME1	8453.89111	0.06752357	0.02286553	2.95307304	0.00314628	0.00593135
NOS3	15.3562149	-0.0723551	0.42957526	-0.1684341	0.86624175	0.90390444
NRAS	2212.8312	0.01497656	0.0390154	0.38386276	0.70108017	0.78019984
NSD1	484.180796	-0.0992573	0.07751672	-1.2804633	0.20038226	0.27663392
NT5E	155.012142	2.73508583	0.17102536	15.9922825	1.45E-57	2.10E-56
NUDT2	406.954755	-0.6077402	0.08403373	-7.232098	4.76E-13	1.83E-12
NUF2	1848.50733	1.36875496	0.04448424	30.7694338	6.72E-208	3.23E-206
OAS1	25.4157101	-0.9142689	0.34692543	-2.635347	0.00840513	0.01498515
OAS3	102.976769	-0.4757753	0.17046748	-2.791003	0.0052545	0.00975835
OSM	17.2133827	-0.1533327	0.40178841	-0.3816254	0.70273924	0.78026564
OSMR	1290.31771	-0.2159529	0.04848257	-4.4542392	8.42E-06	2.05E-05
P4HA2	445.681892	-0.757799	0.08051938	-9.4113861	4.90E-21	2.63E-20
P4HB	5335.4938	-0.783626	0.02677943	-29.26224	3.14E-188	1.40E-186
PA2G4	4285.03346	0.11435986	0.03130715	3.65283481	0.00025936	0.00056986
PAK2	1364.84466	0.50567686	0.04769592	10.6020992	2.91E-26	1.89E-25
PARD3	210.152752	0.38685173	0.119662	3.2328705	0.00122553	0.00244323
PARP1	2282.2789	0.32589294	0.03988123	8.17158608	3.04E-16	1.34E-15
PARP2	678.648942	0.16541853	0.06499928	2.54492861	0.01093001	0.01913328
PARP3	16.3363549	0.76760294	0.42465085	1.8076096	0.07066728	0.1096925
PARVA	440.563893	0.35386411	0.07949863	4.45119768	8.54E-06	2.07E-05
PBRM1	402.501488	0.59178547	0.08543921	6.92639233	4.32E-12	1.54E-11
PCLAF	6725.28299	-0.3875281	0.0250684	-15.458833	6.58E-54	8.73E-53
PCNA	1100.26852	-0.2544948	0.05138063	-4.9531265	7.30E-07	1.91E-06
PDCD1	14.0967414	0.26026036	0.47170701	0.55174156	0.58112543	0.67779864
PDCD1LG2	92.3647242	1.25324176	0.18137166	6.90979917	4.85E-12	1.72E-11
PDGFB	97.9533374	-0.5518865	0.17223631	-3.2042404	0.00135419	0.00269114
PDGFRA	751.849195	-0.4946033	0.06193782	-7.9854817	1.40E-15	5.90E-15
PDK1	332.763756	-0.5846026	0.09325853	-6.2686237	3.64E-10	1.19E-09
PDLIM5	536.561994	-0.0290135	0.07411317	-0.3914761	0.69544535	0.77710903
PDPK1	389.194934	0.03020849	0.08813348	0.34275844	0.7317802	0.79984587
PFKFB3	162.539366	-0.3575958	0.13049768	-2.7402466	0.00613931	0.01116889
PFKP	3171.17509	-0.1909094	0.03310104	-5.7674745	8.05E-09	2.49E-08
PGF	175.329965	-0.5630084	0.12677746	-4.4409185	8.96E-06	2.16E-05
PGK1	28381.587	-0.689987	0.01705134	-40.465273	0	0
PHB	164.500446	-0.4892913	0.13028774	-3.7554673	0.00017302	0.00038558
PIK3CA	786.37517	0.3994987	0.06061387	6.59087937	4.37E-11	1.50E-10
PIK3CB	527.420935	0.1890754	0.0741431	2.55014156	0.01076792	0.01893964
PIK3CD	145.448649	0.21442909	0.13714715	1.56349652	0.11793585	0.17775839
PIK3R1	383.471053	-0.2293077	0.0866296	-2.6469902	0.00812117	0.01452038
PIK3R2	229.063668	0.07410269	0.11527849	0.64281454	0.52034445	0.6304756
PIK3R3	340.98163	0.54499018	0.09259312	5.88586069	3.96E-09	1.25E-08
PIK3R4	406.984423	0.29723399	0.08295895	3.58290443	0.0003398	0.00072863
PKM	40696.2432	-0.6656412	0.01965538	-33.86559	2.14E-251	1.48E-249
PLA2G4A	79.425543	-0.0763061	0.19017785	-0.4012354	0.68824684	0.77103417
PLAU	11055.0007	-0.241405	0.02070051	-11.661794	2.00E-31	1.47E-30
PLCB3	282.084317	0.40579553	0.10126513	4.00725848	6.14E-05	0.00014092
PLK1	3063.04368	0.43892587	0.03296061	13.3166801	1.85E-40	1.65E-39

PMAIP1	1336.53859	0.6269927	0.04824268	12.9966381	1.28E-38	1.08E-37
PNOC	24.3754565	-0.3736938	0.33676257	-1.1096653	0.26714326	0.35771973
POLE	136.796255	-0.0597441	0.14348661	-0.4163738	0.67713649	0.76132103
PPAN	76.3041591	-0.4740088	0.19352561	-2.4493337	0.01431208	0.02433443
PRDX1	12996.4828	-0.7039864	0.0244072	-28.843387	6.13E-183	2.39E-181
PRDX2	4470.6337	0.00229063	0.02783983	0.0822789	0.93442493	0.95587075
PRDX6	12202.473	0.61568393	0.02188156	28.1371111	3.45E-174	1.08E-172
PRIM1	1183.11005	0.10948085	0.05043493	2.17073484	0.02995122	0.04931283
PRKACA	1229.06851	0.11872886	0.0532278	2.23057987	0.02570897	0.04266595
PRKCA	497.519452	-1.0687289	0.08050828	-13.274771	3.24E-40	2.81E-39
PSMB10	41.4497251	0.11066989	0.25572855	0.43276313	0.66518686	0.75605939
PSMB7	2926.44378	0.31140701	0.03378598	9.21704803	3.05E-20	1.57E-19
PSMB9	65.0587609	-2.3092709	0.24557819	-9.403404	5.28E-21	2.82E-20
PTCH1	37.821793	0.75809046	0.27681942	2.73857395	0.00617063	0.01119323
PTCH2	17.3919527	-0.2345744	0.40716471	-0.5761166	0.56453636	0.66717934
PTEN	84.1779821	0.08585725	0.18508546	0.46387894	0.64273448	0.73187284
PTGER1	18.4275688	-0.7025576	0.39253435	-1.789799	0.07348622	0.11322322
PTGER3	15.0907925	-0.5833989	0.4289185	-1.3601627	0.17377845	0.24644944
PTGER4	25.8943053	0.42391168	0.33038465	1.28308531	0.19946219	0.2759743
RAC3	248.482448	0.19975082	0.10594128	1.88548623	0.05936422	0.09473983
RAD51	301.930653	0.65239179	0.09838834	6.63078335	3.34E-11	1.16E-10
RAD51AP1	566.864485	0.52306447	0.07779331	6.72377195	1.77E-11	6.17E-11
RAD54B	494.744239	0.32486671	0.07636506	4.25412745	2.10E-05	4.85E-05
RAD54L	229.292878	0.25105314	0.11082053	2.26540285	0.02348797	0.03918848
RAF1	1169.46331	0.52336011	0.05165993	10.1308724	4.03E-24	2.42E-23
RASA1	751.446852	0.05267599	0.06296754	0.83655788	0.40284113	0.51196103
RB1	1276.29853	0.46864956	0.0495038	9.46694108	2.88E-21	1.56E-20
RB1CC1	1350.36293	-0.1407665	0.04981211	-2.8259488	0.00471408	0.00883359
RBBP5	602.099062	0.54333397	0.07342564	7.39978567	1.36E-13	5.46E-13
RBL1	432.863889	0.10313369	0.08221745	1.25440153	0.2096961	0.28758322
RBX1	2852.44833	0.25910657	0.03517416	7.36639065	1.75E-13	6.97E-13
REL	146.217706	-0.8054789	0.15243149	-5.284203	1.26E-07	3.52E-07
RELB	44.8905498	-0.6324182	0.25061457	-2.5234695	0.01162031	0.0200306
RET	18.0298979	-0.3827494	0.40045729	-0.9557809	0.33918292	0.4409378
RFC2	910.760186	0.46080582	0.05807028	7.93531221	2.10E-15	8.73E-15
RFC3	1313.88255	0.14746574	0.04754369	3.101689	0.0019242	0.00372888
RFC4	1784.97988	1.0226833	0.04440072	23.033033	2.18E-117	5.66E-116
RICTOR	464.313161	-0.0114591	0.07966956	-0.1438332	0.88563219	0.9164751
RIMKLB	199.872666	-0.5207946	0.12607005	-4.1309934	3.61E-05	8.32E-05
RNF111	638.602183	-0.5918476	0.07275541	-8.1347571	4.13E-16	1.80E-15
ROCK1	409.291017	-0.0123207	0.0831928	-0.1480984	0.88226513	0.91450737
ROCK2	726.200608	0.23723239	0.06636925	3.57443224	0.00035099	0.00075006
RPA3	4218.11244	0.55497714	0.02942433	18.8611647	2.38E-79	4.79E-78
RPTOR	64.0121663	0.11151735	0.20789268	0.53641787	0.59166978	0.6875269
RRAGC	283.508074	-0.0813607	0.10431315	-0.7799659	0.43541095	0.54908536
RRM2	6702.52423	0.21012631	0.02615292	8.034527	9.39E-16	4.01E-15
RUNX1	885.933054	0.08271842	0.05844848	1.41523651	0.15699917	0.2252126
RUVBL1	1856.46181	0.61663034	0.04093462	15.0637876	2.80E-51	3.50E-50
SAV1	514.709356	-0.4720216	0.07669836	-6.1542604	7.54E-10	2.43E-09
SDC1	696.979654	0.57484138	0.06475277	8.87747971	6.84E-19	3.33E-18
SELL	19.9166879	-0.6798947	0.40756203	-1.6681993	0.09527618	0.1450057
SERPINB5	16.6086959	-0.6252762	0.42111665	-1.4848052	0.13759543	0.20159138
SERPINE1	3778.97895	-0.4014594	0.03436331	-11.682793	1.56E-31	1.16E-30
SETD2	491.796872	0.34293327	0.08049801	4.26014573	2.04E-05	4.76E-05
SF3B1	2100.63869	0.33284291	0.03898948	8.5367362	1.38E-17	6.53E-17
SFN	142.61923	-4.0746804	0.23459593	-17.368931	1.42E-67	2.53E-66
SH3PXD2A	105.356367	0.42341345	0.16492322	2.5673368	0.0102483	0.01811598
SIGLEC5	17.074139	-0.4100397	0.41631502	-0.9849264	0.3246602	0.42740078
SIN3A	693.481927	-0.5419299	0.0675299	-8.0250361	1.01E-15	4.31E-15
SIRPA	30.4839316	-2.6328515	0.37913987	-6.9442749	3.80E-12	1.36E-11
SKA1	406.049182	0.21382754	0.08407065	2.54342687	0.0109771	0.01913328

SKIL	1290.82292	-0.6561371	0.04957881	-13.234225	5.57E-40	4.76E-39
SKP2	1104.636	0.74683188	0.0541432	13.7936419	2.78E-43	2.67E-42
SLC1A5	3812.38415	-0.1791931	0.03017286	-5.9388815	2.87E-09	9.09E-09
SLC2A1	143.800093	-0.108533	0.14024616	-0.7738751	0.43900464	0.55229616
SLC7A5	1840.83922	-0.7124258	0.04515684	-15.7767	4.50E-56	6.24E-55
SLIT2	297.792469	1.06004913	0.10286445	10.3053016	6.67E-25	4.20E-24
SMAD7	59.1745972	-1.2956199	0.22769411	-5.6901777	1.27E-08	3.84E-08
SMC3	1426.30729	-0.5901978	0.04654861	-12.679174	7.71E-37	6.17E-36
SMO	118.054272	-0.4910449	0.15444418	-3.1794329	0.00147564	0.00292316
SMS	12259.8982	0.13254695	0.02074744	6.38859282	1.67E-10	5.56E-10
SNAI1	27.6300565	-0.4725844	0.31538444	-1.4984393	0.13401915	0.19770201
SNAI2	218.519044	-0.352362	0.11351605	-3.1040719	0.00190877	0.00372137
SOCS1	50.6554706	0.12260514	0.23650468	0.5184047	0.60417594	0.69558263
SOCS3	189.289383	0.3732873	0.12260942	3.04452383	0.00233049	0.00447454
SOD1	1970.61522	-0.4609842	0.04193286	-10.993387	4.11E-28	2.76E-27
SORD	893.941863	-0.8651621	0.06300314	-13.732049	6.53E-43	6.17E-42
SOS1	322.413305	-0.740692	0.10494591	-7.0578459	1.69E-12	6.28E-12
SOX2	833.107936	0.16620458	0.06042711	2.75049706	0.00595049	0.01085704
SOX7	30.8589788	0.09740909	0.30793364	0.31633144	0.75175095	0.8158132
SPHK2	31.307252	0.23556119	0.308804	0.76281781	0.44557206	0.55718831
SPINT1	32.6884437	1.46172133	0.31046528	4.70816364	2.50E-06	6.39E-06
SPINT2	19.2307201	-0.699371	0.39072705	-1.7899221	0.07346643	0.11322322
SQLE	592.083912	-0.4316158	0.06946761	-6.2131945	5.19E-10	1.69E-09
SQSTM1	1826.53051	-0.3294799	0.04553382	-7.2359391	4.62E-13	1.79E-12
SRC	23.0261509	-0.439984	0.35935213	-1.2243811	0.22080849	0.3014978
SREBF1	4950.1316	-0.1622667	0.02826573	-5.7407577	9.43E-09	2.88E-08
SRM	1993.77172	-0.2120201	0.03913984	-5.4169899	6.06E-08	1.74E-07
SSRP1	2896.65513	0.3365318	0.03553693	9.46991793	2.80E-21	1.53E-20
STAG2	1401.15152	-0.0309677	0.04696469	-0.6593833	0.50964965	0.62161457
STAT1	528.265374	0.26077262	0.07695893	3.3884649	0.00070285	0.00144745
STAT3	2015.56349	-0.0118381	0.03952823	-0.299485	0.76457002	0.82399256
STAT4	13.9729154	0.69453434	0.46779627	1.48469407	0.13762488	0.20159138
STING1	197.872256	0.68146927	0.1271392	5.36002496	8.32E-08	2.37E-07
STIP1	5186.50492	0.5178177	0.03145006	16.4647585	6.57E-61	1.03E-59
STK3	198.223184	0.80784042	0.12257955	6.59033617	4.39E-11	1.50E-10
STK4	419.117617	-0.4246585	0.08486845	-5.0037262	5.62E-07	1.49E-06
SUPT16H	919.709015	0.03562835	0.05732076	0.62156102	0.53423056	0.64479665
SYK	14.5796183	0.08990997	0.43919379	0.20471593	0.83779407	0.87715353
TAP1	142.33377	-1.3481126	0.14782568	-9.11961	7.54E-20	3.82E-19
TAP2	241.372075	-0.6382914	0.10878513	-5.8674508	4.43E-09	1.39E-08
TAPBP	212.310614	-0.6657238	0.11910558	-5.5893583	2.28E-08	6.74E-08
TAZ	135.044061	-0.5361443	0.14335655	-3.7399362	0.00018407	0.00040875
TBRG4	509.826716	0.07157867	0.0754838	0.94826529	0.34299442	0.44496573
TBX21	14.8493593	0.15061736	0.43070839	0.34969682	0.72656624	0.79679672
TCF7	26.2363178	0.34427183	0.32869847	1.04737886	0.29492488	0.39239472
TCL1A	14.030193	7.96E-05	0.44867871	0.00017744	0.99985843	0.99985843
TCOF1	203.207769	0.09445087	0.11904628	0.79339623	0.42754701	0.54115483
TEAD1	811.860765	0.21998648	0.05948664	3.69808214	0.00021724	0.00048069
TEAD2	192.459754	0.4521833	0.12565564	3.59859128	0.00031995	0.00069082
TERF2IP	275.385789	-0.0135405	0.10109057	-0.1339445	0.89344644	0.91998446
TERT	22.702406	-0.7930889	0.37877518	-2.0938248	0.03627559	0.05925645
TET2	373.697965	0.13372604	0.08886584	1.50480817	0.1323734	0.19573698
TFDP1	523.649584	0.11772755	0.08075634	1.45781194	0.14489239	0.21075256
TGFB1	730.765364	-1.33713	0.06710619	-19.925584	2.44E-88	5.25E-87
TGFB2	906.612944	1.26494787	0.06152088	20.5612773	6.10E-94	1.52E-92
TGFB3	65.1129412	-2.020466	0.23627245	-8.5514242	1.22E-17	5.79E-17
TGFBR2	957.854229	1.06690505	0.05707603	18.6926982	5.68E-78	1.11E-76
THBS1	5821.3831	1.64615752	0.0282364	58.2991351	0	0
THBS2	532.461735	2.65976127	0.09442834	28.1669827	1.48E-174	4.88E-173
TIGIT	17.4627434	0.01995237	0.41322295	0.04828476	0.9614893	0.97076813
TIMELESS	897.854055	0.01726604	0.05677702	0.30410258	0.76104974	0.82304166

TJP1	2717.02948	-0.7528337	0.0374899	-20.080971	1.08E-89	2.41E-88
TLR3	32.8380933	-1.0072967	0.29983981	-3.3594495	0.00078098	0.0015874
TLR4	705.25112	1.07825869	0.0676087	15.94852	2.92E-57	4.14E-56
TMPRSS2	14.4774367	0.02621004	0.44665957	0.05868013	0.95320689	0.9688634
TNF	16.8937799	-0.3768135	0.4036031	-0.933624	0.35049786	0.45281712
TNFAIP3	34.4899713	-0.7167759	0.28459904	-2.5185465	0.01178403	0.02025685
TNFRSF17	14.7079178	-0.4596125	0.44037781	-1.0436776	0.29663457	0.39382972
TNFRSF18	17.6662376	-0.1204384	0.39202717	-0.3072195	0.7586763	0.82189933
TNFRSF4	28.1333947	-0.208758	0.32945554	-0.6336454	0.52631228	0.63647066
TNFRSF9	31.5581945	-0.8474348	0.30256589	-2.8008271	0.00509718	0.00949445
TNFSF11	21.15803	0.09774584	0.36470095	0.26801641	0.78868669	0.83983019
TOP2A	7207.81527	0.50580617	0.02514148	20.1183913	5.09E-90	1.18E-88
TOX	591.426559	-0.7501285	0.0729072	-10.288812	7.91E-25	4.94E-24
TOX3	146.250331	1.01443031	0.14365586	7.06153084	1.65E-12	6.15E-12
TOX4	676.658809	0.17257299	0.06806077	2.53557198	0.01122639	0.01945907
TP53	1389.79268	0.43935416	0.04672852	9.4022696	5.34E-21	2.82E-20
TP73	45.5145426	2.49293956	0.30131122	8.27363667	1.30E-16	5.88E-16
TPD52	1272.8241	-0.2563473	0.04902582	-5.228823	1.71E-07	4.71E-07
TPI1	4996.02737	-0.3023599	0.02721888	-11.108459	1.14E-28	7.91E-28
TPX2	2792.00353	0.19319443	0.0339867	5.6844121	1.31E-08	3.96E-08
TRAF1	21.5163819	-0.083646	0.3590701	-0.2329516	0.81579898	0.86071255
TRAF6	285.821273	-0.1772513	0.09934852	-1.7841366	0.07440147	0.11435103
TRRAP	652.896664	-0.0383277	0.07270332	-0.527179	0.59806929	0.69367145
TSPAN1	26.3784773	0.6259258	0.33414364	1.87322373	0.0610375	0.09642379
TTK	687.391315	0.4860746	0.06526034	7.44823886	9.46E-14	3.81E-13
TUBG1	741.304085	0.09969479	0.06522577	1.52845707	0.12639909	0.18734688
TXN	7085.00353	0.44527956	0.02615439	17.0250427	5.36E-65	9.03E-64
TXN2	1161.38232	-0.1454042	0.05277769	-2.7550311	0.00586866	0.01077071
TXNRD2	278.751544	0.13058329	0.10006822	1.30494264	0.19191238	0.26671119
TYROBP	22.4613066	-0.1255541	0.34961668	-0.3591193	0.71950583	0.79044303
UBE2C	3415.62882	-0.0520055	0.03362807	-1.5464902	0.12198624	0.18210385
UBE2T	274.162146	0.89899872	0.10713571	8.39121446	4.81E-17	2.21E-16
UCHL5	1380.54374	0.80233552	0.04728837	16.9668691	1.44E-64	2.31E-63
UGDH	666.933864	-0.709603	0.06597075	-10.756327	5.53E-27	3.67E-26
VAV1	13.9472399	0.02453405	0.46580569	0.05267013	0.95799474	0.9688634
VCP	5511.49888	-0.7607028	0.02675863	-28.42832	9.03E-178	3.32E-176
VEGFA	355.213452	-1.540415	0.10039289	-15.343866	3.89E-53	5.06E-52
VEGFB	294.89822	0.07625668	0.09780366	0.77969144	0.43557252	0.54908536
VEGFC	142.858757	-0.1981602	0.13887868	-1.4268583	0.15362073	0.22087405
WASF2	1329.02017	-0.102593	0.04916247	-2.0868158	0.03690479	0.0600012
WEE1	1279.26571	-0.3511168	0.0487207	-7.2067285	5.73E-13	2.19E-12
WNT5A	308.543338	-1.0866228	0.10166145	-10.688643	1.15E-26	7.56E-26
WRAP53	417.122563	-0.020065	0.08149446	-0.2462131	0.80551726	0.85483465
WWC1	380.336929	-0.5496076	0.08713833	-6.3073002	2.84E-10	9.37E-10
WWTR1	2183.9591	0.18009071	0.03817155	4.71793014	2.38E-06	6.12E-06
XBP1	577.524821	0.25703321	0.07287738	3.52692712	0.00042041	0.00088928
XRCC2	373.005184	-0.4805519	0.08857048	-5.425644	5.77E-08	1.67E-07
YAP1	666.500119	0.22625053	0.06687922	3.38297199	0.00071706	0.00146703
ZEB1	221.667669	-0.609425	0.1138434	-5.3531865	8.64E-08	2.45E-07
ZMYM2	368.163418	-0.4379206	0.08817737	-4.9663604	6.82E-07	1.79E-06
ABCF1	956.697353	0.23599837	0.05543319	4.25734775	2.07E-05	4.80E-05
DNAJC14	213.604431	-0.0381993	0.11880815	-0.3215205	0.74781599	0.81437552
ERCC3	1347.31952	0.27354144	0.04786988	5.71426983	1.10E-08	3.35E-08
G6PD	1592.9585	-0.641792	0.04398437	-14.591365	3.19E-48	3.83E-47
GUSB	198.757903	0.00713027	0.12667196	0.05628926	0.95511138	0.9688634
MRPL19	1508.37212	-0.2262482	0.04490605	-5.038257	4.70E-07	1.26E-06
NRDE2	231.497714	0.14727934	0.10869166	1.35501971	0.17541128	0.24802179
OAZ1	10869.4454	-0.1491979	0.02118013	-7.0442375	1.86E-12	6.80E-12
POLR2A	530.674199	0.08602566	0.07606504	1.13094871	0.25807668	0.34781825
PSMC4	992.81467	-0.2067041	0.05634261	-3.6686999	0.00024379	0.00053754
PUM1	830.179899	-0.1254883	0.0616897	-2.0341859	0.04193286	0.06778783

SDHA	537.996911	0.19842595	0.07372605	2.69139561	0.00711538	0.01279537
SF3A1	1060.21481	-0.0296778	0.05257209	-0.5645168	0.57240247	0.67079249
STK11IP	118.415257	-0.2445592	0.15943428	-1.5339183	0.12504976	0.18590902
TBC1D10B	145.431879	0.0036765	0.1390923	0.02643209	0.9789127	0.98364175
TBP	770.865363	0.15194781	0.06177141	2.45984061	0.01389987	0.02369814
TFRC	3684.7449	-0.4184418	0.03055447	-13.694946	1.09E-42	1.01E-41
TLK2	633.982475	-0.1838147	0.0681457	-2.6973775	0.0069888	0.01260408
TMUB2	257.876967	0.13950093	0.11006217	1.26747397	0.20498587	0.28236464
UBB	26583.5048	-0.2047575	0.01759912	-11.63453	2.75E-31	2.00E-30
NEG_F	14.1620528	0.09482925	0.44299744	0.21406276	0.83049812	0.87118263
POS_A	17738.0436	0.08672648	0.04625508	1.87496135	0.06079804	0.09628928
POS_B	7281.63072	0.08954272	0.02534508	3.53294247	0.00041096	0.00087225
POS_C	2047.13572	0.12054396	0.04727374	2.54991374	0.01077496	0.01893964
POS_D	448.207761	0.04948802	0.08273667	0.5981389	0.54974725	0.65572358
POS_E	90.3639927	0.10840866	0.18923414	0.57288109	0.56672521	0.66723874
POS_F	37.3285227	-0.114699	0.26942409	-0.425719	0.6703126	0.75637444

Immune Cell	$CD97^{hi}$ Proportion	$CD97^{lo}$ Proportion	*p* -value
Monocytes	0.069	0.109	<0.001*
M0 Macrophages	0.07	0.034	0.021*
M1 Macrophages	0.017	0.015	0.698
M2 Macrophages	0.476	0.489	0.432
Regulatory T Cells	0.017	0.008	0.021*
CD8+ T Cells	0.031	0.027	0.652
CD4+ Naïve T Cells	0.0002	0.0009	0.224
CD4+ Resting Memory T Cells	0.11	0.098	0.37
CD4+ Activated Memory T Cells	0.003	0.001	0.409
Plasma Cells	0.035	0.039	0.33
Resting NK Cells	0.044	0.029	0.030*
Activated NK Cells	0.018	0.025	0.116

Supplementary Table S4. Proportion of tumor-infiltrating immune cells in $CD97^{hi}$ and $CD97^{lo}$ tumors. *p <0.05
